# Innovative Bayesian and Parsimony Phylogeny of Dung Beetles (Coleoptera, Scarabaeidae, Scarabaeinae) Enhanced by Ontology-Based Partitioning of Morphological Characters

**DOI:** 10.1371/journal.pone.0116671

**Published:** 2015-03-17

**Authors:** Sergei Tarasov, François Génier

**Affiliations:** 1 Department of Research and Collections, National Center for Biosystematics, Natural History Museum University of Oslo, P.O. Box 1172 Blindern NO-0318, Oslo, Norway; 2 Coleoptera Section, Canadian National Collection of Insects, Arachnids and Nematodes, Agriculture and Agri-Food Canada, 960 Carling Avenue, Ottawa, K1A 0C6, Ontario, Canada; Onderstepoort Veterinary Institute, SOUTH AFRICA

## Abstract

Scarabaeine dung beetles are the dominant dung feeding group of insects and are widely used as model organisms in conservation, ecology and developmental biology. Due to the conflicts among 13 recently published phylogenies dealing with the higher-level relationships of dung beetles, the phylogeny of this lineage remains largely unresolved. In this study, we conduct rigorous phylogenetic analyses of dung beetles, based on an unprecedented taxon sample (110 taxa) and detailed investigation of morphology (205 characters). We provide the description of morphology and thoroughly illustrate the used characters. Along with parsimony, traditionally used in the analysis of morphological data, we also apply the Bayesian method with a novel approach that uses anatomy ontology for matrix partitioning. This approach allows for heterogeneity in evolutionary rates among characters from different anatomical regions. Anatomy ontology generates a number of parameter-partition schemes which we compare using Bayes factor. We also test the effect of inclusion of autapomorphies in the morphological analysis, which hitherto has not been examined. Generally, schemes with more parameters were favored in the Bayesian comparison suggesting that characters located on different body regions evolve at different rates and that partitioning of the data matrix using anatomy ontology is reasonable; however, trees from the parsimony and all the Bayesian analyses were quite consistent. The hypothesized phylogeny reveals many novel clades and provides additional support for some clades recovered in previous analyses. Our results provide a solid basis for a new classification of dung beetles, in which the taxonomic limits of the tribes Dichotomiini, Deltochilini and Coprini are restricted and many new tribes must be described. Based on the consistency of the phylogeny with biogeography, we speculate that dung beetles may have originated in the Mesozoic contrary to the traditional view pointing to a Cenozoic origin.

## Introduction

**Fig 1:**
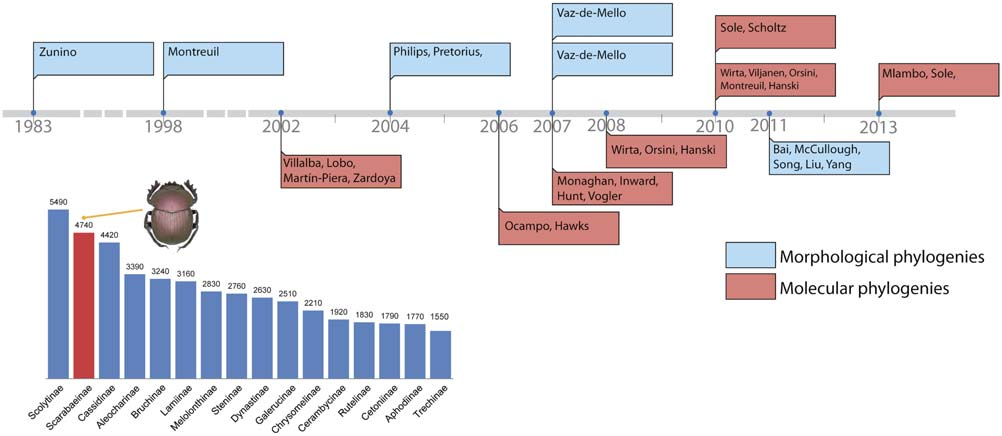
Phylogenetic timeline and citation chart. On the top: phylogenetic timeline illustrating history of phylogenetic studies in dung beetles. Description of the phylogenetic studies is provided in Table 1. Left on the bottom: citation chart of Coleoptera subfamilies. The popularity of each Coleoptera subfamily is based on the number of citations on Google Scholar. The diagram shows the 16 most popular Coleoptera subfamilies whose number of citations exceed 1500. The citations were obtained by querying a recently updated list of all Coleoptera subfamilies (provided by Alfred Newton, Field Museum, Chicago, IL) in Google Scholar using R script (available upon request). Scarabaeinae emerges as the second top subfamily, with 4740 citations. Numbers above columns indicate the number of citations.

Dung beetles of the subfamily Scarabaeinae are a well-known group of insects thanks to their exploitation of animal feces, a behavioral trait with a global impact on Earth’s ecosystems. With more than 6200 species in 267 genera and with an estimated 30–50% of the species still undescribed (FG, personal database), dung beetles exhibit diversity comparable to the Aves (ca. 9.800 species [1]), as well as being the second most cited subfamily of beetles on Google Scholar (Fig. 1). Dung beetles have been shown to play a major role in nutrient recycling, bioturbation, enhancement of plant growth, secondary seed dispersal, parasite suppression and dispersal, fly control, trophic regulation and pollination [2]. Through these various mechanisms, they provide ecosystem services valued at $380 million annually for the cattle industry in the US alone [3].

Their association with ephemeral food resources coupled with interesting and complex feeding, breeding and nesting behaviors make dung beetles a model for various ecological studies [4]. Dung beetles have been selected as indicators for biodiversity inventory and monitoring due to their global distribution, high abundance, ease of capture combined with tight connections to specific soil and vegetation types; their sensitivity to community disturbance allows assessment of human impact around the globe [4, 5]. Dung beetles, amongst few other invertebrate taxa, were selected for the IUCN Red List Index program [6, 7]. The scarabaeine genus *Onthophagus*, one of the most species-rich genera in animal kingdom, in which horns exhibit fascinating phenotypic diversity, has emerged as a model system in evolutionary developmental biology and ecological development [4, 8].

Due to the extensive biological knowledge acquired about them and their unique biological traits, dung beetles stand out amongst other invertebrates, as organisms ideal for unraveling general patterns of nature. Dobzhansky’s famous "Nothing in biology makes sense except in the light of evolution" [9] applies well as comparative data in biology can be only interpreted if we know the phylogeny of the organisms under study. Dung beetles’ all-around popularity in the last two decades has made them the subject of 13 key phylogenetic studies aiming to resolve their evolutionary history using both molecular and morphological characters (Fig. 1, Table 1). Despite such intense scrutiny, the phylogeny of dung beetles remains largely unresolved. Current phylogenies often resolve only very shallow divergences between closely related genera and largely disagree with each other concerning deeper relationships (see next chapter for details). As a result, we still do not have a firm basis for comparative evolutionary and ecological research and a good natural classification for this important group.

**Table 1:** The key phylogenetic studies in the subfamily Scarabaeinae. See also phylogenetic time-line in Fig. 1.

#	Year	Authors	Description
1	2013	Mlambo et al. [29]	MOLECULAR: 48 species, COI, 16S, 28Sd3; majority of African Deltochilini and Dichotomiini, 5 Madagascan and 1 Oriental taxon
2	2011	Bai et al. [23]	MORPHOLOGY: 81 species 119 characters; Chinese taxa
3	2010	Sole and Scholtz [28]	MOLECULAR: 35 species, COI, 16S, 28Sd2, 28d3, CAD; majority of African genera
4	2010	Wirta et al. [27]	MOLECULAR: 155 species, COI, 16S, 18S, 28S, COI; focused on Madagascan Deltochilini but also includes many other Scarabaeinae taxa
5	2008	Wirta et al. [26]	MOLECULAR: 44 species, COI, 18S, 28S, COI, Cytb, 16S; focused on Madagascan tribe Helictopleurini but also includes other genera of Scarabaeinae
6	2007	Monaghan et al. [25]	MOLECULAR: 224 species, COI, 16S, 28S; global coverage of Scarabaeinae
7	2007	Vaz-de-Mello [22]	MORPHOLOGY: 12 species, 20 characters; some Neotropical genera of Dichotomiini and Deltochilini
8	2007	Vaz-de-Mello [11]	MORPHOLOGY: 87 taxa, 297 characters; global coverage of Scarabaeinae
9	2006	Ocampo and Hawks [24]	MOLECULAR: 45 species, 18S, 28Sd2, 28d3, various Scarabaeinae genera
10	2004	Philips et al. [20]	MORPHOLOGY: 48 species, 199 characters; 47 genera from various biogeographic regions
11	2002	Villalba et al. [18]	MOLECULAR: 33 species, COI, COII; Iberian species
12	1998	Montreuil [17]	MORPHOLOGY: 29 genera, 42 characters; some Dichotomiini and Deltochilini
13	1983	Zunino [19]	MORPHOLOGY: 18 genera from various biogeographic regions; intuitive cladistic approach

Usually molecular phylogenies dealing with dung beetles rely on a limited number of mitochondrial (both protein-coding and rDNA) and nuclear rDNA markers. Protein-coding mitochondrial markers, affected by faster evolution, are less suitable for uncovering deeper divergences, while rDNA sequences pose alignment problems and challenge modeling of their evolution [10]. Although mitochondrial and rDNA genes are good candidates for resolving shallow divergences, they seem to include noise and less information for elucidating relationships at a higher level [10]. Morphological phylogenies are sensitive to taxon sampling that can significantly affect their results. On average, morphological studies in dung beetles use up to 50 terminals; there is only one study comprising 80 taxa and a representative sample of the genera [11]. In addition, morphological phylogenies, to a certain extent, lack a thorough investigation of morphology that would include total examination of their internal and external structures as well as genitalia. Such in-depth investigation is critical as morphological phylogenies strictly depend on homology assessment and incorporation of all possible phylogenetic information preserved in the phenotype.

In this paper, we aim at a reconstruction of dung beetle phylogeny based on an unprecedented analysis in terms of taxon sample and depth of morphological investigation.

In the current era of phylogenomics, our choice of a morphological approach is based upon two considerations rendering morphology irreplaceable. First, even big multi-gene studies may provide biased estimates and perform worse than morphology [12]. Thus, morphology is an alternative source of data important for the assessment of phylogenetic accuracy. Second, morphology is essential to the development of a new robust natural classification for dung beetles, which requires morphological characters for diagnosis and identification of taxa.

The present study has several key advantages over previous morphological studies. It covers all biogeographic and morphological diversity and incorporates the majority of putative lineages, and suprageneric categories considered phylogenetically questionable. We performed the most detailed investigation of endo- and exoskeleton structures in dung beetles using a dissecting technique described in the materials and methods section. We specially focus on studying the internal sclerites of male genitalia. These structures recently have been shown to be phylogenetically informative [13], although remaining poorly studied as their preparation and examination is rather complex. We provide the first large scale assessment of homology for internal sclerites and their detailed illustration. Our thorough examination of morphology revealed many novel characters, which are informative for uncovering phylogenetic relationships in dung beetles.

Complementary to the parsimony approach prevailing in phylogenetic inference using morphology, we also use a Bayesian method. Previous studies applying Bayesian methodology to morphology revealed that, although results of the Bayesian and parsimony analyses are similar, the Bayesian approach might infer interesting patterns neglected by parsimony [14–16]. Bayesian inference is based on substitution models, accounts for rate heterogeneity and can incorporate autapomorphic characters, ignored by parsimony. By partitioning the dataset, model-based methods allow different partitions to evolve at different evolutionary rates. Such an approach seems biologically more realistic than parsimony, and therefore can be interesting for phylogenetic inference. However, the partitioning of the morphological datasets, unlike the molecular ones, is not straightforward. Here, we use anatomic ontology i.e., anatomical relationships among characters for partitioning. This approach assigns characters in partition given their anatomical location on the beetle body, on the assumption that characters of the same anatomical region undergo similar evolutionary dynamics. The dataset can be partitioned in multiple ways that, also allows testing hypotheses about character evolution. We use Bayes factor to elucidate the best partitioning scheme for phylogenetic inference and answer the following biological questions. *Is partitioning of the morphological dataset meaningful*? *Do characters on the same anatomical region evolve at similar rates*? *And*, *how can autapomorphic characters affect tree topology*?

### How many patterns are out there: a review of Scarabaeinae phylogenies

The current classification splits the subfamily Scarabaeinae into 12 tribes and approximately 267 genera. More than half of the genera are classified in the tribes Deltochilini (formerly Canthonini, 103 genera) and Dichotomiini (43 genera). All phylogenetic studies support the polyphyly of these tribes, which results in a constant shuffling of genera among Deltochilini, Dichotomiini and the other tribes. Of all the tribes, Deltochilini, Dichotomiini, Coprini, and Onthophagini have a global distribution. Despite that, the tribes Deltochilini, Dichotomiini do not share any genera among the New World, Old World and Australasian Regions. The tribes Gymnopleurini, Scarabaeini and Onitini are endemic to the Old World, while Eucraniini, Eurysternini and Phanaeini are endemic to the New World. Two tribes, Oniticellini and Sisyphini, occur in both the Old and New World, but their New World presence likely represents rather recent dispersal. Interestingly, the Australasian Region, known for its high generic endemism, lacks any currently recognized endemic dung beetle tribes [4].

The first study of dung beetle phylogeny, based on an intuitive phylogenetic approach and illustrated by a tree drawn by hand, dates back three decades [35]. Then, after a fifteen years hiatus, Montreuil [17] published the first morphological phylogeny based on parsimony, with a dataset comprising 29 genera mostly from the tribes Coprini, Deltochilini and Dichotomiini and using 42 characters. Four years later, the first molecular phylogeny [18] using two gene regions (COI and COII) and focusing on Iberian dung beetles was published. This publication started an ongoing and still increasing interest in molecular and morphological investigation of dung beetles resulting in a steady flow of publications every year or two. Up to date, 7 morphology-based [11, 17, 19–23] and 6 molecular-based studies [18, 24–29] relevant to higher-level phylogeny of Scarabaeinae have been published. A summary of these publications (Table 1 and Fig. 1) is presented as a historical timeline that can be found at http://embed.verite.co/timeline/?source=0Aqe3bAUelOltdG9ac1VEdmczbXdUSGdwSWU5QVF2T2c. Although, these phylogenies pursue different goals and differ in taxonomic content and biogeographic coverage as well as the set of molecular or morphological markers used, we can conduct comparisons between them, due to the significant taxon overlap.

The largest studies in terms of biogeographic coverage and taxon sample are the morphological phylogeny of Vaz-de-Mello [11] (87 species, 297 characters) and the molecular phylogeny of Monaghan et al. [25] (224 species, 3 gene regions). Some of the other phylogenies focus mainly on specific biogeographic regions; e.g., [24] Neotropical taxa; [28, 29] Deltochilini and Dichotomiini genera from Africa and Madagascar [26, 27].

Molecular phylogenies use an almost uniform set of 2–6 gene regions which represent mitochondrial protein-coding gene(s) and nuclear and/or mitochondrial rDNA genes. A single broad-scale study attempted incorporating nuclear protein-coding markers [28]. Nuclear markers were shown to be highly informative for a smaller-scale phylogeny dealing with taxa of Onthophagini [30].

The substantial disagreement between the 13 key phylogenies complicates induction of shared patterns, as almost every single study has its unique tree. Here, we highlight the most critical cases and summarize patterns shared between two or more phylogenies or those with interesting biogeographical correlations. Each analysis is brief on purpose in order to demonstrate the dramatic incongruence of the results. Scholtz et al. [4] provide an excellent in-depth review of existing phylogenies.

**Fig 2:**
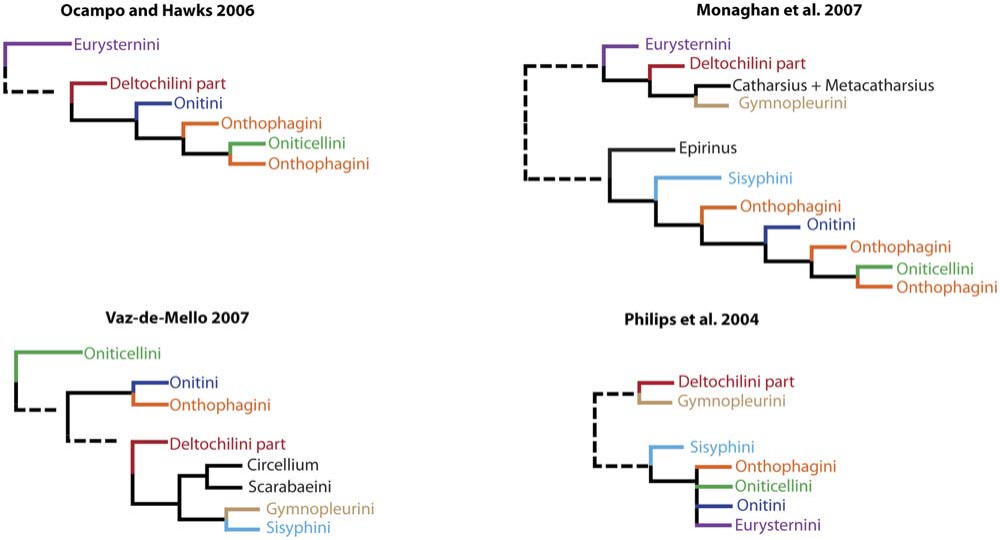
Phylogenetic patterns revealed by previous phylogenies. The trees illustrate relationships, among tribes Onthophagini, Oniticellini, Onitini, Sisyphini, Eurysternini, Deltochilini and some genera. Interrupted line indicates remote relationship between clades i.e., it omits irrelevant taxa/clades branching between the clades it joins.

All phylogenies support monophyly of Scarabaeinae. The majority of phylogenies do not challenge the monophyly of 8 (Onitini, Sisyphini, Gymnopleurini, Scarabaeini, Eurysternini, Oniticellini, Phanaeini, Eucraniini) out of 12 scarabaeine tribes, while two tribes Dichotomiini and Deltochilini, emerge as highly polyphyletic. Despite these similarities, substantial discrepancies appear in the relationships between monophyletic tribes (Fig. 2) and genera of the polyphyletic Dichotomiini and Deltochilini.

To exemplify, two molecular phylogenies [24, 25] recover the tribe Oniticellini nested within the tribe Onthophagini, another phylogeny based on morphology recovers polytomy comprising Onthophagini and Oniticellini [24], while the other morphological phylogeny [11] recovers Oniticellini as a basal lineage on the Scarabaeinae tree, only remotely related to Onthophagini (Fig. 2). At the same time, there is also a lack of consensus on the monophyly of the tribe Onthophagini that emerges polyphyletic or paraphyletic in broad-scale molecular phylogenies [24, 25], or comes up monophyletic in one morphological phylogeny [13].

The phylogenetic positions of the tribes Gymnopleurini and Eurysternini have been in constant flux among phylogenies as they support different placements of these two tribes as demonstrated in Fig. 2.

The studies with sufficient taxon sampling to test the monophyly of Coprini, uncover its polyphyletic nature: two morphological phylogenies [17, 20] separate Coprini into two lineages, while a molecular and another morphological phylogeny separate Coprini into three lineages [11, 25]. Additionally, all four phylogenies revealing polyphyly contradict each other in the affiliation of separate Coprini lineages.

Two molecular phylogenies [24, 25] support monophyly of Neotropical tribes Phanaeini + Eucraniini and their sister relationship to the Neotropical genus *Dichotomius* and its close relatives. Conversely, two morphological phylogenies are not consistent with this pattern, and place Eucraniini as sister to *Circellium* + Scarabaeini [20] or recover a clade composed of Onitini + Onthophagini within the clade Phanaeini + Eucraniini [11].

Many phylogenies are consistent in the position of the genera *Sarophorus*, *Frankenbergerius*, *Coptorhina*, *Dicranocara*, *Odontoloma* and allies which emerge as basal or nearly basal lineages [11, 25, 28, 29]; however relationships between these genera differ among those studies.

Two morphological [11, 20] and molecular phylogenies [25] confirm the sister relationship between *Circellium* (or *Circellium + Ateuchus*) and the tribe Scarabaeini. However, two other molecular phylogenies place *Circellium* as either sister of *Pedaria* [28] or belonging to a clade consisting of the genera *Janssensantus + Tanzanolus* [29].

The molecular phylogeny of Monaghan et al. [25] recovers an interesting clade comprising almost all Australasian endemic genera with New Zealand, New Caledonia, and one African genus *Pedaria* nested within. Only two Australasian genera, *Boletoscapter* and *Monoplistes*, not falling in the Australasian clade, break the monophyly of Australasian endemic genera in that phylogeny. That study also recovers monophyly for New Caledonian genera. Such consistency related to geography makes this phylogenetic pattern quite meaningful biogeographically, although not supported by any other phylogenetic study.

The evolutionary scenario for the majority of Madagascan taxa is also contradictory. According to [27], Deltochilini includes three separate lineages on Madagascar: *Arachnodes + Epilissus*, *Epactoides*, and *Nanos + Apotolamprus*. The *Epactoides* lineage is sister to the Oriental genus *Ochicanthon*, with whom they form a clade sister to the Australasian genus *Monoplistes*; this Australasian—Oriental—Madagascan clade, in turn, is sister to another Madagascan lineage comprising *Nanos + Apotolamprus*. However, the latter bigger clade is weakly supported in that analysis (posterior probability 0.54). The sister group to the lineage *Arachnodes + Epilissus* was not inferred in that study as this lineage ends in a polytomy with many other taxa used in the study. Simultaneous analysis of Madagascan and Afrotropical taxa [29] corroborates the division of Madagascan scarabaeines [27] into three lineages, and, additionally, places the Eastern-Arc mountain genus *Madaphacosoma* within the *Epactoides + Ochicanthon* lineage. However, the largest molecular phylogeny [25] merges all three Madagascan lineages along with *Ochicanthon* and *Monoplistes* in one clade, and at the same time places *Epilissus splendidus* Fairmaire, 1889 and *Arachnodes sp*. as sister to the Neotropical genus *Trichilium*.

## Materials and Methods

### Outline of the methodological procedure

To construct a character matrix for the phylogenetic analyses, we performed detailed examination of endo- and exoskeleton morphology (205 characters) for the selected dung beetle taxa (110 species). To make our statements of morphological characters clear and usable in further research we document them by providing illustrations of almost all characters used (see character report and illustrations).

To infer phylogenetic relationships we ran six parsimony analyses (parsimony analyses section). In addition to them, we ran 184 Bayesian analyses using different schemes of character matrix partitioning to infer phylogenetic trees and best partitioning scheme (Bayesian analyses section). The partitioning of the matrix is based on our innovative approach that uses anatomy ontology to guide the partitioning (anatomy ontology construction section).

### Illustrations and abbreviations

Photos were taken with Canon EOS 500D digital camera attached to a Leica MZ16 microscope. Aedeagi, endophallic sclerites and some skeletal structures were photographed in an alcohol-based hand sanitizer comprising a dense gel that was used to fix the position of structures for photography. The color schemes of the endophallic sclerites were drawn in Adobe Illustrator. The technical notes on the illustrations are provided in character report section. Abbreviations used in the text and figures are detailed in Table 2.

**Table 2:** Abbreviations used in the text.

**?**	indicates sclerites with unclear homology
**AcS**	accessory sclerites
**AIS**	additional inferior sclerite
**AMS**	additional medial sclerite of lamella copulatrix
**AS**	additional sclerite
**ADP**	anterior depression of propleura
**ARP**	anterior ridge of propleuron
**A**	axial sclerite
**BSG**	basal sclerite of galea
**BS**	basal sclerite of internal sac
**BSSg**	basal sclerite spiculum gastrale
**BSc**	basal semicircular sclerite
**BFP**	basisternal furca of prothorax
**Blp**	basolateral paramerite
**Blp**	basolateral plate
**BShA**	border delimiting area of head shielded by pronotum
**Br**	bristle
**Cav**	cavity
**CRP**	clypeal rectangular pattern
**CxDp**	coxal depression
**DASG**	dorsal articular sclerite of galea
**DPT**	dorsal process of tentorium
**ExL**	external lobe
**Eye**	eye
FLP*	fronto-lateral peripheral sclerite marked with * is not considered to be homologous to FLP
**FLP, FLP1, FLP2**	fronto-lateral peripheral sclerite, fronto-lateral peripheral sclerite 1 (outer), fronto-lateral peripheral sclerite 2 (inner).
**Gl**	glossa
**GlFl**	glossal flap
**Gr**	groove
**InLM**	incisor lobe of mandibles
**IAS1, IAS2**	inferior accessory sclerite 1 and 2 respectively
**IL**	inferior left lobe of lamella copulatrix
**ILb**	inferior lobe
**IP**	inferior portion
**IR**	inferior right lobe of lamella copulatrix
**ICP**	internal carina of pronotum
**InL**	internal lobe
**InS**	internal sack
**LC**	lamella copulatrix
**LlS**	lateral labial sclerite
**LP**	lateral process
**MP**	medial peripheral sclerite
**MsArRp**	mesofurcal arm, rear process
**MtSc**	metascutellum
**MtEp**	metepisternum
**PIS**	parameral inferior side
**PSS**	parameral superior side
**PhFS**	phallobase frontal side
**PhRS**	phallobase rear side
**PhG**	pheromone gland
**pit**	pit
**Pit**	pit of different origin
**PRP**	posterior ridge of propleuron
**Pr**	projection
**PsnSt**	prosternal sternellum
**Ri**	ridge
**X**	sclerite of unknown homology
**SclP**	sclerotized plate of parameral inferior side
**SclR**	sclerotized roof
**ScP**	scutellum plate
**ScAP**	scutellum, apical process
**setae**	setae
**TS**	spur-shaped lobe of A+?SA complex
**SA (SA1, SA2, SA3)**	subaxial sclerites (subaxial sclerite 1, subaxial sclerite 2, subaxial sclerite 3).
**SL**	superior left lobe of lamella copulatrix
**SLb**	superior lobe
**SR**	superior right lobe of lamella copulatrix
**SRP**	superior right peripheral sclerite
**S**	suture
**Sw**	swell
**TFP**	trochantofemoral pit
**Tub**	tubercle
**VPCAM**	ventral process of clypeal anterior margin
**VsSt**	ventral surface of abdominal sternite
**WScP**	weakly sclerotized part

### Taxon selection

The entire data matrix comprises 110 taxa, 4 of which comprise the outgroup. Outgroup taxa belong to the subfamilies Aphodiinae: tribe Aphodiini (*Aphodius erraticus* and *Podotenus fulviventris*) and tribe Aulonocnemini (*Manjarivolo sp*. and *Aulonocnemis crassecostata africana*) (S1 Table). Aphodiinae are conventionally considered the sister group to Scarabaeinae. This fact gains support from various molecular and morphological phylogenies [31–33]. They share 44 synapomorphies in morphology-based phylogeny of the family Scarabaeidae [34]. However, since Aphodiinae still lack any thorough phylogenetic analysis, there is no robust evidence that could support monophyly of this group. Given the high morphological heterogeneity of taxa within Aphodiinae and its non-phylogeny based systematics, there is a high probability that Aphodiinae might be paraphyletic (see also Philips [31]). Therefore, for the purpose of the current analysis we selected representatives of Aphodiinae *sensu stricto* (i.e., tribe Aphodiini) and *sensu lato* (i.e., tribe Aulonocnemini) to account for phylogenetic uncertainty. We specifically selected the tribe Aulonocnemini, which is considered a subfamily by some authors, since the species of this tribe share a number of characteristics common for Scarabaeinae and usually absent in other Aphodiinae. These characteristics are: metatibia with one apical spur, anterior portion of hypomeron depressed, protibia truncated apically. The taxonomically different outgroup taxa were included for their potentially informative input in resolving basal relationships in Scarabaeinae.

The ingroup taxa were sampled to cover taxonomic, biogeographic, and morphological diversity. They belong to all 12 tribes and to 101 genera of the subfamily which represents 37% of the total generic diversity (Scarabaeinae currently totals ca. 258–267 genera according to information compiled from Scholtz et al. [4] and FG personal database). The sampling of taxa across taxonomic categories was biased on purpose. According to the available phylogenies the monophyly of the majority of the tribes is almost entirely undoubted, except for two tribes Deltochilini (formerly Canthonini) and Dichotomiini which are always shown to be polyphyletic (see review of phylogenies section). Together, these two tribes comprise 146 genera and are the most genera-rich in the subfamily. Therefore, in the present study, we selected a few representatives from the tribes with supported monophyly and concentrated greatest focus on the tribes Deltochilini and Dichotomiini covering almost all putative phylogenetic lineages. In this paper the tribal classification for genera follows the most recent list of genera provided by Scholtz et al. [4] with some taxonomic modification introduced by Vaz-de-Mello [35].

### Material deposition

The deposition of material for each taxon used in the analyses is summarized in S1 Table. The codons used in the table are as follows:

- *CASC* California Academy of Sciences, San Francisco (N. Penny, D. Kavanaugh)
- *CMNC* Canadian Museum of Nature, Ottawa (F. Génier)
- *CNCI* Canadian National Collection of Insects, Arachnids and Nematodes, Ottawa (V. Grebennikov and B. Gill)
- *cST* Sergei Tarasov private collection
- *FMNH* Field Museum of Natural History, Chicago (M. Thayer and J. Boone)
- *MNHN* Museum national d’Histoire naturelle, Paris, France (O. Montreuil and A. Mantilleri)
- *TMSA* Ditsong National Museum of Natural History (formerly Transvaal Museum), Pretoria (R. Müller)
- *UPSA* University of Pretoria, Insect collection (C. Deschodt and C. Scholtz)
- *ZMUC* Natural History Museum of Denmark (A. Solodovnikov)

### Examination of specimens and morphology

Specimens were either dry-pinned or alcohol-preserved. At least one specimen per species was entirely dissected. The number of males and females dissected per species is given in S1 Table. In addition to the dissected specimens, on average, we examined 1–3 dry-pinned specimens per species to study external morphology and dissect aedeagi.

The dissection process involved the following phases. Main body parts (usually head, prothorax, pterothorax, legs, wings, elytra, abdomen, genitalia) of alcohol-preserved specimens were separated using forceps/scalpel and placed in 10% KOH for several hours or overnight; wings were directly placed in distilled water. Dry specimens were softened in warm distilled water prior to separation of their body parts. After KOH treatment, the specimens were rinsed in distilled water; aedeagus and spiculum gastrale were placed in glycerin for study and permanent storage; internal sac was separated from the aedeagus. The remaining tissue on the body parts was removed by washing under a fine stream of water. Dark and strongly pigmented body parts, in which structural details were difficult to observe under light microscope, were bleached with 3% hydrogen peroxide containing one drop of ammonium hydroxide. The time required for bleaching ranged from few minutes to few hours depending on the specimen size and desired result. After bleaching, the body parts were rinsed in distilled water, and along with the remaining dissected elements including wings, placed in absolute ethanol for a few minutes; after which they were preserved in glycerin for study and permanent storage. We used tissue culture plates to store dissected specimens. Each plate consists of six compartments that allow separate storage keeping specific body part(s) separately, thus avoiding intermixing of body elements. This procedure provides easy and fast access to a structure of interest during the course of comparative study.

### Morphological principles and terminology

Edmonds [36] provides the most comprehensive review of Scarabaeinae morphology that has been published so far. Although, his work deals with the comparative morphology of Phanaeini species only, the close similarity of morphological structure means this terminology can be applied to all Scarabaeinae. The vast majority of these terms are conventionally accepted by scarabaeine experts. Although, some terms, mainly body sclerites, are inconsistent with those used in describing general beetle morphology (e.g., Lawrence et al. [33]), here we do not attempt integration of these two nomenclatures, and therefore largely follow conventional terms from Edmonds’ work. Terminology of wing venation follows [37, 38] and John Lawrence’s personal notes.

Nomenclature of male genitalia including the endophallic sclerites follows that of Tarasov and Solodovnikov [13] with changes introduced due to reassessment of some previous homologies, which was a result of examination of a significantly larger taxon sample in the current study. The main change concerns the complex of SA and A sclerites in the tribes Coprini and Onitini. Criterion of position and bigger taxon sample suggest that SA sclerites in Coprini and Onitini are homologous to the A sclerite in Onthophagini and Oniticellini, while A sclerite in Coprini and Onitini is homologous to the complex of SA sclerites in Onthophagini and Oniticellini.

### Character selection and coding

Phylogenetically informative characters, including autapomorphies that can be informative in model-based analyses, were sampled from external and internal skeletal structures in the adult body as well as from male genitalia including the internal sac. The character states used in previous morphological phylogenies [11, 17, 20] were investigated; however, their interpretation was problematic due to the rich taxonomic sample of dung beetles which necessitated *de novo* character sample and homology assessment.

Some variable interspecific characters used in the taxonomy of Scarabaeinae were not scored due to their continuous nature which posed problems for their delimitation in discreet units across the large taxa set. These characters are as follows: head shape, size and shape of eye, leg length, body shape, cervical sclerites, metendosternite shape, anterior margin and shape of protibia.

Standard criteria of homology [39–41] were applied to assess the hypotheses of primary homology. These hypotheses were coded in the data matrix mainly using absent/present (a/p) coding scheme. The a/p coding scheme was used in the majority of cases to score absence or presence of morphological structures. This scheme was chosen due to the following reasons: (1) it provides a straightforward means to formalize morphological traits into a data matrix by only answering a question whether the feature is present or absent; (2) it provides an accurate way to separate trait by only identifying one subset of taxa sharing a common feature (character state: present) in an entire set of taxa; (3) this approach was shown to be efficient for coding characters of endophallic sclerites and phylogenetic inference in onthophagine dung beetles [13]. A detailed discussion of the arguments in favor of the a/p coding scheme is provided in [13].

### Character matrix

The character matrix was constructed using Mesquite ver. 2.75 [42]. We do not provide a table version of the data matrix in this paper since available computer programs and web-based applications offer much handier visualization opportunities. The present character matrix can be downloaded as supplementary material (S1 Matrix) or can be viewed online and downloaded from MorphoBank (http://www.morphobank.org project 1157).

The present character matrix comprises 110 taxa and 205 characters; all characters consist of 2 states, except characters #181, 182 which have 3 states; 29 characters are parsimony uninformative but were scored as they may be informative for taxonomy as well as for the Bayesian analysis that takes them into phylogeny estimation.

### Anatomy ontology construction

The anatomical ontology is presented as a tree-graph to reflect the hierarchical relations among anatomical elements and was used to guide partitioning of the character matrix in Bayesian analyses. The ontology was built solely to meet the purpose of the current study characterizing the topological (positional) interrelations among morphological structures (characters). These relations were presented as a tree-like graph where terminal branches correspond to the characters in the character matrix, and nodes represent anatomical regions where the characters or the other nodes (anatomical regions) of a lower hierarchical level are located. The content of this anatomical ontology is quite reductive in contrast to the ontologies aiming at comprehensive characterization of the anatomy (e.g., Yoder et al. [43], Donitz et al. [44]) but might be further elaborated to fulfill the needs of a comprehensive ontology.

The ontology used in this paper initially corresponded to a database constructed in Microsoft Access. The structure of the database comprises two columns: the first column contains character names from the data matrix and names of morphological regions, while the second column contains names of the region to which the entity of the first column is related. In ontological terms, the *part_of* type relation relates the first column to the second one. Extraction of an entire chain of relationships for a specific character from this database results in a string of relations. To exemplify, the character [shape of axial sclerite] is *part_of* [axial sclerite] that is *part_of* [endophallic sac] that is *part_of* [aedeagus] that is *part_of* [abdomen] that, in turn, is *part_of* [body]. Since more than one terminal (character) can be related to a node (region) that, in turn, can be related to another node (region) of higher hierarchical level, the relationships represent a tree-like graph. The entities reflecting dung beetle anatomy are based on the terminology developed by Edmonds [36]. To construct a tree-like graph of the anatomical relations, we exported the database to R [45] and made a script (available upon request) that translated database relations into a graph using the APE package [46].

The tree-like graph of ontology was used to generate various partitioning schemes in the Bayesian analyses assuming that morphological structures located on the same anatomical region have similar evolutionary rates (see also Bayesian analyses section).

### Parsimony analyses

The parsimony analyses were conducted in TNT ver. 1.1 [47]. The continuous nature of the characters #6, 121, 122 challenges their partitioning into characters with a finite number of states. Since application of morphometric methods which can objectively score continuous structures is beyond the scope of the present paper, we ran an initial series of analyses to account for ambiguity in the coding of such characters. This series comprises four analyses each with the data matrix composed of modified set of characters to be excluded from the analysis: analysis #1 (no characters excluded), analysis #2 (characters # 6 excluded), analysis #3 (characters #121, 122 excluded), and analysis #4 (character # 6, 121, 122 excluded). The parsimony analyses in the first series were conducted under equal weights using the following TNT options: tree buffer set to store 10^6 trees, TBR, trees were automatically condensed after search. Each of those four analyses comprises two successive searches with branch swapping set to 1000 and 3000 replications respectively, up to 200 trees saved per replication and random seed randomly generated. The successive searches were used to check the convergence of the analyses in finding the most parsimonious trees (MPTs).

The results of those four analyses gave evidence that the dataset contains conflicting characters resulting in instability of some taxa and hence many shortest trees, which, in turn, yielded a poorly resolved consensus (see results section for details). To identify those taxa, we used a protocol detecting unstable taxa and characters supporting that instability [48]. This protocol uses positional congruence (reduced) index (PCR) to run on the data matrix with parsimonious trees in order to determine and then exclude unstable taxa/clades. This procedure is repeated over the course of successive iterations until all unstable taxa/clades are eliminated. A list of characters supporting instability for every unstable taxon/clade is provided throughout the course of the analysis. Using TNT script given in the original paper [48], we applied this protocol to the data matrix comprising all characters (none excluded) and trees obtained in the first series of the analyses with 1000 replications (total 11137 trees). The 1000 replications analysis was preferred over the 3000 replications analysis as both share similar topological composition but the former contains fewer trees, thus saving computational time. The file produced by the script with the list of characters supporting instability per taxon/clade was evaluated to estimate the frequency of occurrence of every single character supporting instability across the entire set of unstable taxa (not clades). The frequency of occurrence is summarized in the supplementary material (S2 Table).

In the next series of two analyses (analyses #5 and #6 respectively) we excluded characters most frequently supporting instability, namely the data matrix in analyses 5 excluded characters #122, 71, 73, 74, 161, 204, whereas the matrix in analyses #6 excluded characters #6, 48, 57, 128, 159, 168, 175. Both analyses ran under equal and implied weighted parsimony. The equal weight searches used the same options as those in the analyses #1–4 but the number of TBR replications in two searches were 1000 and 5000. The implied weight searches also used the same parsimony options but the number of replications was reduced to 1000. We used eleven different concavity factor values (k_n_ = 1, 10, 20,… (10n-10),… 90, 100) to explore the sensitivity of topology under varying weighting conditions.

Bremer support [49], hereafter BSV (Bremer support values), was used to assign support values onto branches of the consensus trees in the equal weight analyses. Bremer support was calculated by searching suboptimal trees up to 10 steps longer than the shortest one using TBR swapping on the shortest trees.

The synapomorphies were mapped in WinClada [50] onto the most parsimonious tree using an option showing unambiguous changes only.

### Bayesian analyses

The entire analytical procedure for phylogenetic inference and different parameter–partition schemes comparisons using Bayesian framework included the following steps: we first ran 164 Bayesian analyses (82 [parameter—partition models] * 2 [datasets with and without autapomorphies]) to infer phylogenies and assess convergence. Next, we selected only those analyses that had completed and converged, estimated their harmonic mean, and ran stepping stone sampling on those datasets (Table 3).

**Table 3:** Description of parameter-partition schemes tested using Bayesian framework.

PS	NoP	#ID	Among-partition linkage of branch length	Among-partition linkage of rate multiplier	Among-character rate variation	Number of parameters
1	11	1.2	Unlinked	NA	Equal	11
1	11	1.4	Unlinked	NA	Shared gamma	12
1	11	1.5	Linked	NA	Per partition gamma	12
1	11	1.6	Unlinked	NA	Per partition gamma	22
1	11	1.7	Linked	Unlinked	Equal	12
1	11	1.8	Unlinked	Unlinked	Equal	22
1	11	1.9	Linked	Unlinked	Shared gamma	13
1	11	1.10	Unlinked	Unlinked	Shared gamma	23
1	11	1.11	Linked	Unlinked	Per partition gamma	23
1	11	1.12	Unlinked	Unlinked	Per partition gamma	33
2	8	2.2	Unlinked	NA	Equal	8
2	8	2.4	Unlinked	NA	Shared gamma	9
2	8	2.5	Linked	NA	Per partition gamma	9
2	8	2.6	Unlinked	NA	Per partition gamma	16
2	8	2.7	Linked	Unlinked	Equal	9
2	8	2.8	Unlinked	Unlinked	Equal	16
2	8	2.9	Linked	Unlinked	Shared gamma	10
2	8	2.10	Unlinked	Unlinked	Shared gamma	17
2	8	2.11	Linked	Unlinked	Per partition gamma	17
2	8	2.12	Unlinked	Unlinked	Per partition gamma	24
3	6	3.2	Unlinked	NA	Equal	6
3	6	3.4	Unlinked	NA	Shared gamma	7
3	6	3.5	Linked	NA	Per partition gamma	7
3	6	3.6	Unlinked	NA	Per partition gamma	12
3	6	3.7	Linked	Unlinked	Equal	7
3	6	3.8	Unlinked	Unlinked	Equal	12
3	6	3.9	Linked	Unlinked	Shared gamma	8
3	6	3.10	Unlinked	Unlinked	Shared gamma	13
3	6	3.11	Linked	Unlinked	Per partition gamma	13
3	6	3.12	Unlinked	Unlinked	Per partition gamma	18
4	6	4.2	Unlinked	NA	Equal	6
4	6	4.4	Unlinked	NA	Shared gamma	7
4	6	4.5	Linked	NA	Per partition gamma	7
4	6	4.6	Unlinked	NA	Per partition gamma	12
4	6	4.7	Linked	Unlinked	Equal	7
4	6	4.8	Unlinked	Unlinked	Equal	12
4	6	4.9	Linked	Unlinked	Shared gamma	8
4	6	4.10	Unlinked	Unlinked	Shared gamma	13
4	6	4.11	Linked	Unlinked	Per partition gamma	13
4	6	4.12	Unlinked	Unlinked	Per partition gamma	18
5	7	5.2	Unlinked	NA	Equal	7
5	7	5.4	Unlinked	NA	Shared gamma	8
5	7	5.5	Linked	NA	Per partition gamma	8
5	7	5.6	Unlinked	NA	Per partition gamma	14
5	7	5.7	Linked	Unlinked	Equal	8
5	7	5.8	Unlinked	Unlinked	Equal	14
5	7	5.9	Linked	Unlinked	Shared gamma	9
5	7	5.10	Unlinked	Unlinked	Shared gamma	15
5	7	5.11	Linked	Unlinked	Per partition gamma	15
5	7	5.12	Unlinked	Unlinked	Per partition gamma	21
6	6	6.2	Unlinked	NA	Equal	6
6	6	6.4	Unlinked	NA	Shared gamma	7
6	6	6.5	Linked	NA	Per partition gamma	7
6	6	6.6	Unlinked	NA	Per partition gamma	12
6	6	6.7	Linked	Unlinked	Equal	7
6	6	6.8	Unlinked	Unlinked	Equal	12
6	6	6.9	Linked	Unlinked	Shared gamma	8
6	6	6.10	Unlinked	Unlinked	Shared gamma	13
6	6	6.11	Linked	Unlinked	Per partition gamma	13
6	6	6.12	Unlinked	Unlinked	Per partition gamma	18
7	4	7.2	Unlinked	NA	Equal	4
7	4	7.4	Unlinked	NA	Shared gamma	5
7	4	7.5	Linked	NA	Per partition gamma	5
7	4	7.6	Unlinked	NA	Per partition gamma	8
7	4	7.7	Linked	Unlinked	Equal	5
7	4	7.8	Unlinked	Unlinked	Equal	8
7	4	7.9	Linked	Unlinked	Shared gamma	6
7	4	7.10	Unlinked	Unlinked	Shared gamma	9
7	4	7.11	Linked	Unlinked	Per partition gamma	9
7	4	7.12	Unlinked	Unlinked	Per partition gamma	12
8	5	8.2	Unlinked	NA	Equal	5
8	5	8.4	Unlinked	NA	Shared gamma	6
8	5	8.5	Linked	NA	Per partition gamma	6
8	5	8.6	Unlinked	NA	Per partition gamma	10
8	5	8.7	Linked	Unlinked	Equal	6
8	5	8.8	Unlinked	Unlinked	Equal	10
8	5	8.9	Linked	Unlinked	Shared gamma	7
8	5	8.10	Unlinked	Unlinked	Shared gamma	11
8	5	8.11	Linked	Unlinked	Per partition gamma	11
8	5	8.12	Unlinked	Unlinked	Per partition gamma	15
9	1	9.1	Linked	NA	Equal	1
9	1	9.3	Linked	NA	Shared gamma	2

Abbreviations: PS, partitioning scheme (see Table 5); NoP—number of partitions in dataset; #ID, id of parameter-partition scheme, every scheme was analyzed twice with dataset containing all characters and with a dataset excluding autapomorphic characters. In the "Among-partition linkage of rate multiplier" NA indicates that the rate multiplier parameter is not used in estimation. See also S3 Table showing model likelihood and Bayes factor for the listed schemes.

#### Bayesian phylogenetic analyses

In all Bayesian analyses, we excluded characters #122, 71, 73, 74, 161, 204 that were found to be strongly related to taxon instability in parsimony analyses. Bayesian analyses were performed using MrBayes v. 3.2.1 [51] on the Abel Cluster, at the University of Oslo. The dataset was analyzed under *Mk* model for morphology [52], with equal state frequencies, and the ascertainment bias set to variable as only variable characters were scored in the matrix. All analyses ran 30 M generations, two simultaneous runs and four chains, with sampling parameters and trees every 10^4 generations. The temperature parameter for heating the chains was set to 0.2. Convergence was assessed by the average SD of split frequencies and, in some selected analyses, also by visual examination of trace plots using Tracer [53] for likelihood and parameters. The analyses were judged converged when the average SD of split frequencies reached value 0.01 or lower. If the values were higher than 0.01, the analysis was reported as not converged (S3 Table). If an analysis was still running after 30 days of computation and the convergence was not achieved, the analysis was stopped. This was done as we aimed at finding a computationally efficient partitioning scheme and therefore considered 30 days as a limit beyond which the scheme is no longer efficient computationally given the relatively small size of our morphological dataset. Stopped analyses are marked as demanding long computational time (S3 Table). The burn-in was set to 25% of samples as this value was always enough to discard the initial high-failure portion.

#### Models: parameter-partition schemes

We analyzed and tested 82 models that differ in the partitioning of the dataset and parameter linkage amongst the dataset parts. The ontology tree-like graph was used to generate various partitioning schemes in BI. This approach is based on the assumption that morphological structures located on the same anatomical region share similar evolutionary dynamics. This is a simplified model given that real evolution of characters is more complex and the tree graph does not always depict complex relations among anatomical structures. Nevertheless, such a model can represent a reasonable approximation of the real-life biological phenomena. A previous study [16] using a partitioned dataset in a Bayesian analysis of morphology corroborates this simplification.

**Fig 3:**
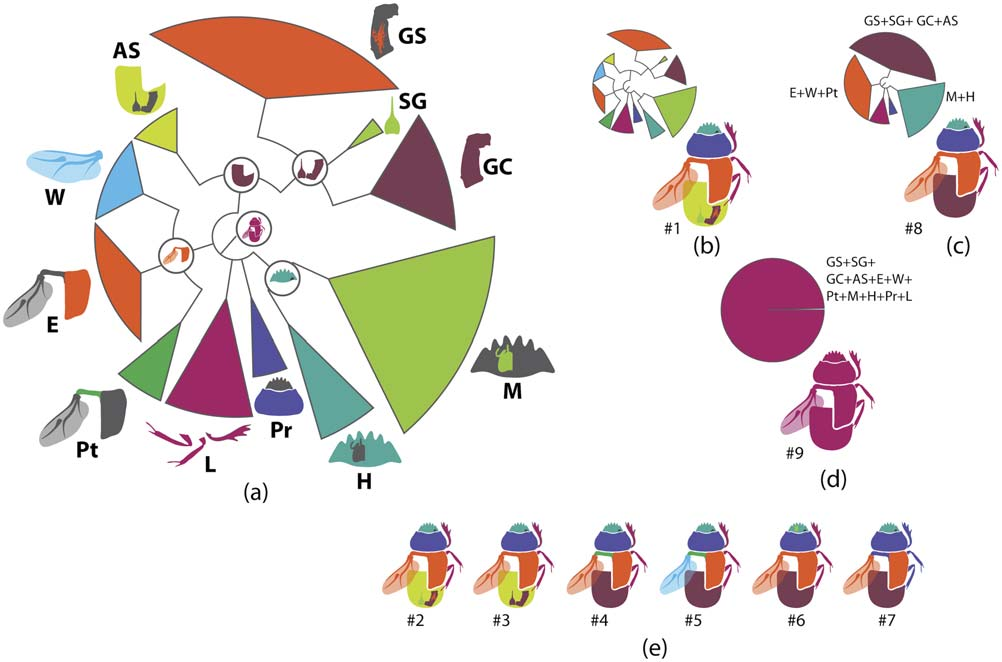
Ontology of Scarabaeinae and partitioning schemes used in Bayesian analyses. a, Graph of Scarabaeinae ontology reflecting anatomical characters relationship from datamatrix. This graph guided generation of partitioning schemes in Bayesian analyses. The tips of the graph refer to elementary categories of characters and their relative size correspond to the number of characters in the category. The abbreviations and uniquely colored pictures of beetle anatomical part associated with each tip specify the elementary category (see Table 4.) b-d, exemplified partitioning schemes: the uniquely colored tips of the graph above beetle picture characterize partitions used in Bayesian analyses; abbreviations (see Table 4) associated with tips specify the elementary categories included in the partition; the beetle body parts are colored in accordance with the partition they belong to. The number # indicates the ID of partitioning scheme from Table 5. e, The remainder partitioning schemes demonstrated without graphs. Beetle body parts are colored in accordance with the partition they belong to. The number # indicates the ID of partitioning scheme from Table 5.

In the present study, the graph of ontology (Fig. 3a) consists of eleven elementary categories of characters grouped according to their anatomical position (Table 4). The relationships between the elementary categories reflect their anatomical relationships. Navigating from the apical part of the graph toward the basal part, the elementary categories can be merged into larger categories. This approach is used here to generate various partitioning schemes. In the simplest case, each elementary category can become a separate partition resulting in a dataset consisting of 11 partitions (Fig. 3b), more partitioning schemes can be generated by merging two or more elementary categories. To exemplify, the graph includes 3 elementary categories which comprise characters of *wings (W)*, *elytra (E)* and *pterothorax (Pt)*. The characters of all 3 categories are parts of the pterothorax, thus these 3 categories can be joined in one supercategory called *“pterothorax”*. Likewise, the elementary category *mouthparts (M)* can be merged with the category of characters of the head (H) into one supercategory *“head”* as the characters of both are located on the same anatomical region—the head. The supercategories *“pterothorax”* and *“head”* along with the rest elementary categories can be used to form a separate partitioning scheme consisting of 8 partitions (Table 5). Further merging of categories can for instance generate a scheme with 5 partitions (Fig. 3c), and walking down to the root of the graph culminates in merging all categories and gives a dataset with 1 partition (Fig. 3d). All possible and meaningful combinations of the elementary categories and the supercategories of different hierarchical level for the current dataset result in nine different partitioning schemes (Fig. 3b-e, Table 5).

**Table 4:** Elementary categories and associated characters.

Abbreviation	Name of category	Characters placed in category
**E**	Elytra	65, 66, 67, 68, 69, 70, 71, 72, 73, 74, 75, 76, 77, 78, 79, 80, 81, 82, 83, 84, 85, 86, 87, 88, 89, 90, 91, 92, 93, 94, 95
**W**	Wings	96, 97, 98, 99, 100, 101, 102, 103, 104, 105, 106, 107, 108, 109, 110, 111, 112, 113, 114, 115, 116
**GC**	Genital capsule	1, 2, 3, 4, 5, 6, 7, 8, 9, 10, 11, 12, 13, 14, 15, 16, 17, 18, 19
**ES**	Endophallic sclerites	20, 21, 22, 23, 24, 25, 26, 27, 28, 29, 30, 31, 32, 33, 34, 35, 36, 37, 38, 39, 40, 41, 42, 43, 44, 45, 46, 47, 48, 49, 50, 51, 52, 57, 58, 59, 60, 61, 62, 63, 64
**SG**	Spiculum gastrale	53, 54, 55, 56
**H**	Head	147, 148, 149, 150, 151, 152, 153, 154, 155, 156
**M**	Mouthparts	117, 118, 119, 120, 121, 122, 123, 124, 125, 126, 127, 128, 129, 130, 131, 132, 133, 134, 135, 136, 137, 138, 139, 140, 141, 142, 143, 144, 145, 146
**L**	Legs	167, 168, 169, 170, 171, 172, 173, 174, 175, 176, 177, 178, 179, 180, 181, 182
**Pr**	Prothorax	157, 158, 159, 160, 161, 162, 163, 164, 165, 166
**Pt**	Pterothorax	183, 184, 185, 186, 187, 188, 189, 190, 191, 192
**AS**	Abdominal sternites	193, 194, 195, 196, 197, 198, 199, 200, 201, 202, 203, 204, 205

The column “name of category” corresponds to elementary categories (partitions) from Fig. 3. “Characters placed in category” lists characters from datamatrix belonging to a specific category.

**Table 5:** Partitioning schemes.

#	Partition scheme	Number of Partitions
**1**	GS, SG, GC, AS, E, W, Pt, M, H, Pr, L	11
**2**	GS, SG, GC, AS, E+ W+ Pt, M+ H, Pr, L	8
**3**	GS+SG+ GC, AS, E+ W+ Pt, M+ H, Pr, L	6
**4**	GS+SG+ GC+AS, E+ W, Pt, M+ H, Pr, L	6
**5**	GS+SG+ GC+AS, E, W, Pt, M+ H, Pr, L	7
**6**	GS+SG+ GC+AS, E+W+Pt, M, H, Pr, L	6
**7**	GS+SG+ GC+AS, E+W, M+ H, Pt+Pr+ L	4
**8**	GS+SG+ GC+AS, E+W+Pt, M+H, Pr, L	5
**9**	GS+SG+ GC+AS+E+W+Pt+M+H+Pr+L	1

# indicates ID of a partitioning scheme; the column “partition scheme” lists elementary categories (partitions) in the partition scheme. The sign + indicates that two or more elementary categories are merged in one partition. For the expansion of abbreviations and further explanation, see Fig. 3 and Table 4.

Different schemes of parameter linking among partitions can be applied to accommodate heterogeneity of evolutionary rates among characters. The heterogeneity can be implemented by modeling among-partition rate variation, and among-character rate variation within a partition. Linking/unlinking of among-partition and among-character rates among parts of the data matrix sets different constraints on the rates of characters evolution. In MrBayes among-partition rate variation is controlled by two parameters: branch length and rate multiplier. Linked branch length assumes the same relative branch length for the partitions, while unlinked branch length (MrBayes option: *unlink brlens*) allows relative branch length to vary among partitions. The rate multiplier controls the relative average substitution rate. It can be fixed (MrBayes command *prset ratepr = fixed*) assuming the same substitution rate for each partition. Alternatively, the rate multiplier can be set to variable (command *prset ratepr = variable*) allowing substitution rate to vary under the constraint that the average rate among partitions totals one. The among-character rate variation can be implemented using a gamma model (Г) assuming that among-character rate within a partition follows gamma distribution. The gamma model is controlled by one shape parameter defining shape of the gamma distribution. MrBayes implements options allowing use of equal rates without application of a gamma model (*lset rates = equal);* use of shared gamma model where shape parameter is linked among partition (*lset rates = gamm*a); and use of a per partition gamma model providing separate estimation of shape parameter per every single partition (*lset rates = gamma unlink shape = ()*).

The branch length priors are known to sometimes affect the results of analyses [54]. However, here we do not perform a test of branch length priors, and use the default *exp(10)*, as the branch length priors were shown not to significantly affect estimation of model likelihood in the morphological dataset [16]. All possible combinations of linking/unlinking aforementioned parameters were used to construct different parameterization schemes which in couple with a set of partitioning schemes yielded 82 different parameter—partition models with the number of parameters varying from 1 to 33 (Table 3).

We also investigated the effect of exclusion/inclusion of autapomorphies (29 characters), on the topology. Unlike parsimony that treats autapomorphies as being uninformative, Bayesian framework uses them in inference; however, the effect of inclusion of autapomorphic characters in morphology-based Bayesian analysis has not been broadly studied. To investigate exclusion versus inclusion, we tested each of the 82 parameter–partition schemes by analyzing the dataset containing all characters and the dataset excluding autapomorphies.

Bayes factor was used to choose among parameter—partition models. The interpretation of Bayes factor for model choice follows Kass and Raftery [55]. The marginal likelihood for calculation of Bayes factor was estimated using two methods: (1) stepping-stone sampling [56] and (2) harmonic mean [57, 58]. The stepping-stone method is considerably more accurate than the less reliable harmonic mean method [56]. We used both approaches for two reasons: (1) for comparing both methods and (2) for substituting the stepping-stone by the harmonic mean approach if the former could not reach completion within a reasonable time limit. The analyses demanding long computation time were interrupted after 30 days and marginal likelihood was calculated using harmonic mean only.

The stepping-stone sampling was run in MrBayes with the same option as phylogenetic analyses, but for 10 M generations using 3 runs, sampling every 10^4 generations, with number of steps set to 50, alpha parameter 0.4, burninss-1, and the first 25% samples of each step were discarded as burn-in. The harmonic mean of likelihood was calculated from the output of MCMC run through *sump* command in MrBayes.

## Results

### Parsimony analyses

**Fig 4:**
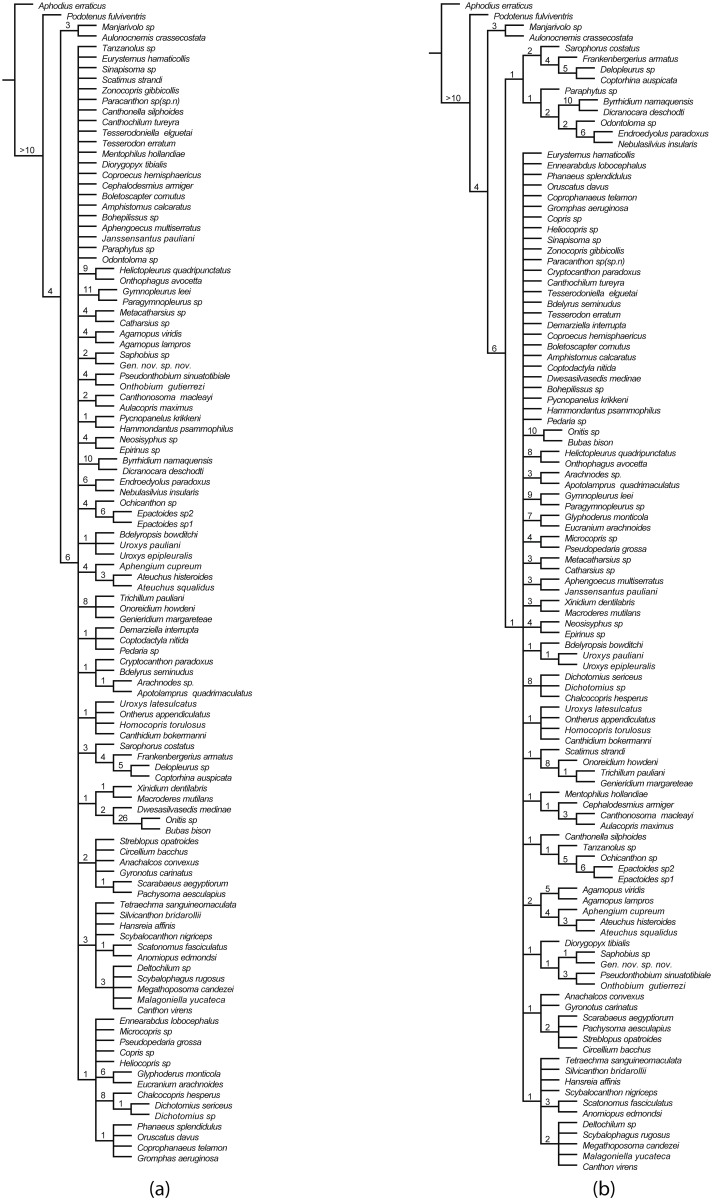
Strict consensus of parsimonious trees of Scarabaeinae, with Bremer support values. a, Strict consensus tree with Bremer support values from the analysis #1; unweighted parsimony, dataset with all characters. b, Strict consensus tree with Bremer support values from the analysis #5; unweighted parsimony, dataset excluding characters #122, 71, 73, 74, 161, 204.

**Fig 5:**
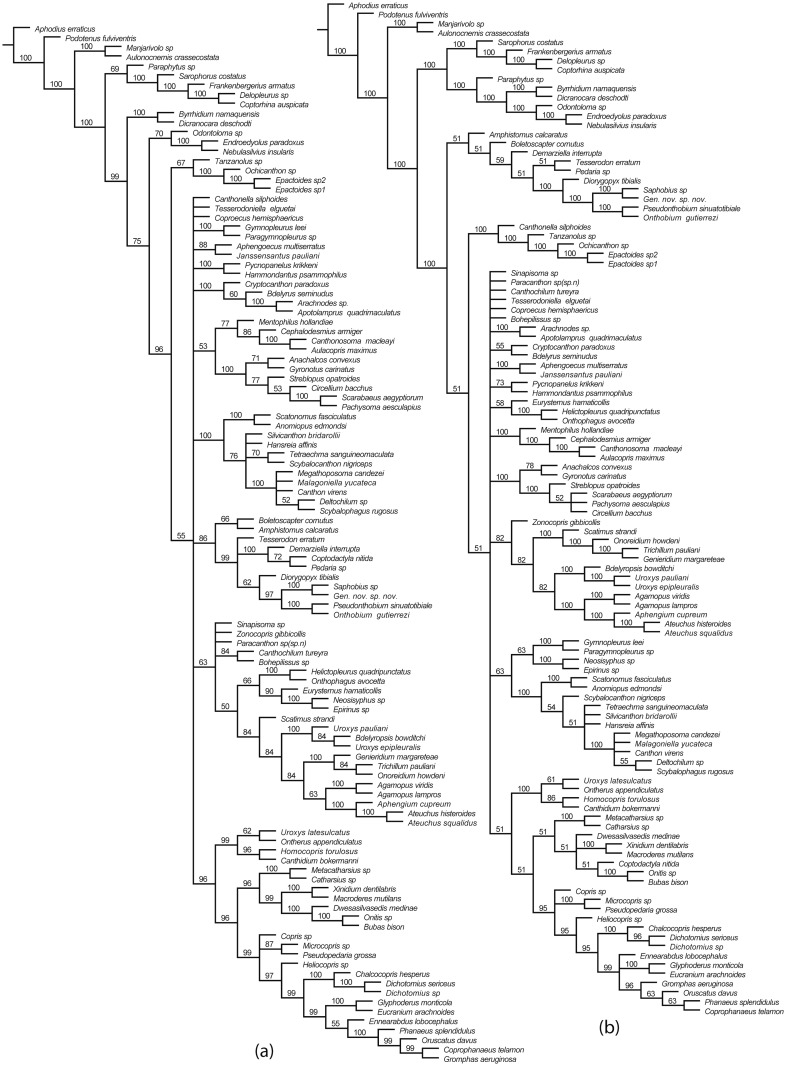
Majority consensus of parsimonious trees of Scarabaeinae. a, Majority consensus (50%) of MPTs from the analysis #1; unweighted parsimony, dataset with all characters. b, Majority consensus (50%) of MPTs from the analysis #5; unweighted parsimony, dataset excluding characters #122, 71, 73, 74, 161, 204.

The first series of four analyses yielded the results shown in Table 6. The topological composition of trees between searches with 1000 and 3000 replications in the analyses #1, 3, 4 was similar given the close frequency values in the majority rule (50%) consensus. Both searches differ only in the number of trees found which was higher in the 3000 replications searches. The analysis # 2 with 1000 did not find the most parsimonious trees as its trees were one-step longer than those of the respective analysis with 3000 replications. The strict consensus of analyses #1 with the dataset containing all characters is poorly resolved (Fig. 4), its majority rule consensus is shown in Fig. 5a. The strict consensus of analyses #2, 3, 4 where from 1 to 3 characters were excluded, are similar to that of analysis #1 in the resolution and topology but their majority rule consensus results differ from that of analysis #1 as well as from each other (S1–S4 Figs.). This differences are due to the distinction in topological composition among the analyses indicating that their topologies are quite sensitive to the character exclusion. This likely points to the fact that the data matrix includes conflicting characters causing instability for some taxa and thus changing topological composition of parsimonious trees.

**Table 6:** Summary of statistics for parsimony phylogenetic analyses.

# Analysis	Character composition	RI	CI	L	N of trees
**1**	all characters	2.99	0.33	529	25723 (3000 repl.)
**2**	characters # 6 excluded	2.91	0.34	512	392 (3000 repl.)
**3**	characters #121, 122 excluded	2.84	0.35	497	11686 (3000 repl.)
**4**	characters # 6, 121, 122 excluded	2.76	0.36	481	11491 (3000 repl.)
**5**	characters #122, 71, 73, 74, 161, 204	2.57	0.39	439	23681 (5000 repl.)
**6**	characters #122, 71, 73, 74, 161, 204, 6, 48, 57, 128, 159, 168, 175 excluded	2.30	0.44	377	164436 (5000 repl., analyses stopped after 4813 repl. as tree buffer was full)

The column “N of trees” shows number of MPTs obtained in the searches with max number of replications.

Running the method of Pol and Escapa [48] on the present dataset required 8 iterations before stability was reached and all unstable taxa/clades eliminated. The resulting stabilized topology comprised only 27 taxa out of the 110 initially included. Such a phylogenetic tree was uninformative for the format of the present study that aims at phylogenetic reconstruction for all taxa included. In addition to the instable taxa, 38 characters were associated with instability in those taxa. The list of clades/taxa and characters supporting their instability is provided in S1 Document. The summary of the frequency at which a character was found to support instability (S2 Table) revealed that the majority of those characters support instability for more than one taxon. Interestingly, characters #122 and 6 excluded *a priori* in the first series of analyses were identified supporting instability 6 and 3 times respectively, which confirms their ambiguity.

With the second series of analyses (analyses #5, 6) we tested the effect of exclusion of these characters, and following the aim of the paper we kept all taxa included. Analysis #5 excluded those characters found to be most frequently associated with instability (6 and 4 times respectively), while analysis #6 excluded those characters deleted in analyses #5, in addition to the characters which caused instability 3 times (S2 Table and material and methods section).

**Fig 6:**
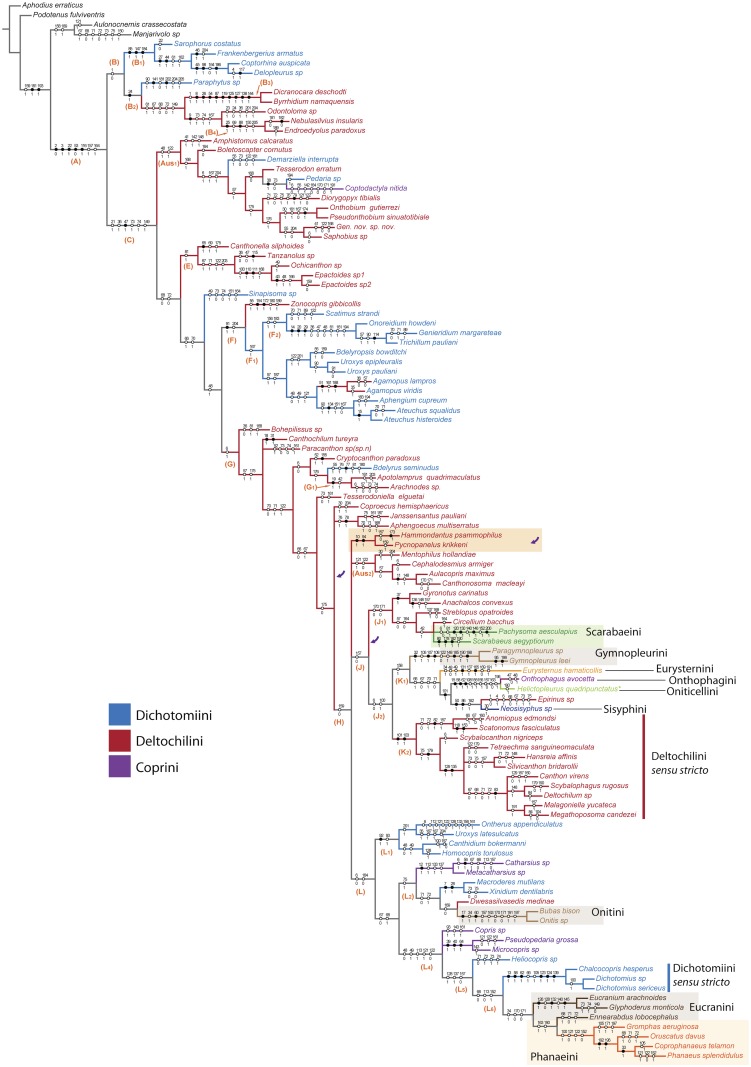
Phylogenetic tree of Scarabaeinae from parsimony analysis #5 (implied weight parsimony, dataset excluding characters #122, 71, 73, 74, 161, 204), with mapped synapomorphies. This topology was obtained with concavity factor ranging from 40 to 100. In addition to this tree, this range of factors also yielded two other MPTs. Those two parsimonious trees differs only in the position of the clade (*Hammondantus psammophilus* + *Pycnopanelus krikkeni*) which is highlighted in orange and its alternative positions are arrowed. Branches of the tree are colored according to the Scarabaeinae taxonomy.

**Fig 7:**
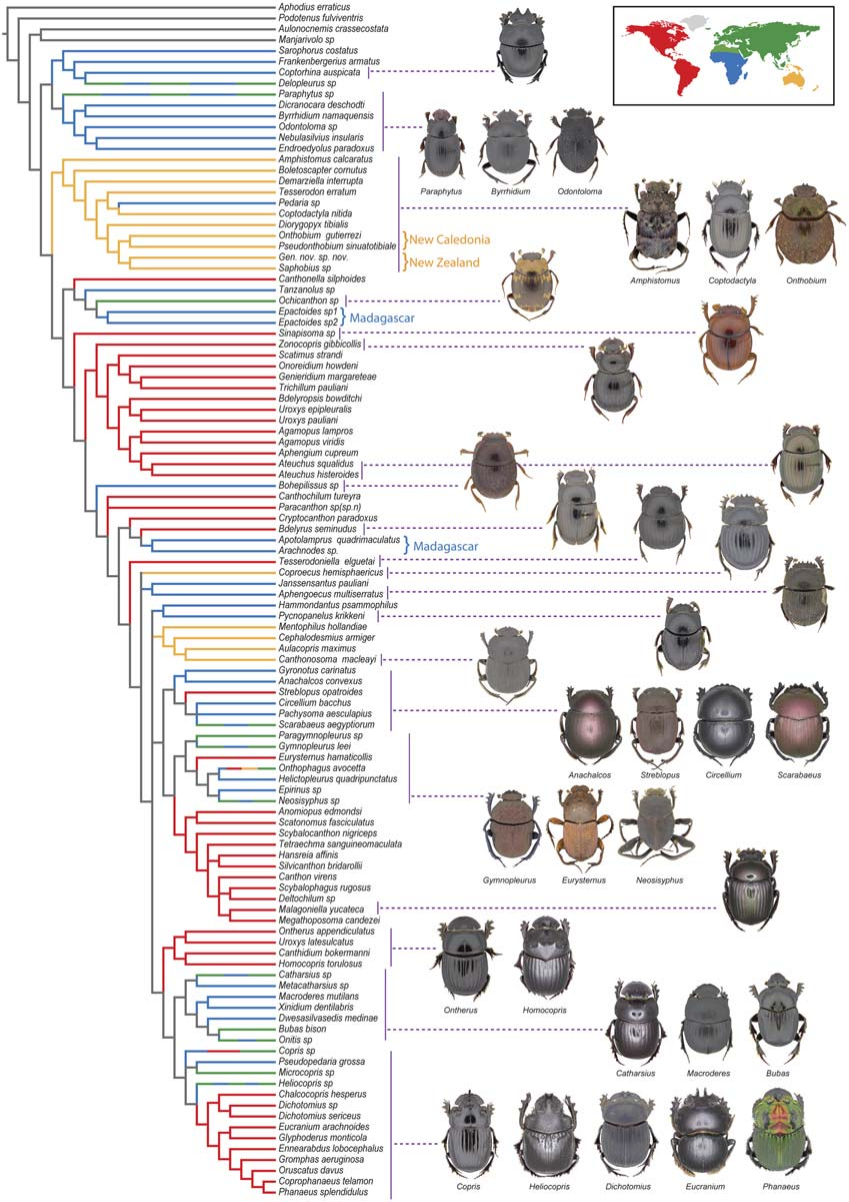
Phylogenetic tree of Scarabaeinae from parsimony analysis #5 with implied weights (same tree as in Fig. 6, see that figure for description). Branches of the tree are colored according to the area of endemism of the genus. The scheme of biogeographic regions is provided on the top of the figure (note: the Palearctic and Oriental Regions are combined for clarity). The photographs of beetles correspond to the taxa used in the analyses.

The statistics of trees for analyses #5 and 6 is provided in Table 6. The majority rule and strict consensus of analysis #5 is shown in Figs. 5b, 4b. The majority consensus is similar to that of analysis # 6 (S4 Fig.) but differs by the position and support of some clades. Under implied weights analysis #5 yielded trees of the same length as the unweighted parsimony analysis when concavity factor was ranging from 30 to 100. Noteworthy, implied weight analyses with k = 40–100 always uncovered the same set of the three most parsimonious trees (Fig. 6, 7). The analysis #6 with implied weights also yielded parsimonious trees of the same length as unweighted parsimony analysis when the concavity factor was varying from 20 to 100. The number of inferred trees in this range of concavity factor varied from 50 to 200.

### Bayesian analyses: model comparison and inference

**Fig 8:**
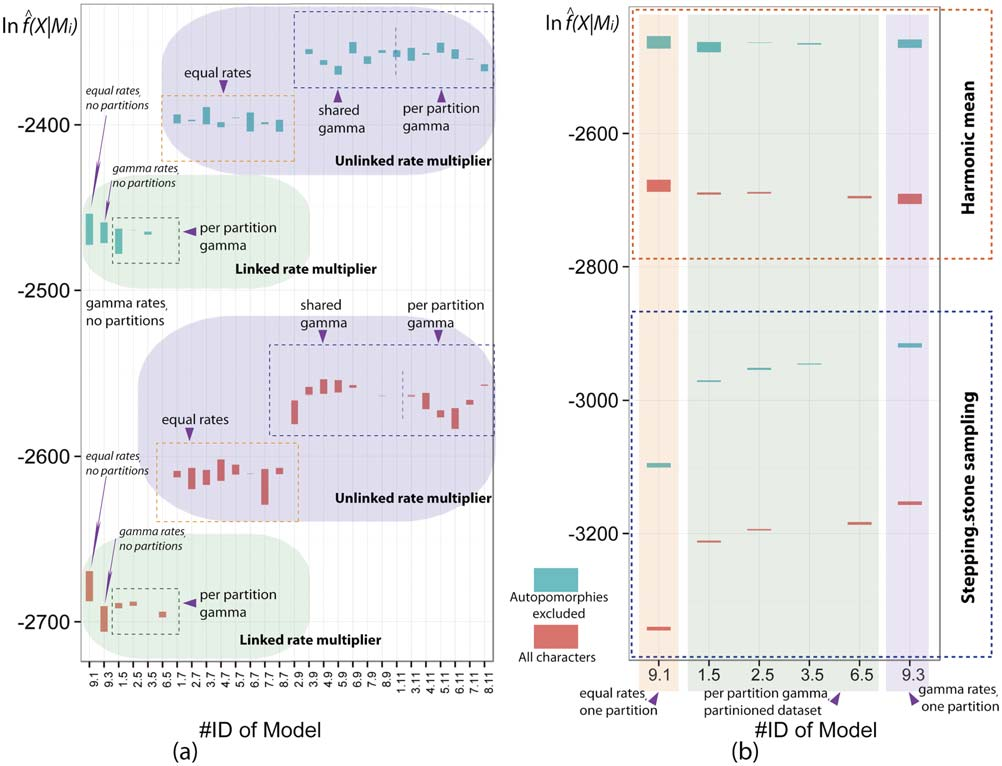
Comparison of model likelihoods. #ID of model correspond to the specific parameter-partition scheme described in Table 3 and S3 Table. The red and blue columns, on the plot, corresponding to dataset with and without autapomorphies respectively, show variation of likelihood values (max and min) from different runs within specific parameter-partition scheme. a, comparison of model likelihoods estimated using harmonic mean method. b, comparison of model likelihoods estimated using stepping stone and harmonic mean methods.

We calculated model likelihood for both the harmonic mean and the stepping-stone methods only for analyses that converged in the MCMC run (Fig. 8a, b, S3 Table). Many analyses, including all of those with unlinked branch length, did not converge, likely due to the presence of superfluous parameters in the character deficient partitions whose size does not allow efficient parameter estimation. In addition, all stepping-stone analyses with unlinked rate multiplier were extremely time consuming and were thus interrupted due to the reasons described in the material and methods section (S3 Table).

The differences between harmonic mean and stepping stone estimators for the same analysis were in the range ca. 400–800 likelihood units with harmonic mean being constantly higher. Absolute variation in estimated model likelihood among different runs within the same analysis was low in the stepping-stone method (range of likelihood units 1.18–6.72; Bayes factor values *2lnBF*: 2.36–13.44) and much higher in the harmonic mean method (range of likelihood units 0.07–21.52; Bayes factor values *2lnBF*: 0.14–43.05), (Fig. 8a, b). Model likelihood values positively correlate between analyses with and without autapomorphies being significantly higher in those without them. However, likelihood values cannot be directly compared between these two types of analyses as these analyses use two different datasets (with and without autapomorphies).

In the harmonic mean method, significantly diffused likelihood values between runs in the same analysis overlap across the different analyses, allowing reliable comparison only across the clusters of the analyses separated by large gaps in the likelihood space (Fig. 8a). Because of that, we can compare only between 3 clusters of analyses: (1) with linked rate multiplier, (2) unlinked rate multiplier and equal rates, (3) unlinked rate multiplier, shared and per partition gamma. Given across-analyses overlap within the clusters, the differences among models within them must be taken with caution due to likelihood variation. Bayesian comparison prefers cluster of models with the specific quality of the parameters, namely unlinked rate multiplier and variation among characters modeled with gamma distribution (regardless if shared or per partition), while partitioning scheme and overall number of the parameters did not matter. The model with the highest average marginal likelihood among analyses with all characters is #8.11 (Bayes factor *2lnBF* of this model and the others in the cluster ranges from 1.4 to 39.7), while in the analyses without autapomorphies is #6.9 (*2lnBF* of this model and others ranges from 0 to 27.2). The Bayes factors are summarized in S3 Table.

Due to the computational time constraints, stepping-stone analyses were completed only for the parameter schemes with linked rate multiplier. In these analyses, the absolute variation of likelihood values among runs within analyses was significantly lower than differences of the values amongst the analyses allowing unambiguous comparison of all analyzed parameter-partition schemes. Interestingly, in the analysis with linked rate multiplier, a comparison using the harmonic mean is uninformative due to the overlapping variation, while the stepping-stone method generates less-dispersed estimates (Fig. 8b). The Bayes comparison with the stepping-stone prefers two-parameter model #9.3 (one partition and gamma rates) in both analyses with and without autapomorphies. This model has significantly higher likelihood in comparison to the remaining models with more parameters. In the analyses with all character Bayes factor (*2lnBF*) of #9.3 and its closest model #6.5 (7 parameters) is 29.7, while in the analyses without autapomorphies *2lnBF* of #9.3 and the closest model #3.5 (7 parameters) is 56.7. Interestingly, model #9.1 (one partition and equal rates) has the lowest marginal likelihood, while model#9.3 with similar parameterization scheme but gamma rates has the highest likelihood.

**Fig 9:**
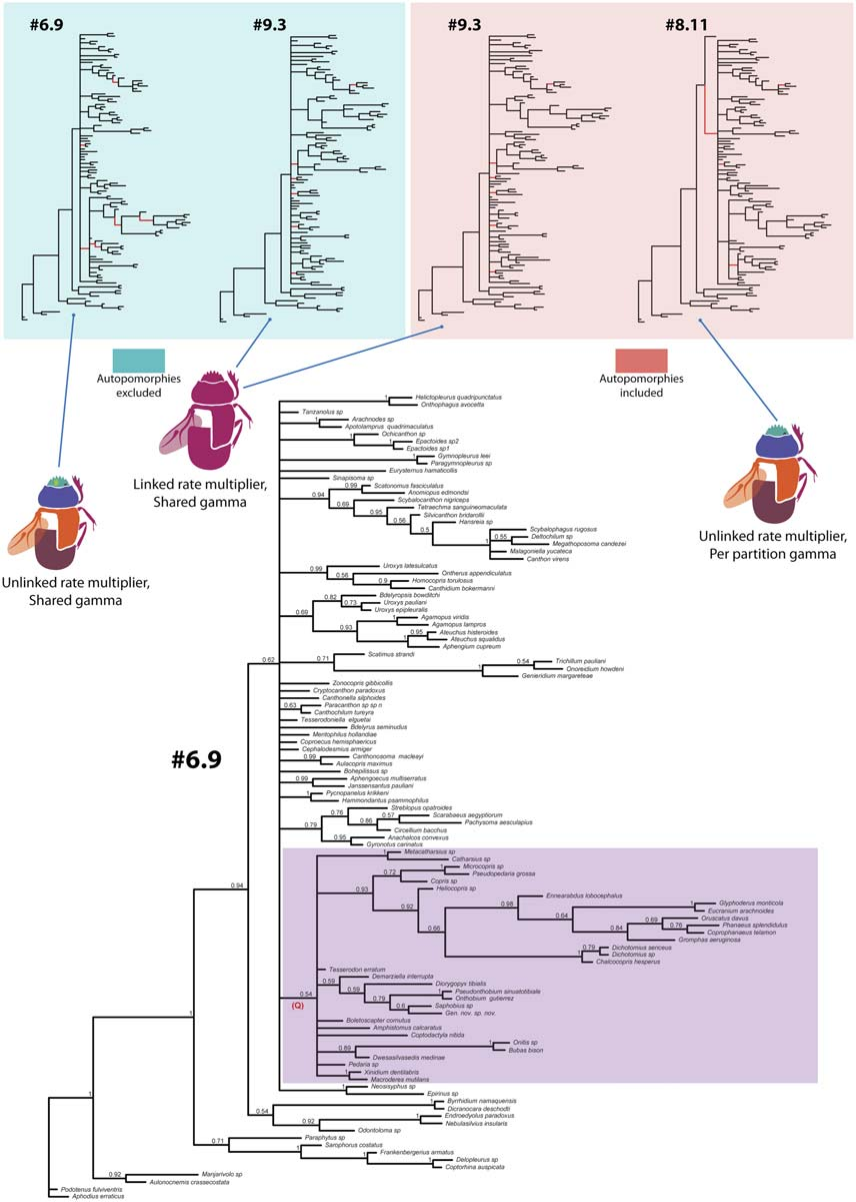
Summary of trees from Bayesian analyses. Top figures, 4 phylogenetic trees with removed tip labels from the Bayesian analyses corresponding to the best partition-parameter model (complete tree can be found in S5–S7 Figs.). The branches colored in red on trees #9.3 and #8.11 show clades unique to those trees in comparison to the tree corresponding to the model #6.9 (without autapomorphic characters) that has highest model likelihood among all the analyses. The branches colored in red on #6.9 show unique clades in comparison to #9.3 (without autapomorphic characters). Model #6.9 and #8.11 have the highest average model likelihood in the analyses with unlinked rate multiplier, while model #9.3 is the best in analyses with linked rate multiplier. The trees corresponding to the models are placed in color boxes indicating inclusion or exclusion autapomorphies. Beetle figures and associated descriptions under the tree illustrate parameter-partition schemes used in the analyses. The large central tree was obtained from the scheme, with the highest model likelihood, #6.9 (without autapomorphic characters). Clade values correspond to posterior probabilities. The clade Q, which recovered only in Bayesian analyses, is highlighted.

Although, the differences in likelihood scores and Bayes factors varies drastically, the inferred topologies are similar across different models as well as analyses with and without synapomorphies. Additionally, the topologies of Bayesian analyses are congruent with those from parsimony. The trees of the best models and their differences are exemplified in Figs. 9, S5–S7.

Regardless of the parameter-partition schemes, the topologies across the Bayesian analyses, despite some differences, are similar with each other and parsimony, which agrees with the result of previous study testing partitioning of morphology in birds [16]. Additionally, other studies analyzing morphological dataset with parsimony and Bayesian approaches, but without partitioning, also report topological consistency between the two methods [14–16].

### Inferred topologies

All strict consensus trees from parsimony analyses (Fig. 4a, b) are poorly resolved; the resolution of majority rule consensuses is significantly better. The analyses with all characters and with ambiguous characters excluded yielded similar strict and majority rule consensus trees which mainly differ in the composition of basal clade B and presence (Figs. 5, 4) of the clade Aus_1_ uniting the majority of Australasian taxa (Fig. 5).

The trees from Bayesian analyses (Fig. 9) are also poorly resolved. Both Bayesian and parsimony approaches inferred largely, although not entirely, congruent topologies. The principal discrepancy between the parsimony and Bayesian analyses with the best models (#6.9 without autapomorphies, and #8.11 with autapomorphies), is clade Q recovered in Bayesian analyses with posterior probability 0.54 and 1 respectively (Figs 9, S7). Noteworthy, that this clade also disappears in single-partition Bayesian analyses which, in turn, yields trees most similar to parsimony ones.

For the in-depth description of the phylogenetic results, we will specifically focus on the preferred phylogenetic trees obtained from parsimony analysis with implied weights and excluded ambiguous characters (analysis #5) due to reasons mentioned in the results of the parsimony analysis section. Analysis #5 with implied weights yielded 3 MPTs which are largely resolved and differ only in the position of the clade composed of Afrotropical genera *Hammondantus* and *Pycnopanelus* (Figs. 6, 7). The topology of preferred trees is generally congruent with the trees from the other analyses.

The subfamily Scarabaeinae emerged as monophyletic (clade A) in both Bayesian as well as in parsimony analyses.

The clade B, which we here refer as “basal Scarabaeinae” is sister to the remaining Scarabaeinae and comprises largely Afrotropical and two Afro-Oriental genera. The taxa recovered in this clade have a number of plesiomorphic character states in male genitalia (e.g., see characters # 1, 21, 23, 36). “Basal Scarabaeinae” separate into two monophyletic groups. One of them (clade B_1_) includes genera *Sarophorus*, *Frankenbergerius*, *Coptorhina* and *Delopleurus*, while the other (clade B_2_) includes genera *Paraphytus*, *Dicranocara*, *Byrrhidium*, *Odontoloma*, *Nebulasilvius* and *Endroedyolus*.

The sister to “basal Scarabaeinae”, clade C, groups together all the remaining taxa. One of the nodes (Aus_1_) branching off next node C contains majority of genera from Australasian Region included in present paper (10 out of 15 taxa), and one Afrotropical genus *Pedaria* nested within them. This Australasian—Afrotropical clade is absent in the strict consensus tree, but present in the majority rule consensus (support value 51%). The strict consensus does not support nested position of Afrotropical *Pedaria* within this clade, while the subclade comprising New Zealand + New Caledonia taxa holds in the strict consensus (BSV 1).

The clade Aus_2_, incorporates another part of the Australasian taxa that emerge separately from the majority of those in clade Aus_1_.

Monophyly of a remarkable group (clade F), comprising exclusively Neotropical genera, is supported in preferred tree but not consensus. Within this clade the snail-dwelling deltochiline genus *Zonocopris*, comes out as sister to a clade comprising mostly Dichotomiini genera which, in the sense of Vaz-de-Mello [35], are classified in the tribe Ateuchini (clade F_1_).

Monophyletic group formed by Madagascan genera *Apotolamprus* + *Arachnodes* (clade G_1_) comes out as a sister group to Neotropical genus *Bdelyrus*. Combined, these Madagascan taxa and *Bdelyrus* form a sister clade to Neotropical *Cryptocanthon*.

With the highest concentration of tribal diversity, the next major clade J, includes more than a half of the tribes involved in the present study. Within this clade the subclade J_2_ is most taxonomically rich—it includes tribes (semi-)endemic to Old World (Gymnopleurini, Sisyphini), broadly distributed worldwide (Onthophagini, Oniticellini), endemic to the Neotropics (Eurysternini), and deltochiline genus *Epirinus* occurring in southern Africa. High number of autapomorphies defining many branches in this clade points to a dramatic overall morphological distinction among its members. Such a high density of morphologically distinct taxa may evidence in increased rate of morphological evolution within this part of the tree.

Clade K_2_ consisting of Deltochilini *sensu stricto* comprises genera *Deltochilum*, *Canthon* and their allies. We refer to the clade as Deltochilini *sensu stricto* since it contains *Deltochilum* the type genus of the tribe. Noteworthy, the tribe Deltochilini, in its present concept, is spread throughout the entire tree being highly polyphyletic. However, given the present topology, the limits of Deltochilini, have to be confined solely to Deltochilini *sensu stricto* that includes exclusively Neotropical taxa. In addition to the taxa involved in this study, many other genera of Deltochilini from the Neotropics, share common synapomorphies with the Deltochilini *sensu stricto* and thus have to be placed within this group in the future. Noteworthy, during the course of present study, we have not been able to elucidate any genus outside the Neotropics fitting synapomorphies of Deltochilini *sensu stricto*.

The other major monophyletic group (clade L) is composed of the tribes Onitini, part of Dichotomiini, Eucraniini, and Phanaeini. The member of this clade the genus *Uroxys* appears polyphyletic in present phylogeny as its two other species come out within the subtribe Scatimina (clade F_1_).

The Old World tribe Onitini (*Bubas* + *Onitis*) comes up as the sister to the deltochiline southern African genus *Dwesasilvasedis*, they both are nested within the larger clade L_2_ composed of Old World genera *Catharsius*, *Metacatharsius*, *Macroderes* and *Xinidium*.

The tribe Coprini appears polyphyletic and separates into three different lineages; its genus *Coptodactyla* emerges within Australasian clade (Aus_1_); two other lineages are composed of *Catharsius* + *Metacatharsius*, and *Copris*, *Pseudopedaria* and *Microcopris*.

Monophyletic group solely composed of Neotropical representatives (clade L_5_) from the tribes Eucraniini, Phanaeini, and Dichotomiini *sensu stricto* is recovered sister to Afro-Oriental genus *Heliocopris*.

The clade Dichotomiini *sensu stricto* includes *Dichotomius* the type genus of the tribe. Overall, the tribe Dichotomiini is recovered to be extremely polyphyletic emerging multiple times in the phylogenetic tree. Apparently, such pattern provides strong evidence that taxonomic limits of Dichotomiini have to be restricted to only incorporate *Dichotomius*, and closely related genera (e.g., *Chalcocopris*).

The tribe Phanaeini emerged monophyletic. Its close relative the tribe Eucraniini occurring in arid regions of South America is recovered paraphyletic; its two genera *Eucranium* + *Glyphoderus* form a monophyletic group, while its genus *Ennearabdus* comes up as sister to the tribe Phanaeini.

## Discussion

### Present phylogeny and the previous studies

Our preferred phylogenetic tree (see results: Inferred topologies section) is consistent with biogeography as monophyletic groups of different hierarchical levels are endemic to specific biogeographic regions (Fig. 7). Present phylogeny, as well as all others, strongly supports polyphyly of Deltochilini and Dichotomiini. The other questionable tribe Coprini, as in previous studies, comes out polyphyletic—it is split into three lineages *Coptodactyla*, *Metacatharsius + Catharsius*, and a polytomy formed by *Copris* and allied (clade L_3_). This pattern, despite some differences, is consistent with previous phylogenies. Specifically, Australasian genus *Coptodactyla*, as in molecular phylogeny [25], comes up within the Australasian clade (Aus_1_). Two other lineages *Metacatharsius + Catharsius* and *Copris* + allied, which together from a monophyletic group in [11, 20], emerge as closely affiliated, though not sister to each other. Since testing, the monophyly for the remaining 9 tribes was not the scope of present study and we cannot discuss it due to insufficient taxon sample, we will focus on discussing relationships among them.

The present phylogeny supports sister relationship between the lineage Sisyphini + *Epirinus* and Onthophagini + Oniticellini. These relationships are new and, so far, have not been recovered by any previous phylogeny, although close affiliation of the two lineages is supported by [20, 25] (Fig. 2), where they lie closely to each other on the tree. Interestingly, many morphological and molecular phylogenies support close relationship between tribe Onitini and clade formed by Onthophagini and Oniticellini. This relationship varies depending on phylogeny, either Onitini is sisters to Onthophagini—Oniticellini, or Onitini is nested within Onthophagini—Oniticellini, or something else (Fig. 2). Our phylogeny contradicts with previous patterns and recovers Onitini as only a remote relative of Onthophagini and Oniticellini but sister to the clade (*Dwesasilvasedis + (Macroderes + Xinidium))*. This result is somewhat surprising, and it might be an artifact of our analysis; however the tribe Onitini despite similar body shape to Onthophagini and Oniticellini, share no synapomorphies with them.

The well supported monophyly (BSV 4) of the deltochiline genus *Epirinus* and the tribe Sisyphini was quite unexpected—none of previous phylogenies support it, and usually place these two taxa remotely from each other [20] (Fig. 2). The most similar pattern was recovered by molecular phylogeny [25], in which Sisyphini branches off next to *Epirinus*, but they do not form a monophyletic group. This similarity between two studies is intriguing, likely indicating that both groups are indeed closely related.

The sister relationships of Eurysternini and clade formed by (Onthophagini + Oniticellini) + (*Epirinus* + Sisyphini) has not been previously supported, however this pattern is somewhat similar to the one recovered in morphological phylogeny [20] that returns Sisyphini as sister to polytomy composed of Onthophagini, Oniticellini, Eurysternini and Onitini (Fig. 2). None of the other existing phylogenies place Eurysternini near either Onthophagini + Oniticellini or *Epirinus* + Sisyphini.

The phylogenetic position of tribe Gymnopleurini has been unstable (Fig. 2). The present study recovers it as sister to a clade formed by Eurysternini, Onthophagini, Oniticellini, *Epirinus* and Sisyphini, which in turn comes out as sister to Deltochilini *sensu stricto* (clades K_1_ + K_2_). Although, each existing phylogeny returns different relationships for these groups, molecular phylogeny [24] recovers clade composed of taxa partially belonging to K_1_ (Onthophagini, Oniticellini, Onitini) also, as a sister to Deltochilini *sensu stricto*. The clade Deltochilini *sensu stricto* comprising representatives of the true deltochilines is monophyletic in the present study as well as in molecular phylogenies [24, 25].

Two molecular phylogenies, [24, 25] and present study, are congruent in supporting phylogenetic pattern consisting exclusively of Neotropical taxa (Phanaeini, Eucraniini) + *Dichotomius* + allied. Present phylogeny differs from two molecular phylogenies only in the position of the Eucraniini genus *Ennearabdus* that emerges as sister to the tribe Phanaeini making Eucraniini paraphyletic (Fig. 6), while molecular phylogenies support monophyly of Eucraniini and sister relationship between Eucraniini and Phanaeini. In addition, molecular phylogeny [25] supports sister relationship between *Dichotomius* (Dichotomiini *sensu stricto*) and *Canthidium* which is inconsistent with the present tree where *Canthidium* emerges as sister to *Homocopris* (clade L_1_).

Our study supports the position of *Circellium* within the tribe Scarabaeini (unresolved polytomy) and, in this respect, agrees with other relevant phylogenies [11, 20, 25].

Our phylogeny recovered many congruent clades with two molecular phylogenies [28, 29] which are quite similar in taxon sample but differ in composition of molecular markers. Present tree (Fig. 6) and phylogeny [28] recover monophyly of the clade comprising dichotomiine and deltochiline taxa *Macroderes*, *Xinidium* and *Dwesasilvasedis*; the differences between both phylogenies lie in monophyly for the subclade *Xinidium* + *Dwesasilvasedis* recovered in [28], whereas the present study places these two genera in different though sister subclades *Macroderes + Xinidium* and *Dwesasilvasedis* + Onitini. Monophyly of the clade *Hammondantus + Pycnopanelus* changing its phylogenetic position among three preferred MPTs (Fig. 6) was supported by [28], and not supported by other phylogeny using less number of markers [29]. Yet, monophyly of *Gyronotus + Anachalcos* inferred in the current study was supported by both molecular phylogenies [28, 29].

Position and composition of “basal Scarabaeinae” (clade B) on the current tree is consistent with that of three molecular [25, 28, 29] and one morphological [11] phylogenies. Especially high congruence is shared with phylogenies Sole and Scholtz [28] and Vaz-de-Mello [11]. Our tree and [28] support monophyly of clades herein indicated as B1, B3, B4, however the relationships between these clades differ; additionally, the present tree recovers *Odontoloma* as sister to *Nebulasilvius + Endroedyolus*, while phylogeny [28] places *Odontoloma* as sister to the group consisting of the same genera as those in clade B_1_. The present study and morphological phylogeny [11], although agreeing on the general branching pattern of “basal Scarabaeinae”, slightly differ in the position of “basal Scarabaeinae” on phylogenetic tree, which in phylogeny [11] emerges prebasally.

Present phylogeny as morphological phylogeny [11] supports monophyly for the tribe Ateuchini (clade F_1_) and its subtribe Scatimina (clade F_2_) in the sense of [35]. Amongst all previous phylogenies, only [11] and [25] include sufficient sample of Ateuchini genera for testing its monophyly and intrageneric relationships. The branching pattern within Ateuchini between present and morphological phylogeny [11] is well congruent, albeit some slight differences. On the contrary, in molecular phylogeny [25] Ateuchini genera: *Ateuchus*, *Trichilium*, *Uroxys* and *Bdelyropsis* are dispersed into three separate lineages. Thus, monophyly of Ateuchini and Scatimina is supported by only morphological phylogenies.

Monophyly for the clade Aus_1_, recovered in the present study, and comprising the majority of Australasian genera, one African genus *Pedaria*, and a subclade formed of New Zealand and New Caledonia genera (Figs. 6, 7), is supported by molecular phylogeny [25]. This molecular phylogeny differs from the present tree by supporting different relationships of taxa within the clade Aus_1_ and placing genera *Coproecus* and *Cephalodesmius*, recovered outside Aus_1_, within it. In turn, the present phylogeny places Australasian *Boletoscapter* within Aus_1_, which, in that molecular phylogeny, is a sister to Neotropical *Uroxys*. This congruence between phylogenies is quite intriguing; none of the other phylogenies has ever recovered monophyly for such large set of Australasian taxa with nested African *Pedaria*. The genera in this clade have notably different overall morphology.

The Madagascan deltochilines separate into two lineages, one composed of the genus *Epactoides* is sister to Oriental *Ochicanthon*, which agrees with two molecular phylogenies [27, 29]; the second one consists of the well-supported clade (BSV 3) comprising *Apotolamprus + Arachnodes*. This result disagrees with [27, 29] which recover *Apotolamprus* and *Arachnodes* to be the remote relatives.

### Bayesian analyses: partitioning using anatomy ontology

The Bayesian analysis with ontology-based partitioning of morphological characters incorporates character rate heterogeneity and allows the assessment of evolutionary dynamics of different sets of characters. Thus, based on these results, we can answer the questions raised in the introduction.

*Can the Bayesian approach give new insight into phylogenetic history in comparison to parsimony*? The answer is yes, although the topologies in both methods are generally consistent, they usually differ in the position of some clades/taxa representing new phylogenetic insights. *Is partitioning of the morphological dataset meaningful*? Given that different parameter-partition schemes yield consistent topologies but different likelihoods, this question cannot be answered unequivocally. If one is focused on topology rather than parameter estimation then the use of simple partition-parameter scheme saves computational time and present an optimal way for the topological inference. However, in Bayesian analysis focused on topology inference and parameters, the complex models with unlinked rate multiplier and gamma rates seem to be more appropriate. The complex models were not always preferred using Bayesian comparative framework and often simpler models had significantly higher marginal likelihood (Fig. 8a, b), the phenomenon known as Lindley's Paradox [59]. *Do characters on the same anatomical region evolve at similar rates*? The partitioning of the data based on anatomy generally increases model likelihood, underpinning the fact that structures of the same anatomical region generally share similar evolutionary dynamics. However, the partitioning scheme itself has considerably less effect on the likelihood than linking/unlinking of the parameters used to model rate variation. *How can autapomorphic characters affect tree topology*? The topologies between the analyses with and without autapomorphies are similar. Since both types of datasets tend to provide similar phylogenetic trees, the use of autapomorphies in morphology-based analysis seems unnecessary.

### Biogeography and early evolution of dung beetles

Dung beetle origin is a controversial issue whose main dilemma lies in a Cenozoic versus Mesozoic age of origin [4, 60]. The fossil record for Scarabaeinae is poor; the majority of fossils are Tertiary and only two fossils are known from the Mesozoic. The oldest known fossil *Cretonitis copripes* Nikolajev, 2007 is described from the Lower Cretaceous of Russia (Baysa, Siberia) [61]. The description is based solely on a holotype—the incomplete impression of the middle leg. A lack of any valuable character on the holotype leg does not allow an assessment of its phylogenetic affinities. Thus, we regard the conclusion that this fossil belongs to Scarabaeinae [61] as premature. Owing to the lack of evidence on the taxonomic position of this fossil, we do not consider it a member of Scarabaeinae. Another Mesozoic fossil *Prionocephale deplanate* Lin, 1980 is described from the Upper Cretaceous of China. The original description of that fossil does not list any unambiguous characters for placing it within Scarabaeinae, which indicates that there are no reliable evidence to consider it a member of the subfamily. The latest study [62] dating the phylogenetic tree of the superfamily Scarabaeoidea uses this fossil to calibrate the age of the Scarabaeinae clade. Because affiliation of this fossil with Scarabaeinae is highly doubtful, it cannot be reliably used in the calibration. Therefore, we do not discuss in present paper the ages inferred for the origin of Scarabaeinae in [62].

Besides the abovementioned fossils, there is an ichnofossil from the Nearctic Cretaceous representing a herbivorous dinosaur coprolite and associated dung filled tunnels [63]. However, this ichnofossil lacks any evidence to be attributed as a member of Scarabaeinae as tunnels in dinosaur dung could be formed by other groups of beetles, for example Geotrupidae [4]. So, there are no reliable fossils or fossilized evidence which would support a Mesozoic origin for dung beetles. At the same time, the distribution of some monophyletic lineages of Deltochilini and Dichotomiini fits a Gondwana pattern and may suggest a Mesozoic origin.

The lack of a reliable dated phylogeny does not allow testing a Cenozoic and Mesozoic hypotheses; however, the present tree, if assumed correct, can be used to assess these competing evolutionary scenarios. Here, we discuss the potential implications for this debate, which derived from the biogeographical pattern of the present phylogeny. We consider long-distance dispersal, dispersal via land bridges, and vicariance as main processes that potentially could shape present distribution of Scarabaeinae. Undoubtedly, such approach is speculative as it is not based on a robust biogeographical analyses (e.g., one including ancestral area reconstruction and a dated phylogeny) but currently, due to the lack of other data, it is the only way to get an insight into dung beetle evolutionary history.

First, suppose, dung beetles evolved during the Cenozoic after the Gondwana breakup, then biogeographical composition of such clades as e.g., Aus_1_ (Australasian taxa with nested African genus) and L (consisting of Old World and New World taxa) can only be explained by long-distance intercontinental dispersal events occurring between Africa and Australia as well as between Africa and S. America. We can rule out the dispersal via land bridges scenario as we lack evidence for it. Some members of Scarabaeinae, as Onthophagini, indeed, owe their cosmopolitan distribution to land bridge dispersal from Old World to New World and Australia through Asia as supported by phylogeny indicating that monophyletic Australasian and American clades derive from Asian ancestors ([30] see also[13]). However, Clade L, consisting of Old World and New World taxa, lacks any evidence pointing to the dispersal through Asian—N. American land bridges, as there is no support for sister relationships of Asian taxa with American or Australian ones. Based on the Onthophagini example, we have evidence for dispersal of dung beetles from Asia to Australia, however the remaining endemic Australasian groups have no close relatives in the Oriental fauna and do not show any hint of their common ancestor dispersal from Australia to Asia. Dispersal through Asian land bridge among Old World, New World and Australia could explain biogeographic pattern in Aus_1_ and L only on the assumption of massive extinctions of dung beetle lineages in Asia, for which we have no evidence. Therefore, given these arguments, intercontinental dispersal would seem the most plausible explanation. However, the occurrence of long-distance dispersal seems quite unlikely, as dung beetles have never been shown to have the necessary flight abilities. Almost all volcanic islands (e.g., Canary Islands, Hawaii etc.) which potentially could host Scarabaeinae if they were able to reach them, lack any traces of native dung beetle species. The longest recorded dispersal refers to the presence of two endemic Scarabaeinae genera on volcanic island of Mauritius, which seemingly happened from Madagascar (distance between two is ca. 900 km). This fact suggests that even medium-distance dispersal is a rare event in dung beetles, and assigns a very low probability to intercontinental dispersal requiring a few thousand kilometers to cross.

However, if we assume a Mesozoic origin for the dung beetles it could explain their present biogeographic pattern by Gondwana vicariance. The nested position of African *Pedaria* within the Australasian clade Aus_1_ and biogeographic pattern in L as well as branching of basal lineages (clades B and Aus_1_) would fit well the vicariance hypothesis if they were correspondingly aligned with fragmentation of Gondwana. This alignment would imply minimal age for clades Aus_1_, B and dung beetle origin coinciding with the separation of Africa from Gondwana in Late Jurassic (160 MYA). Clades consisting of New World and Old World taxa (e.g., clade L) would be constrained to minimal age of the separation of Africa from South America, which remained connected until mid-Cretaceous (110–95 MYA). In addition, vicariance pattern can also explain the nested position of the clade composed of New Zealand + New Caledonia taxa within Aus_1_. This phylogenetic pattern is consistent with breakup of a land mass, comprising New Caledonia and New Zealand, from Gondwana at 80 MYA, and its subsequent split separating New Zealand and New Caledonia taxa at 40–30 MYA. Although the current phylogeny supports a vicariant pattern, such a scenario poses quite old minimal ages for the origin of dung beetles and diversification of their main lineages. This strict vicariance can be relaxed assuming that dung beetles originated in Mesozoic after Gondwana breakup, and then dispersed among fragments of Gondwana when they were not too far away from each other. For example, the African south margin broke from Antarctica, the part of Gondwana, at 160 MYA and started drifting slowly northward, remained separated from Antarctica by the Indian opening of 300–1000 km over Early Cretaceous. This assumption can reduce minimal ages for the origin of the dung beetles and their lineages to early—mid Cretaceous and at the same time, it still reserves a key role for Gondwana and its fragments in shaping current distribution of Scarabaeinae.

Although, the conclusion about Mesozoic origin is preliminary, since we lack comprehensive dated phylogeny of dung beetles, the present tree has higher likelihood of fitting this hypothesis. At the same time, we cannot, by far, rule out, the hypotheses of Cenozoic origin.

## Conclusions

Detailed examination of morphology coupled with parsimony and Bayesian analyses using ontology for matrix partitioning has shed new light on the evolution of dung beetles. Many clades in the present phylogeny are congruent with those in other existing molecular and morphological phylogenies while some clades suggest novel relationships that have not been hypothesized before. We can, to a certain extent, consider congruent clades among previous phylogenies as well-supported but the number of such clades is low and does not allow for the selection of any specific phylogeny as more reliable over the others. Our results are consistent with the results from different studies and provide further resolution for Deltochilini and Dichotomiini as well as the remaining Scarabaeinae tribes whose placement has been contentious. Therefore, the present study provides an integrative pattern of phylogenetic relationships in Scarabaeinae, many parts of which are backed up by previous works. The consistency of the current phylogeny with biogeography likely suggests a Mesozoic rather than Cenozoic age of the origin of dung beetles. However, this conclusion is speculative and has to be confirmed by future total evidence studies integrating morphological, molecular and fossil data to date the dung beetle tree of life. The resulting phylogeny demonstrates that the present classification of Scarabaeinae has to be substantially revised. The mapped synapomorphies can be used to define the new (sub)tribes and groups of genera; however, nomenclatural changes, at present, would be premature and more data, primarily molecular, are required to corroborate the findings presented here.

## Character Report

### Illustration notes

The photo-based illustrations of endophallic sclerites in Figs. 10–33 consist of two elements. Those inside black rectangles represent the position of the sclerite complex in an intact position. To take those pictures, we tried to remove the membrane of the sack as much as possible in order to clear visibility for photographing sclerites. Removal of the membrane did not perturb the relative position of the sclerites. The second element of illustration, located around the black rectangles, consists of photos of each separate sclerite, and details the differences in shape. To indicate correspondence of the same sclerites on different photos, we linked them by blue lines.

**Fig 10:**
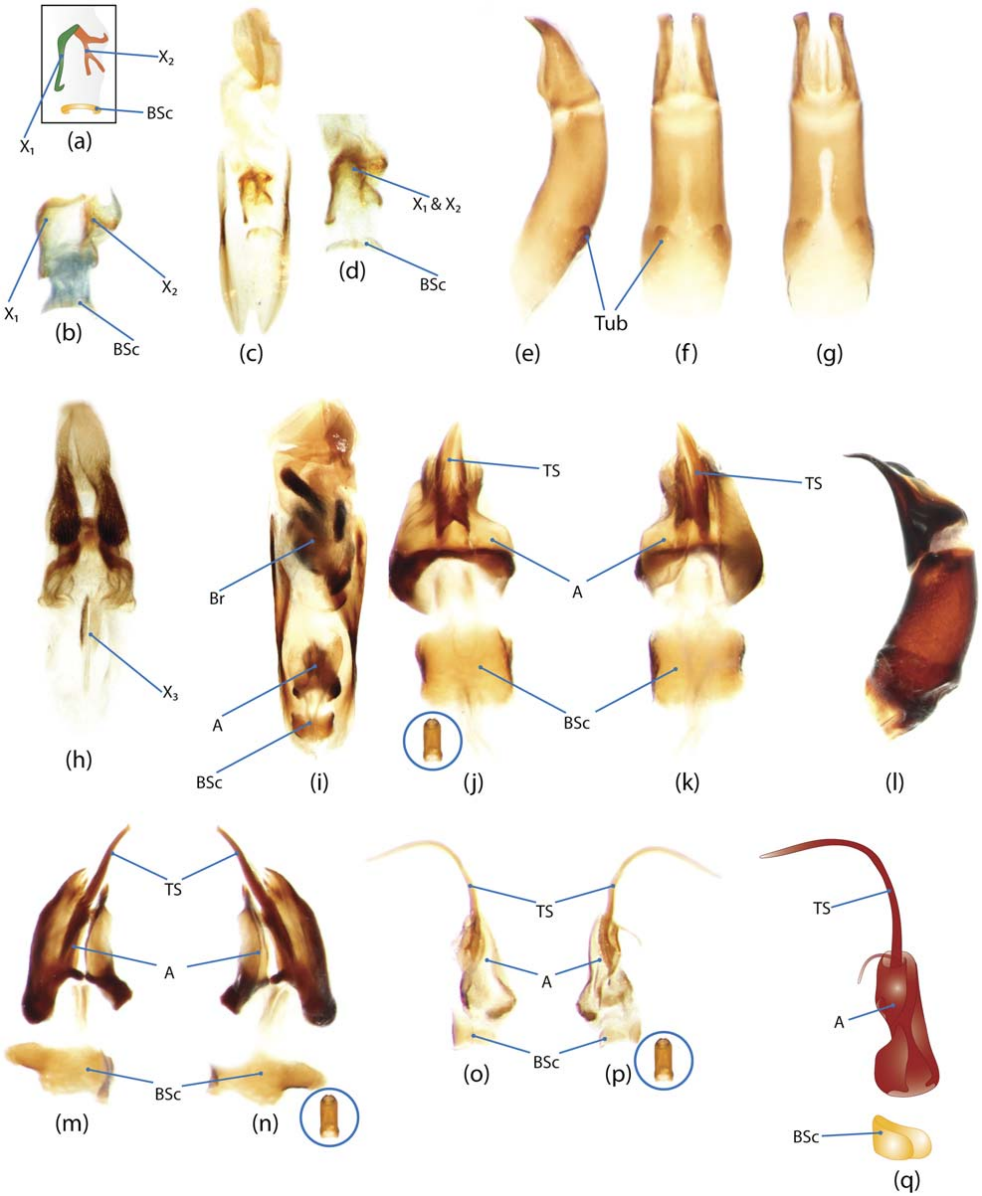
Genitalic elements of Scarabaeinae. a-g, *Odontoloma sp*.; h, *Sarophorus costatus*; i-l, *Byrrhidium namaquensis*; m-n, *Dicranocara deschodti*; o-q, *Nebulasilvius insularis*; a-d, h, i, aedeagal sac with sclerites; e, l, aedeagus, lateral view; f, aedeagus, dorsal view; g, aedeagus, ventral view; j-k, m-n, o-p, aedeagal sclerites; q, scheme of intact aedeagal sclerites.

**Fig 11:**
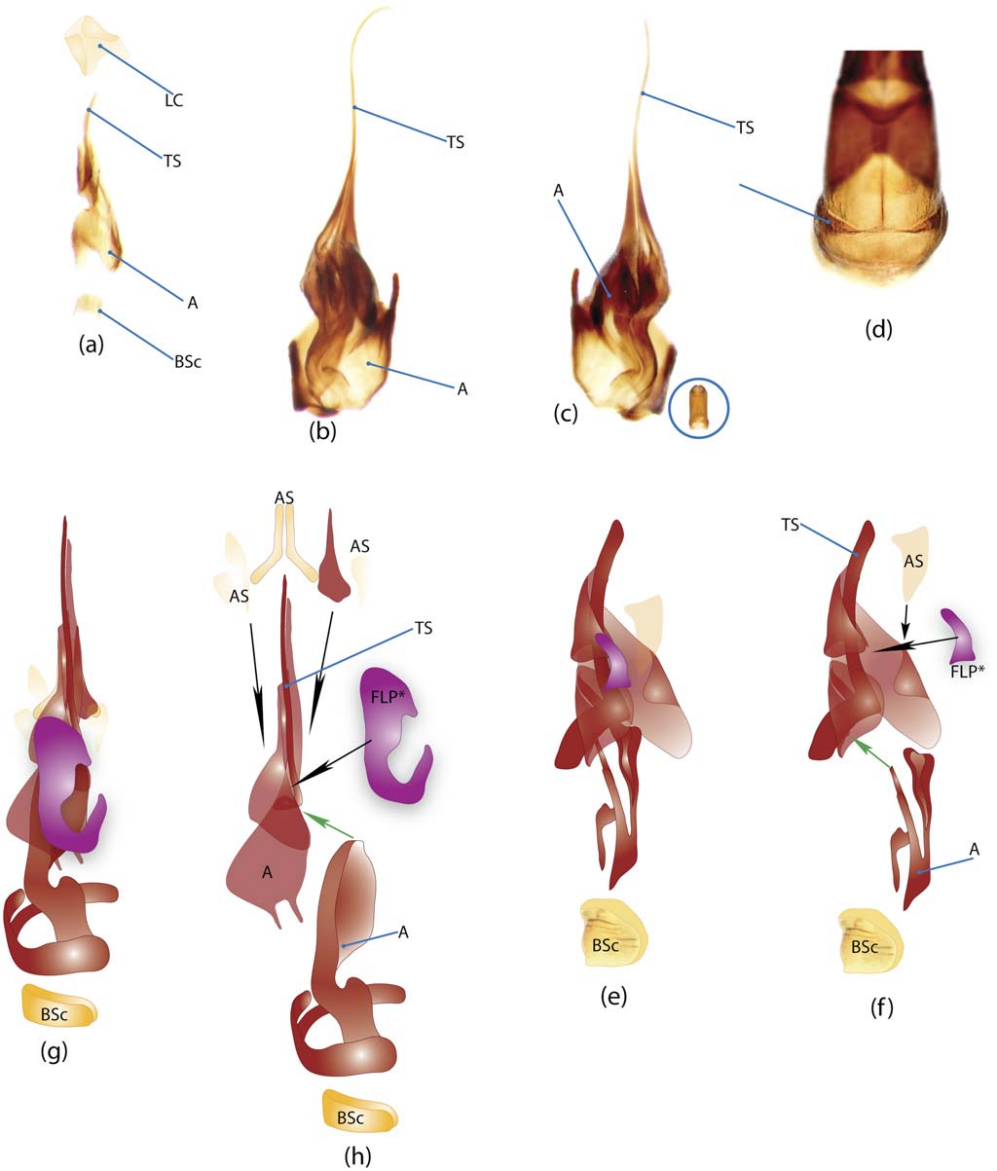
Genitalic elements of Scarabaeinae. b-d, *Paraphytus sp*.; g-h, *Coptorhina auspicata*; e-f, *Frankenbergerius armatus*; a-c, aedeagal sclerites; g, e, scheme of intact aedeagal sclerites; h, f, scheme of dissected aedeagal sclerites; d, phallobase, dorsal view.

**Fig 12:**
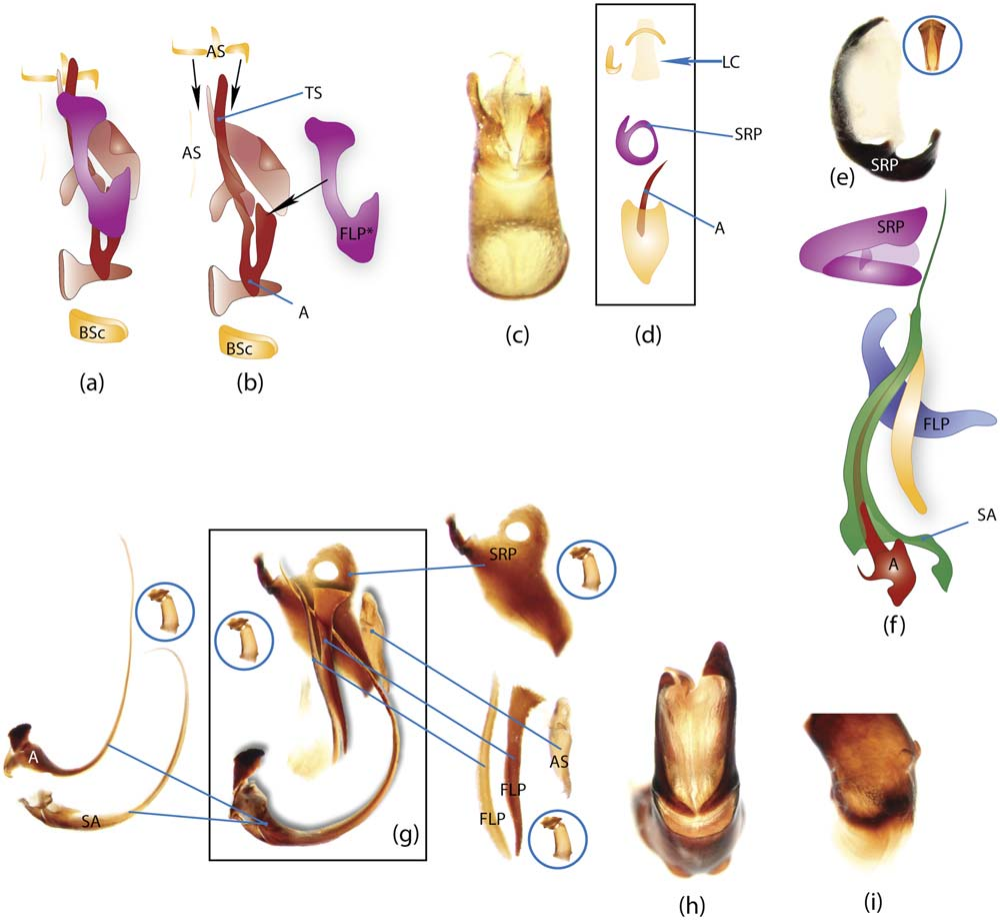
Genitalic elements of Scarabaeinae. a-b, *Delopleurus sp*.; c-d, *Bohepilissus sp*.; e-f, *Anachalcos convexus*; g-i, *Epirinus sp*.; a, d, f, scheme of intact aedeagal sclerites; b, scheme of dissected aedeagal sclerites; e, SRP sclerite; g, i, picture scheme of aedeagal sclerites; h, aedeagus, dorsal view; i, phallobase, left lateral view.

**Fig 13:**
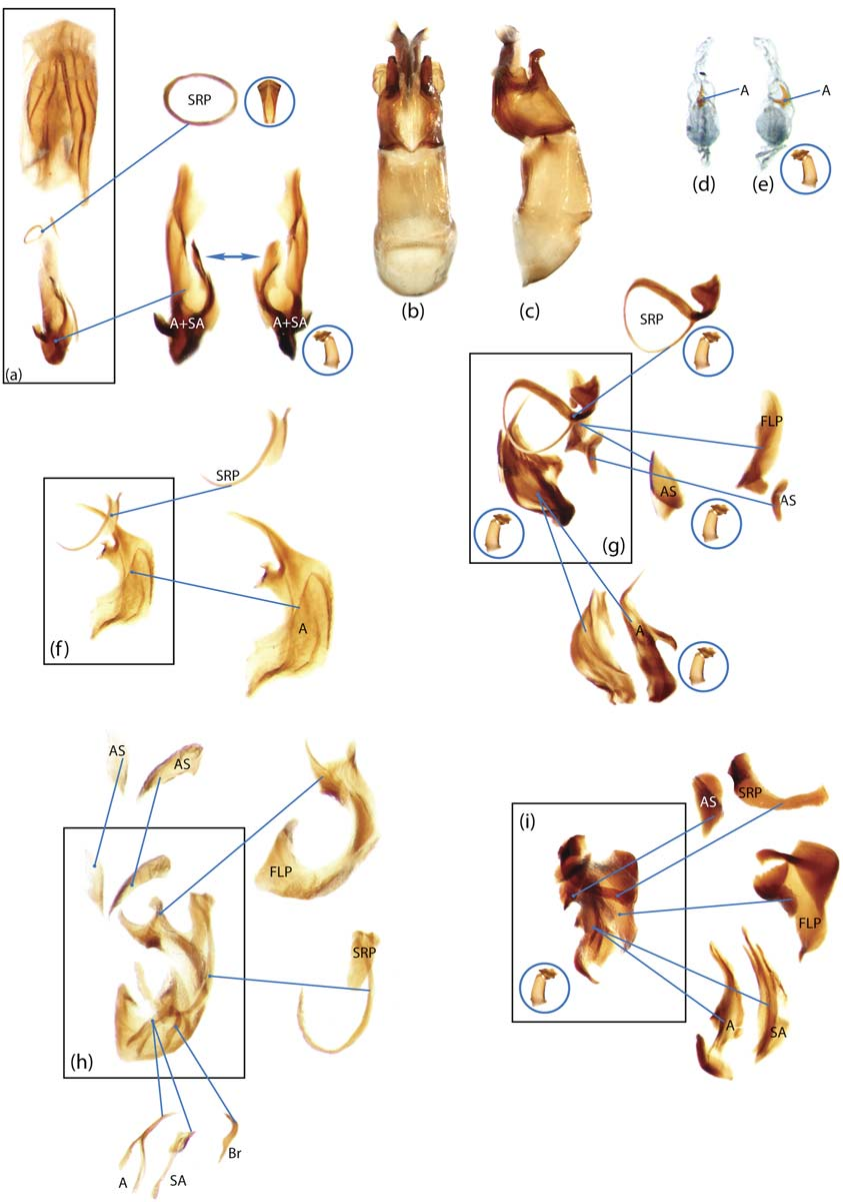
Genitalic elements of Scarabaeinae. a-c, *Amphistomus calcaratus*; d,e, *Tanzanolus sp*.; f, *Agamopus lampros*; g, *Anomiopus edmondsi*; h, *Agamopus viridis*; i, *Aphengium cupreum*; a, f-i, picture scheme of aedeagal sclerites; b, aedeagus, dorsal view; c, aedeagus, lateral left view; d, e, aedeagal sac with sclerites.

**Fig 14:**
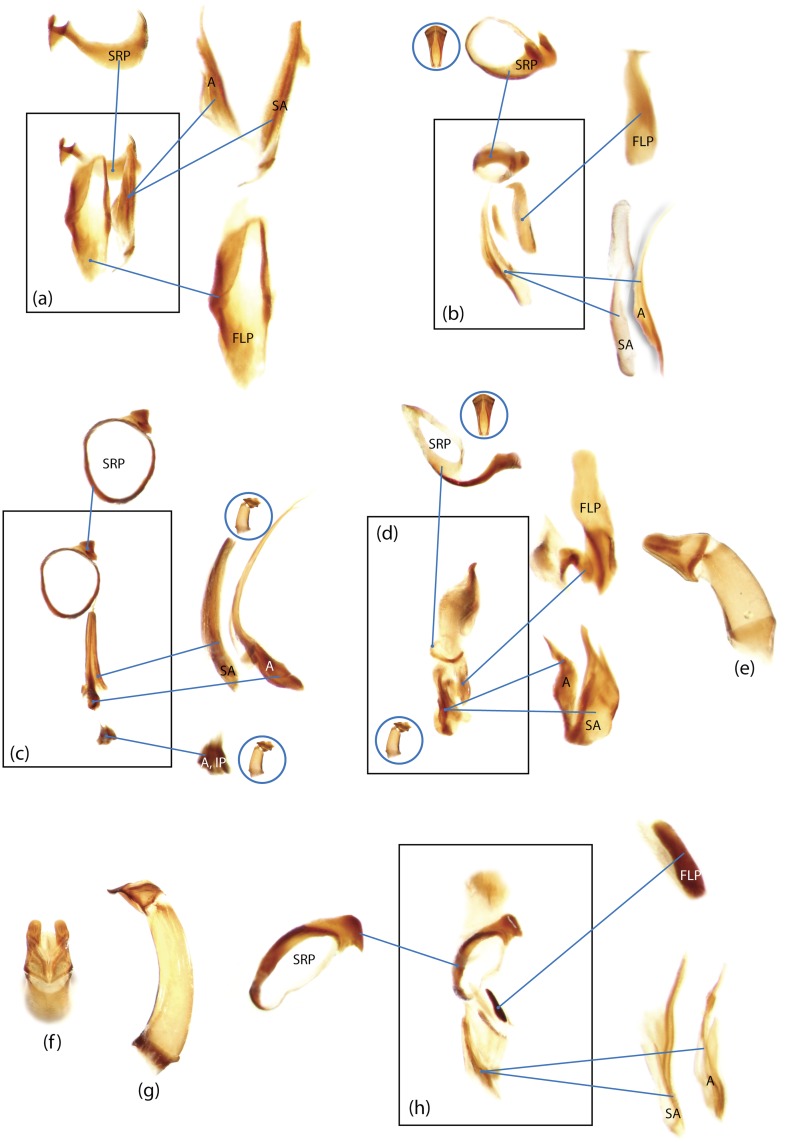
Genitalic elements of Scarabaeinae. a, *Ateuchus squalidus*; b, *Aphengoecus multiserratus*; c, *Aulacopris maximus*; d, e, *Bdelyropsis bowditchi*; f-h *Bdelyrus seminudus*; a-d, h, picture scheme of aedeagal sclerites; e, g, aedeagus, lateral left view; f, aedeagus, dorsal view.

**Fig 15:**
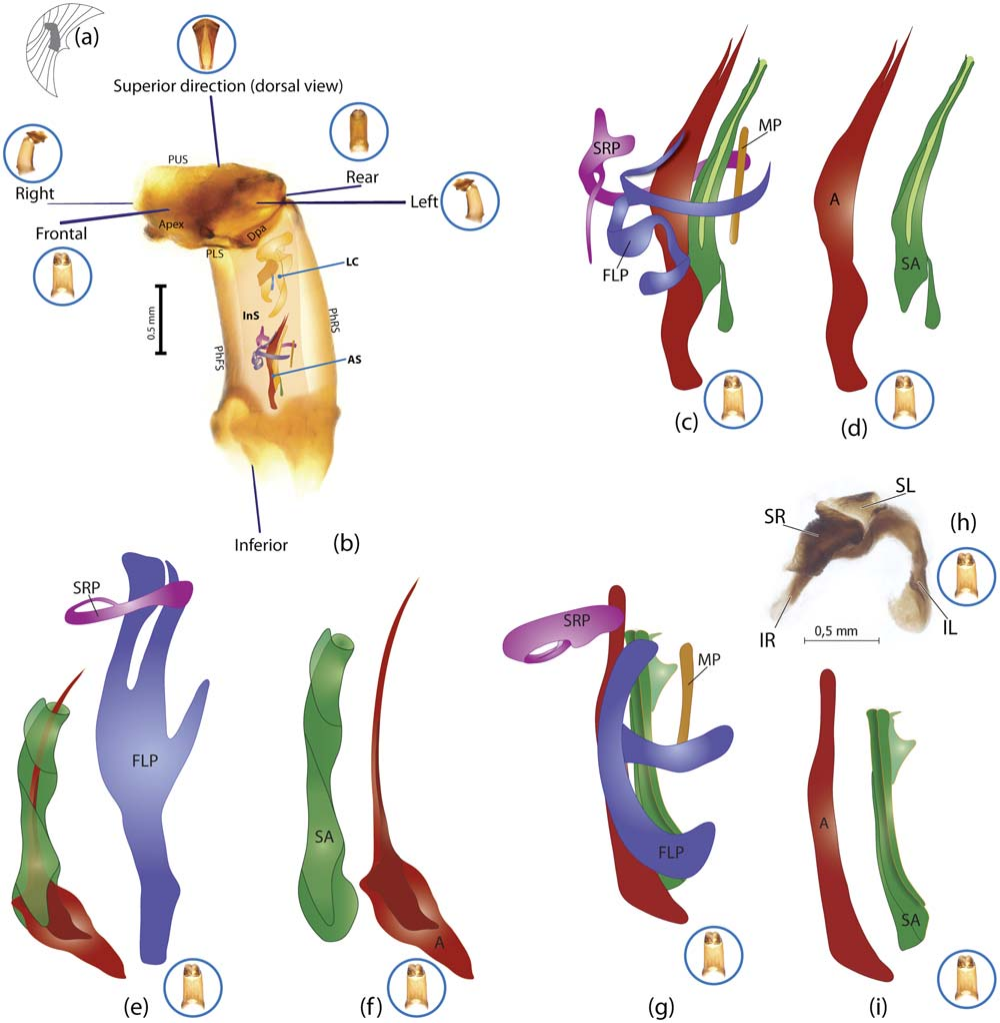
Aedeagus of *Onthophagus avocetta*. b-d, *Onthophagus avocetta*; e,f, *Bubas bison;* g, h, i, *Helictopleurus quadripunctatus*; a, position of aedeagus at rest inside abdomen, dorsal view; b, aedeagus with aedeagal sclerites, ventral-lateral oblique view. *Note*: pictures of aedeagi in blue circles are used in figures of this paper to show the relative position of aedeagus to illustrated sclerite(s). In this figure the aedeagi in blue circles are located at the tips of respective imaginary axes indicating the direction of view; the text associated with the circles indicate the terms used for describing aedeagus position and directions. c-g, i, scheme of intact aedeagal sclerites; h, lamella copulatrix.

**Fig 16:**
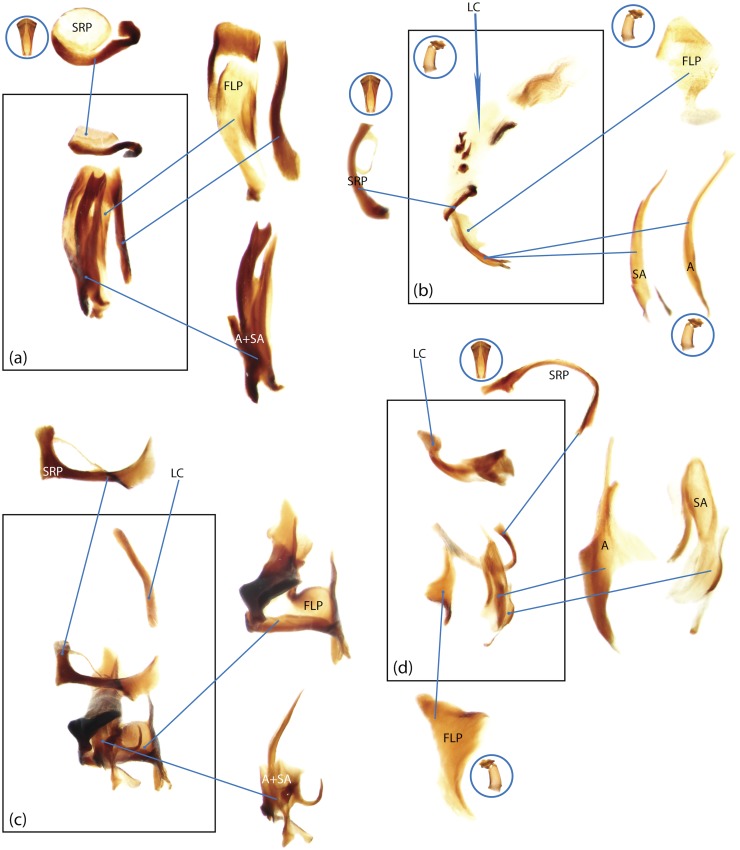
Genitalic elements of Scarabaeinae. a, *Gen*. nov. *sp*. nov.; b, *Boletoscapter cornutus*; c, *Deltochilum sp*.; d, *Canthidium bokermanni*; a-d, picture scheme of aedeagal sclerites.

**Fig 17:**
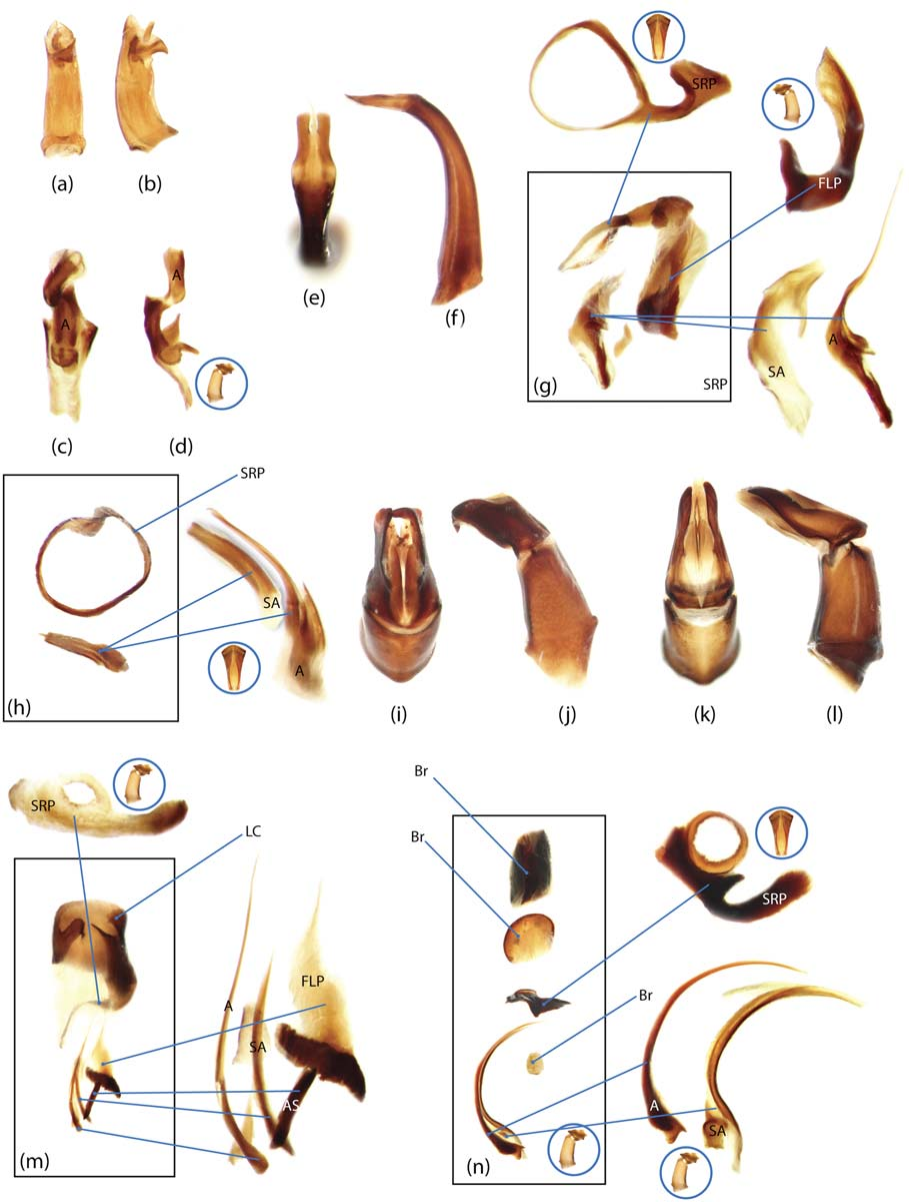
Genitalic elements of Scarabaeinae. a-d *Canthochilum tureyra*; e-f, *Canthonella silphoides*; g, *Canthon virens*; h-j, *Canthonosoma macleayi*; k-m, *Catharsius sp*.; n, *Circellium bacchus*; a, aedeagus, frontal view; b, aedeagus, right lateral view; c-d, g-h, m, n, picture scheme of aedeagal sclerites; e, i, k, aedeagus dorsal view; f, j, l, aedeagus left lateral view.

**Fig 18:**
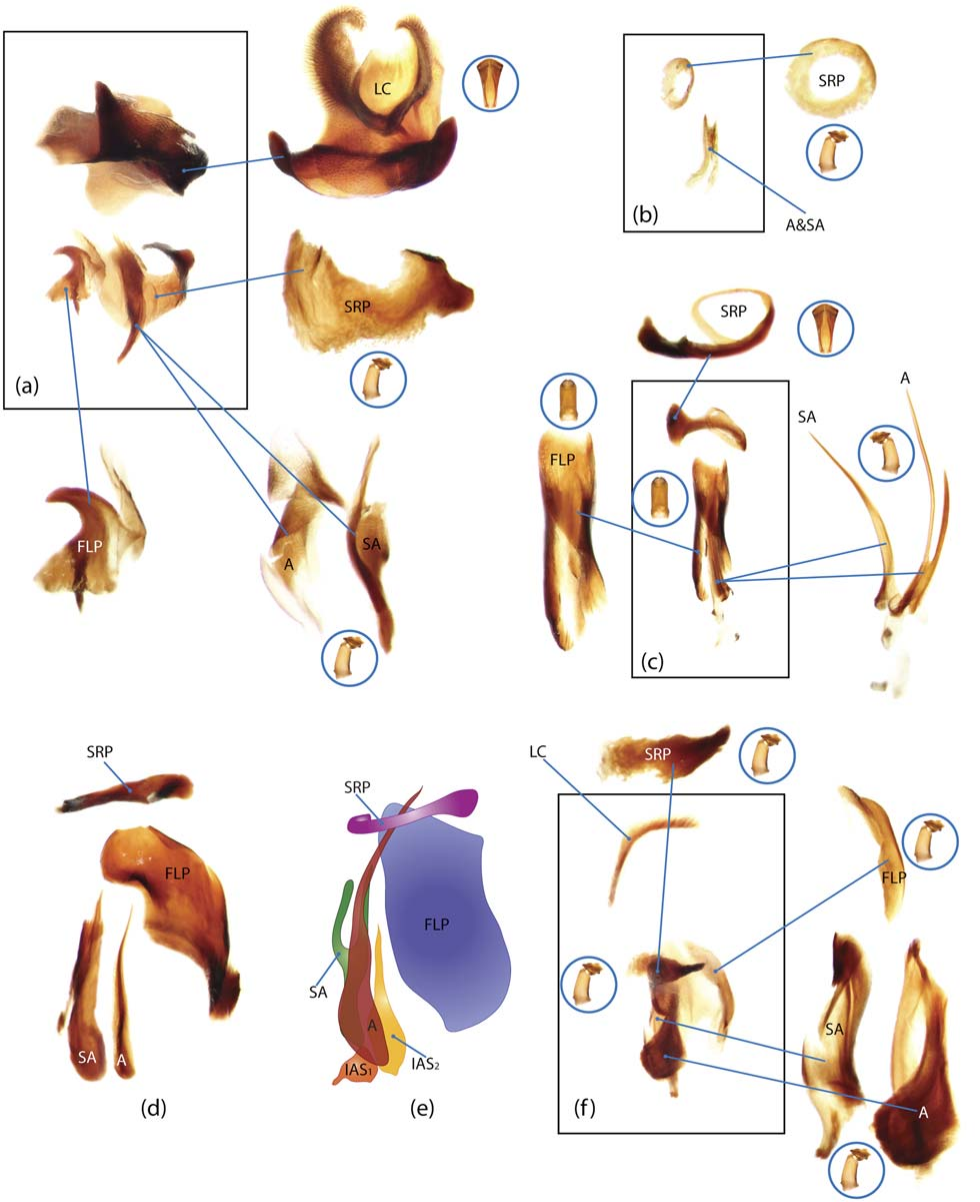
Genitalic elements of Scarabaeinae. a, *Chalcocopris hesperus*; b, *Cephalodesmius armiger*; c, *Coproecus hemisphaericus*; d, *Copris sp*.; e, *Copris*; f, *Coprophanaeus telamon*; a-c, f, picture scheme of aedeagal sclerites; d, picture scheme of aedeagal sclerites, sclerites disarticulated; e, scheme of intact aedeagal sclerites.

**Fig 19:**
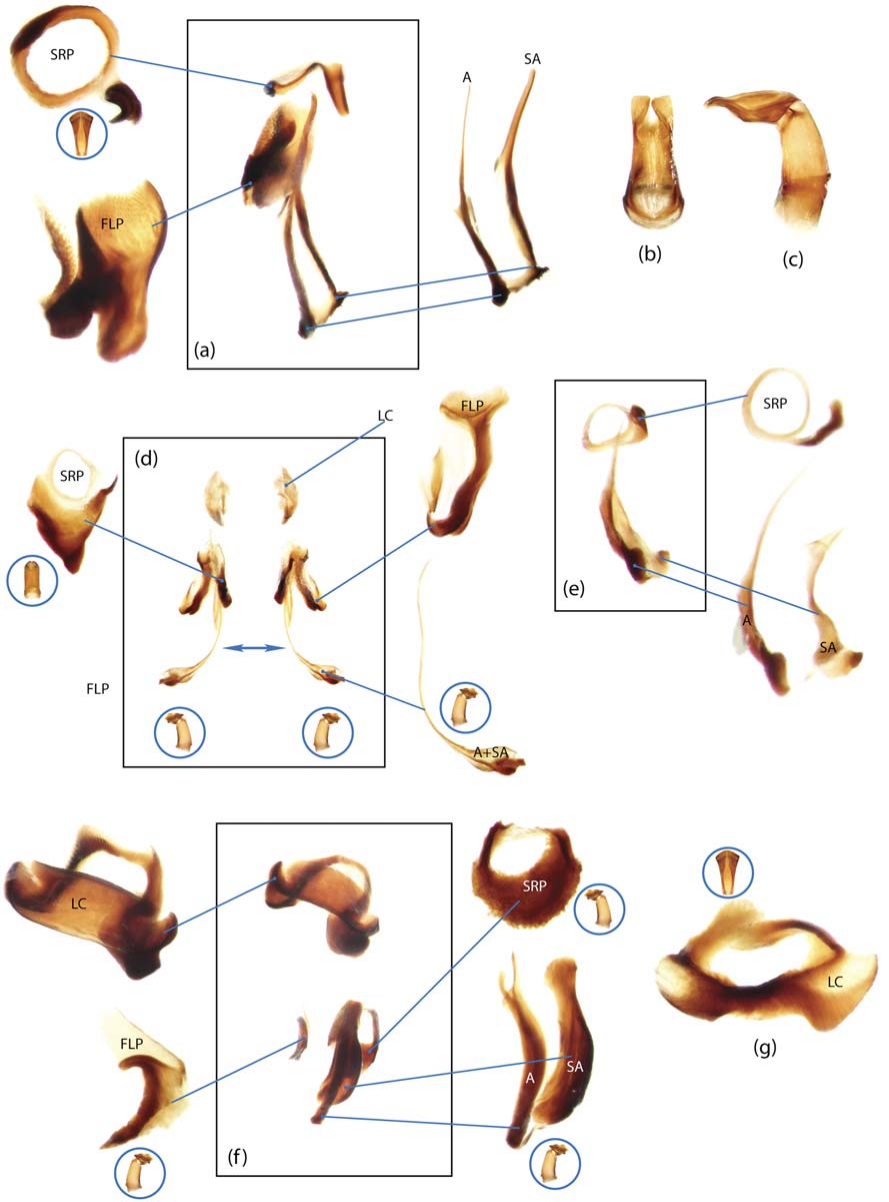
Genitalic elements of Scarabaeinae. a, *Coptodactyla nitida*; b-d, *Cryptocanthon paradoxus*; e *Demarziella interrupta*; f, *Dichotomius sericeus*; g, *Dichotomius sp*; a, d-f, picture scheme of aedeagal sclerites; b, aedeagus dorsal view; c, aedeagus left lateral view; g, lamella copulatrix.

**Fig 20:**
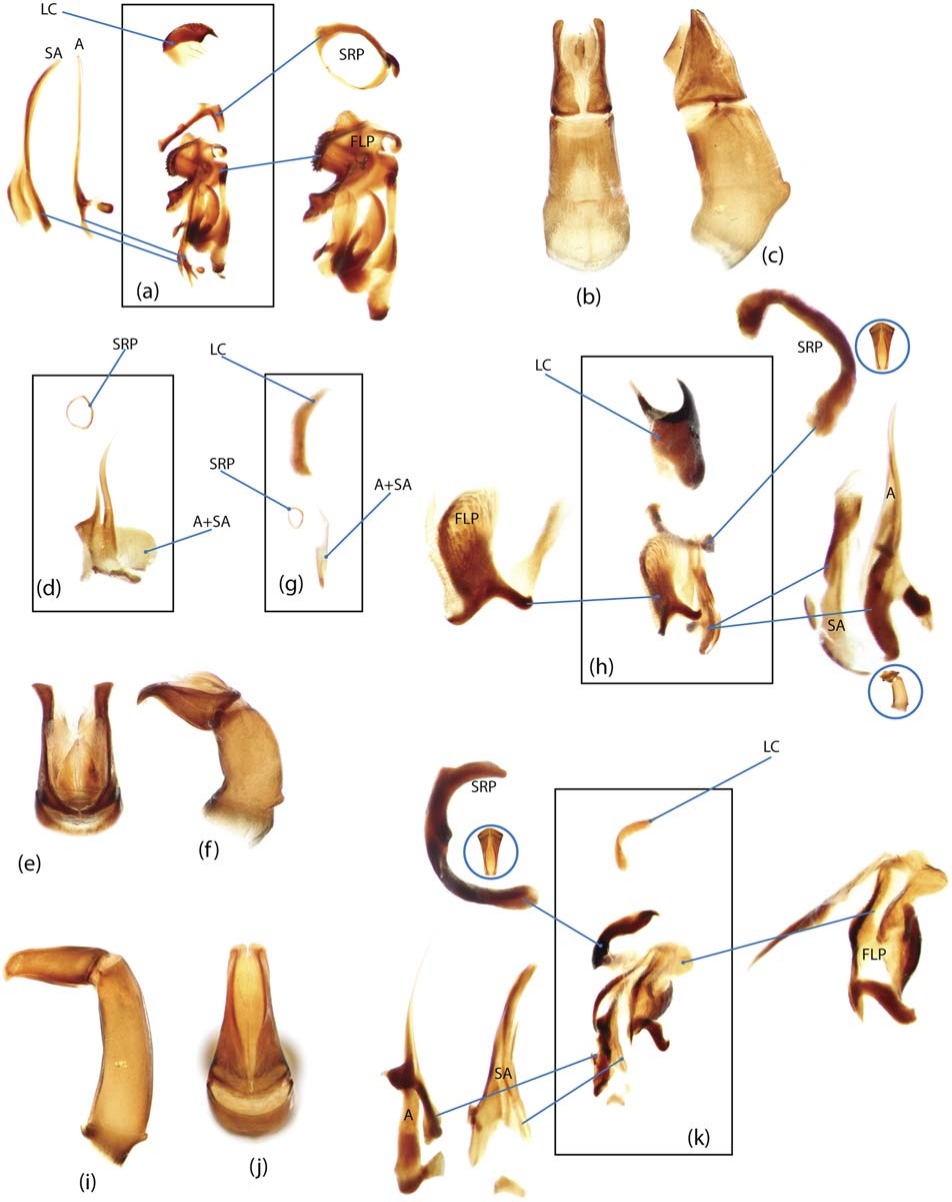
Genitalic elements of Scarabaeinae. a, *Diorygopyx tibialis*; b-c, h, *Ennearabdus lobocephalus*; d-f, *Epactoides sp1*.; g, *Epactoides sp2*.; i-k, *Eurysternus hamaticollis*; a, d, g, h, k, picture scheme of aedeagal sclerites; b, e, j, aedeagus dorsal view; c, f, i, aedeagus left lateral view.

**Fig 21:**
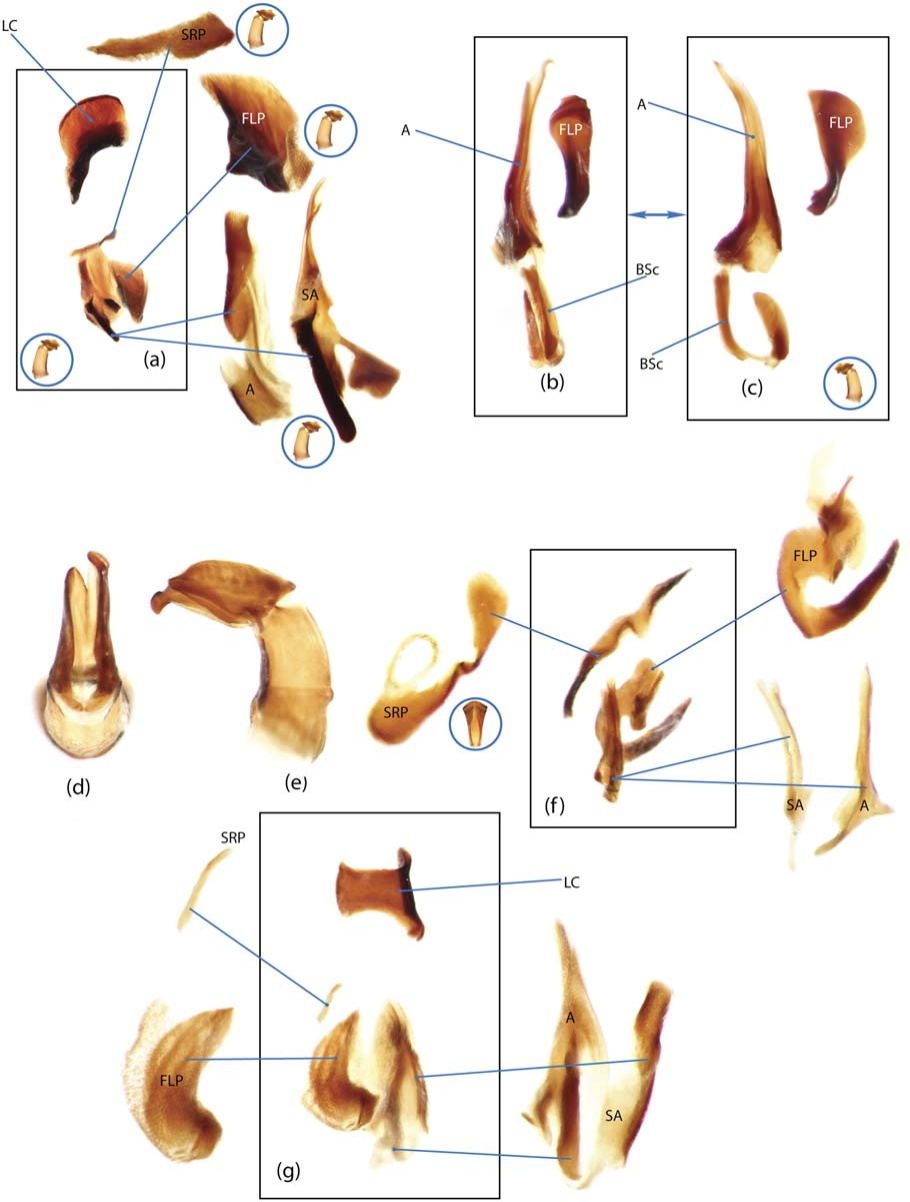
Genitalic elements of Scarabaeinae. a, *Eucranium arachnoides*; b-c, *Genieridium margareteae*; d-f, *Dwesasilvasedis medinae*; g, *Glyphoderus monticola*; a,-c, f, g, picture scheme of aedeagal sclerites; d, aedeagus dorsal view; e, aedeagus left lateral view.

**Fig 22:**
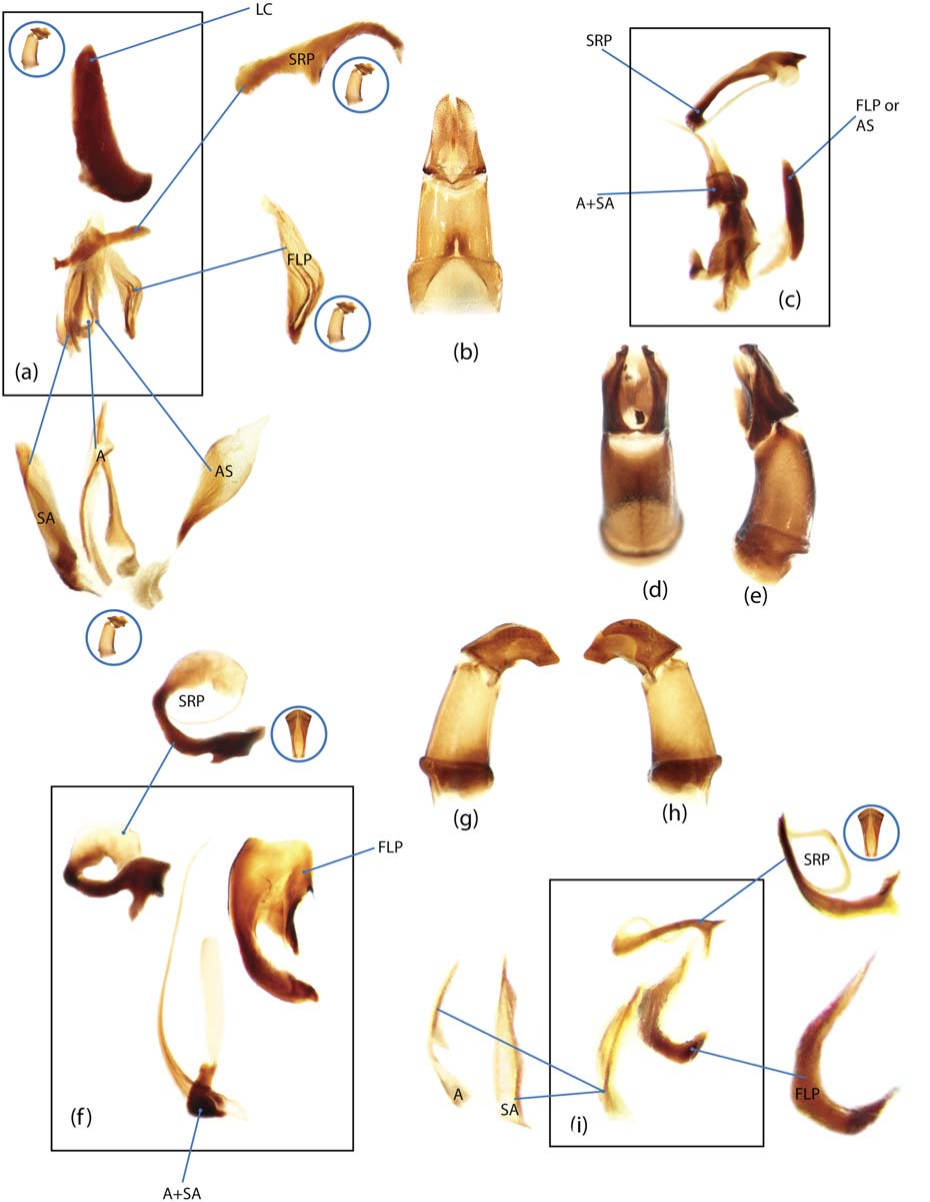
Genitalic elements of Scarabaeinae. a-b, *Gromphas aeruginosa*; c-e, *Gymnopleurus leei*; f, *Gyronotus carinatus*; g-i, *Hammondantus psammophilus*; a, c, f, i, picture scheme of aedeagal sclerites; b, d, aedeagus dorsal view; e, h, aedeagus left lateral view; g, aedeagus right lateral view.

**Fig 23:**
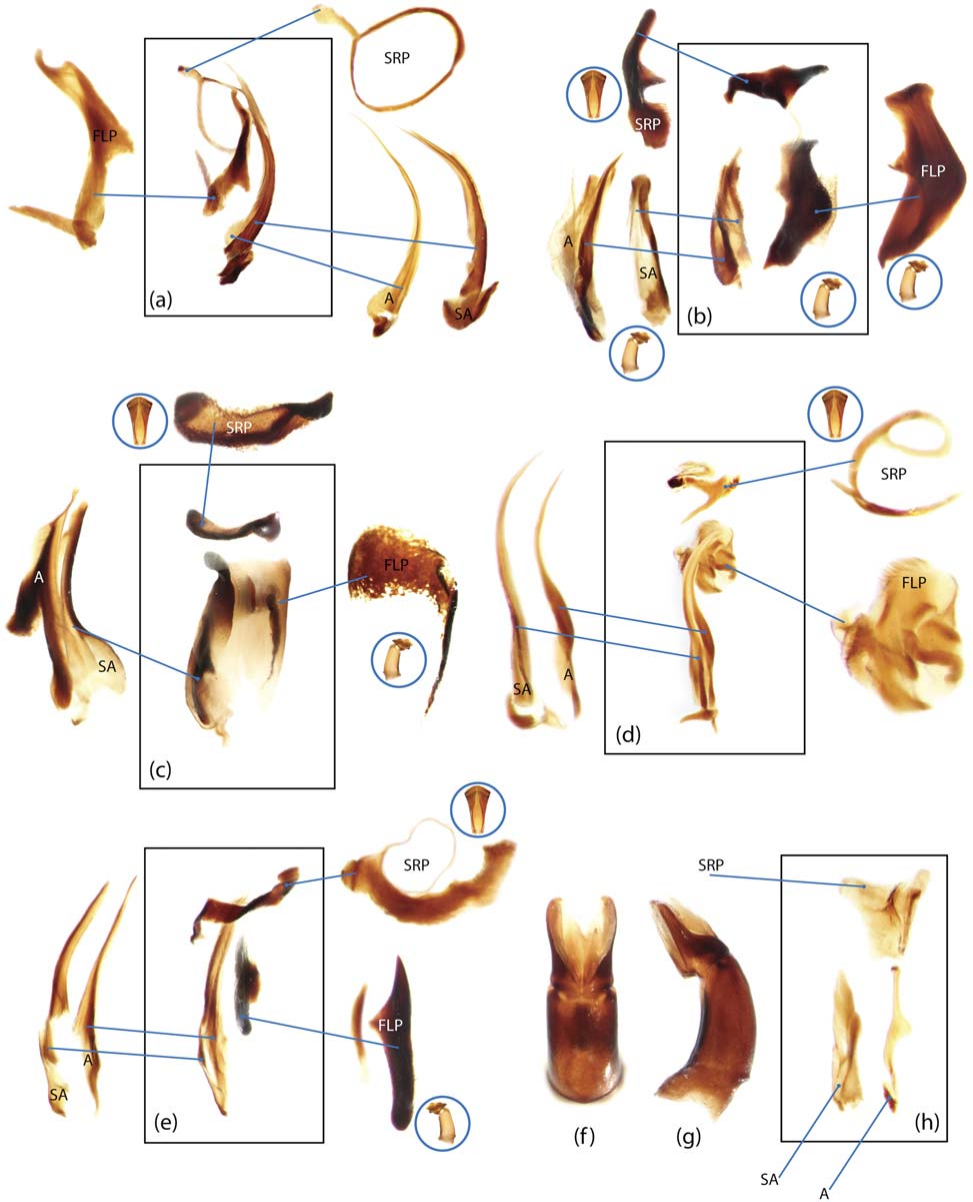
Genitalic elements of Scarabaeinae. a, *Hansreia sp*.; b, *Homocopris torulosus*; c, *Heliocopris sp*.; d, *Janssensantus pauliani*; e, *Macroderes mutilans*; f-h, *Ochicanthon sp*.; a-e, h, picture scheme of aedeagal sclerites; f, aedeagus dorsal view; g, h, aedeagus left lateral view.

**Fig 24:**
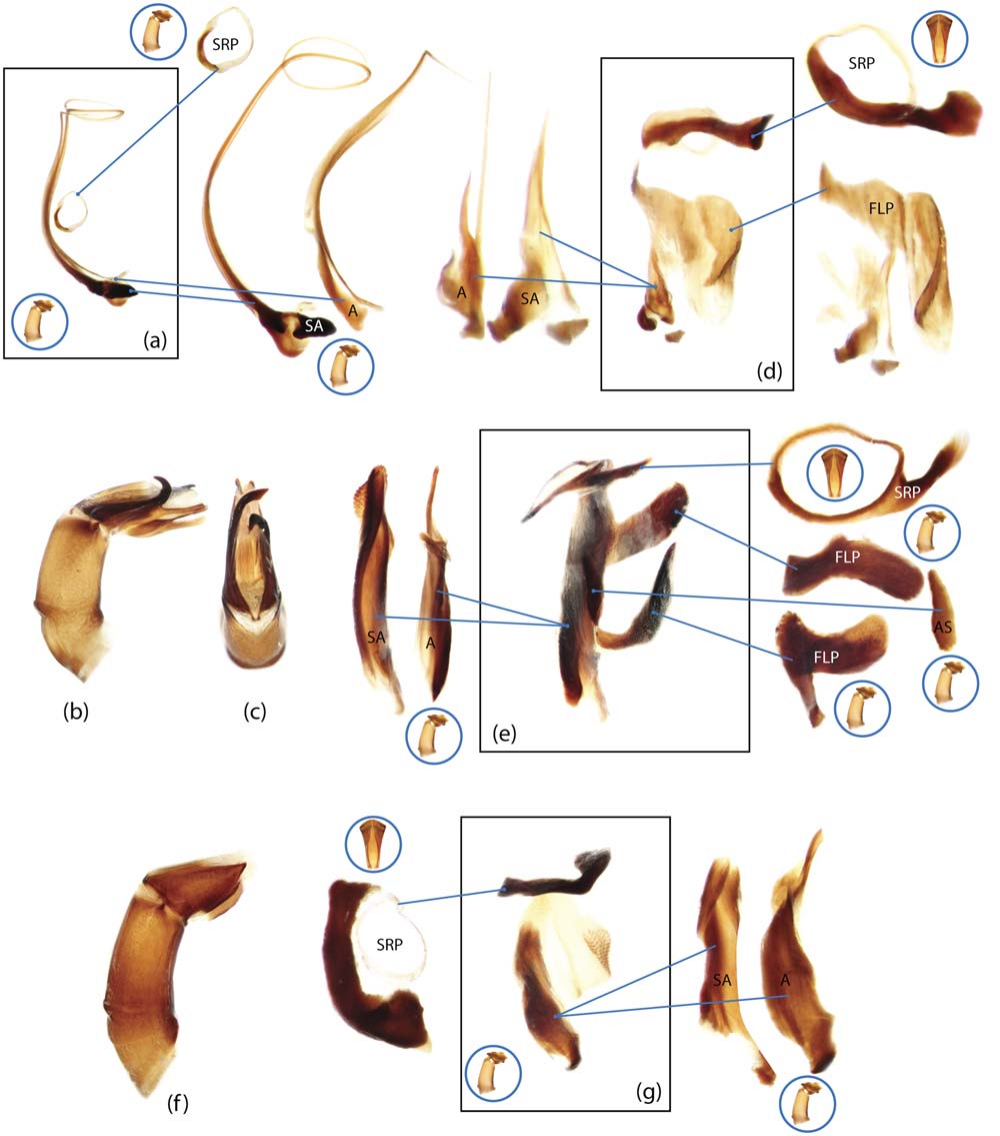
Genitalic elements of Scarabaeinae. a-c, *Arachnodes sp*.; d, *Mentophilus hollandiae*; e, *Megathoposoma candezei*; f-g, *Malagoniella yucateca*; a, d, e, g, picture scheme of aedeagal sclerites; b, f, aedeagus right lateral view; c, aedeagus dorsal view.

**Fig 25:**
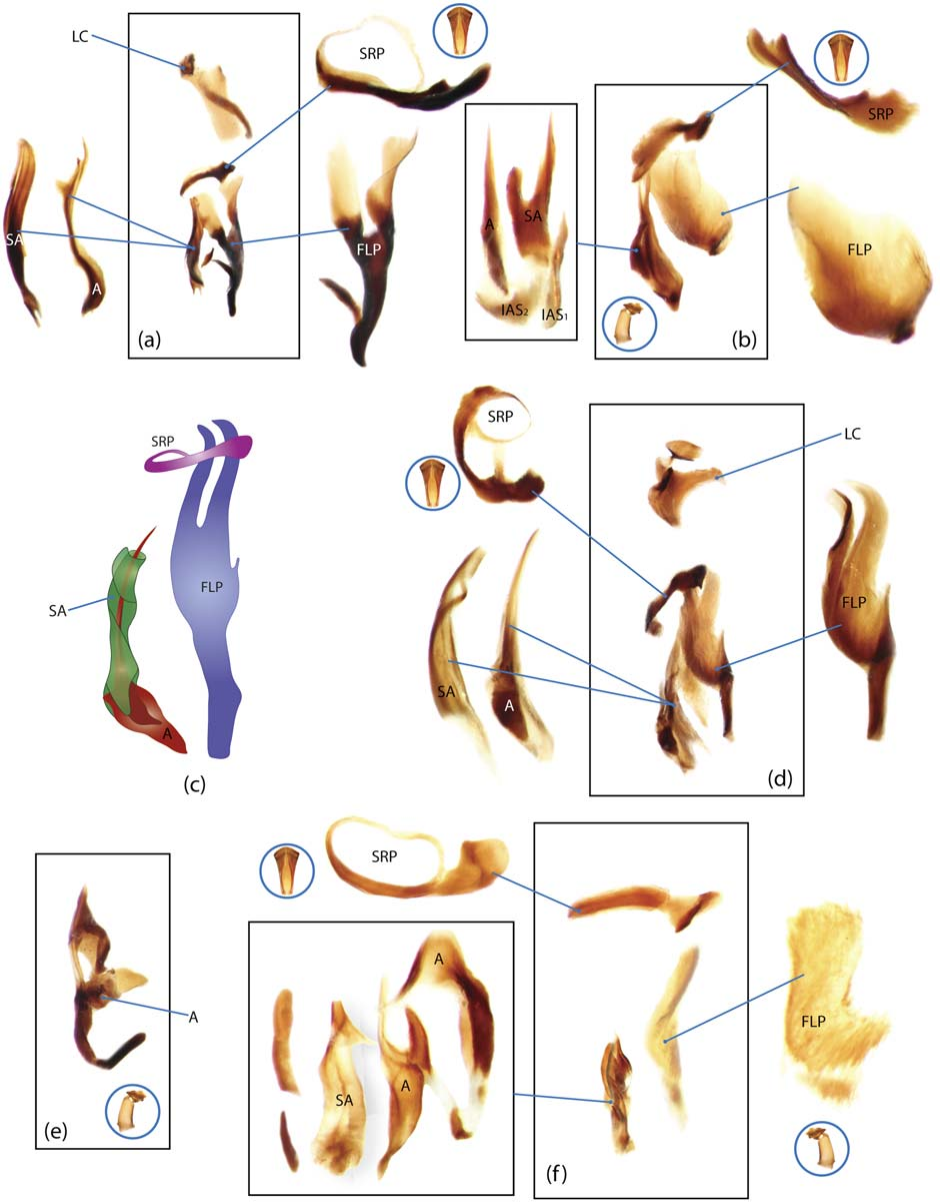
Genitalic elements of Scarabaeinae. a, *Metacatharsius sp*.; b, *Microcopris sp*.; c, *Onitis*; d, *Onitis sp*.; e, *Onoreidium howdeni*; f, *Onthobium gutierrezi*; a, b, d-f, picture scheme of aedeagal sclerites; c, scheme of aedeagal sclerites.

**Fig 26:**
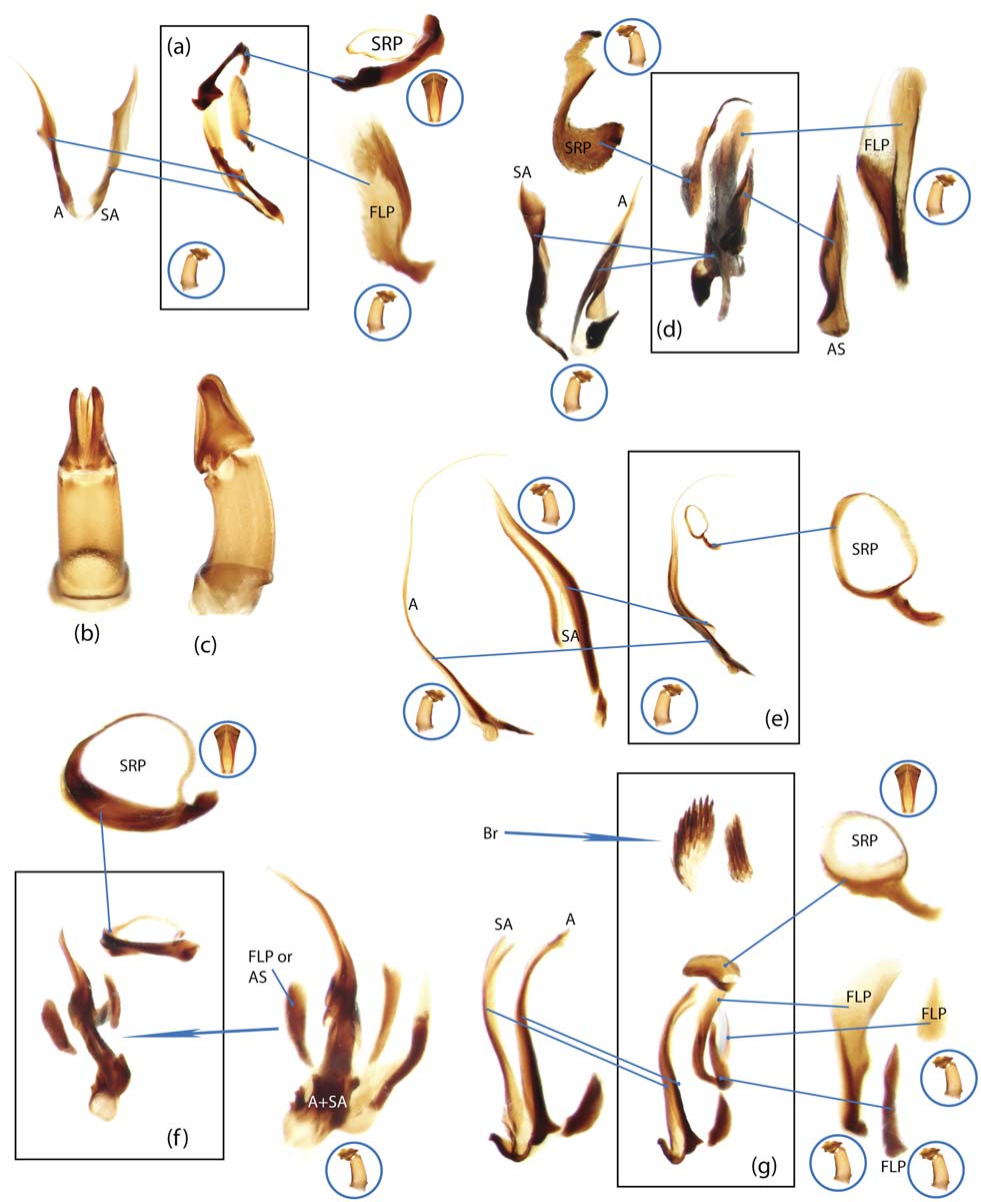
Genitalic elements of Scarabaeinae. a-c, *Ontherus appendiculatus*; d, *Oruscatus davus*; e, *Scarabaeus aesculapius*; f, *Paragymnopleurus sp*.; g, *Pedaria sp*.; a, d, e, f, g, picture scheme of aedeagal sclerites; b, aedeagus dorsal view. b, aedeagus left lateral view.

**Fig 27:**
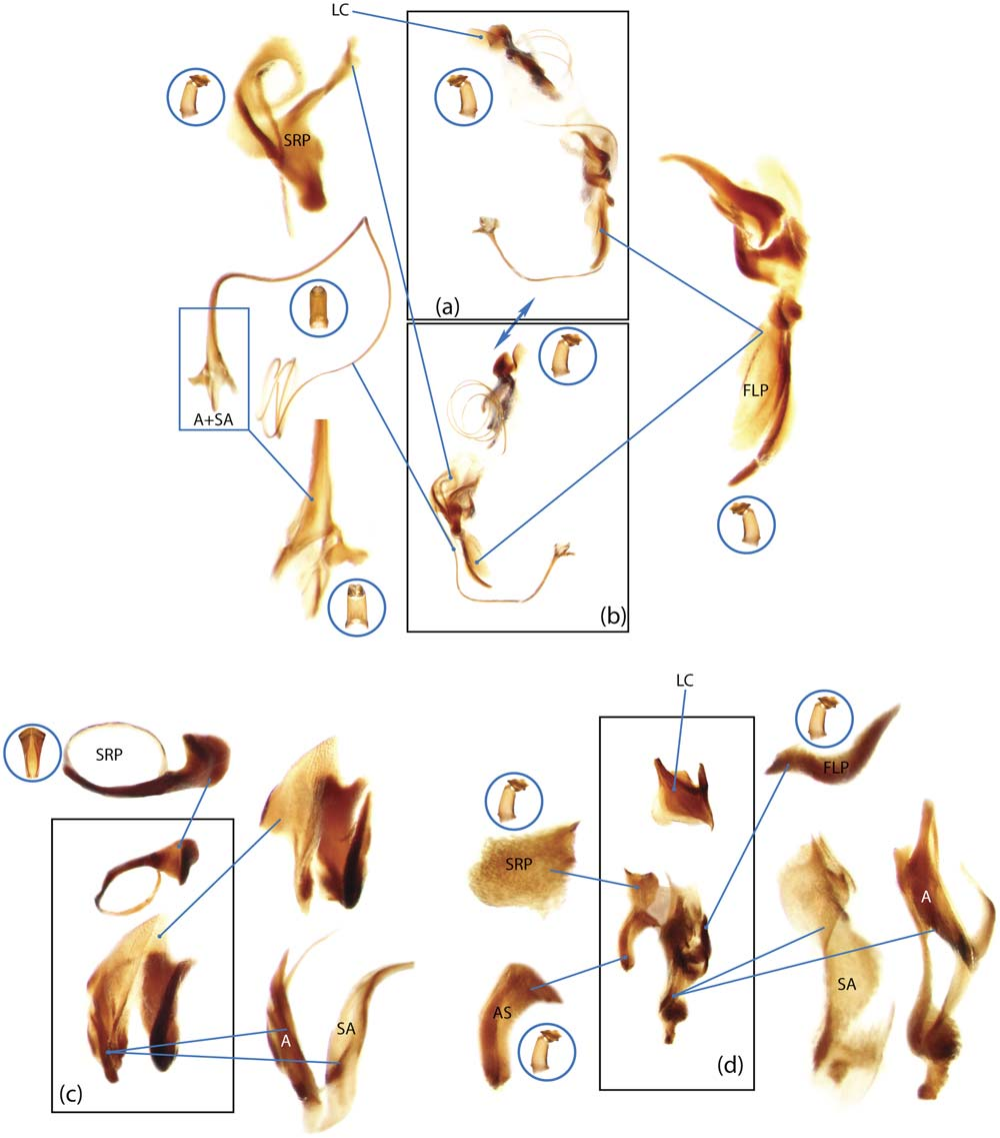
Genitalic elements of Scarabaeinae. a, b, *Paracanthon sp*.; c, *Pseudonthobium sinuatotibiale*; d, *Phanaeus splendidulus*; a-d, picture scheme of aedeagal sclerites.

**Fig 28:**
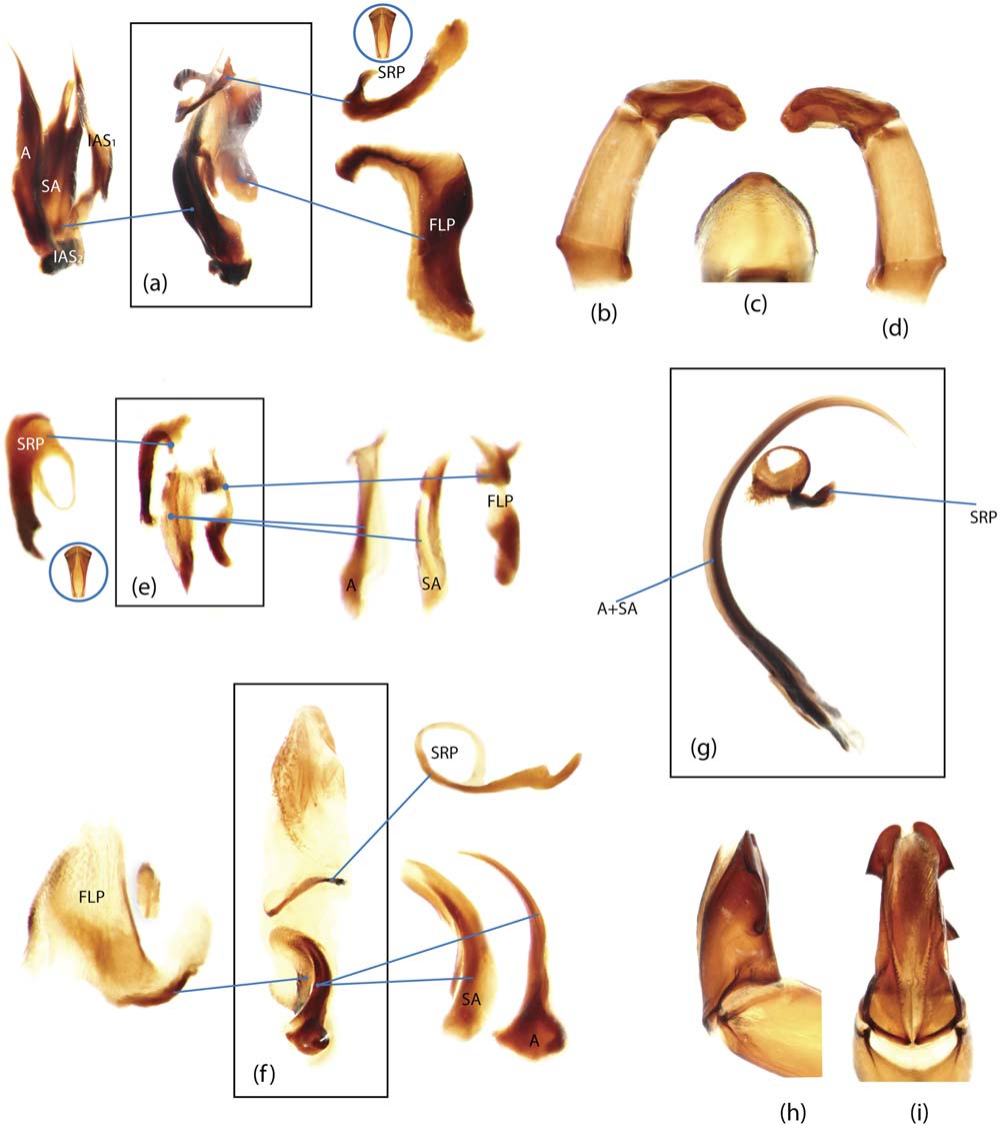
Genitalic elements of Scarabaeinae. a, *Pseudopedaria grossa*; b-e, *Pycnopanelus krikkeni*; f, *Saphobius sp*.; g-i, *Scarabaeus aegyptiorum*; a, e, g, f, picture scheme of aedeagal sclerites; b, h, aedeagus, right lateral view; c, aedeagus, phallobase, dorsal view; d, aedeagus, left lateral view; i, aedeagus, dorsal view.

**Fig 29:**
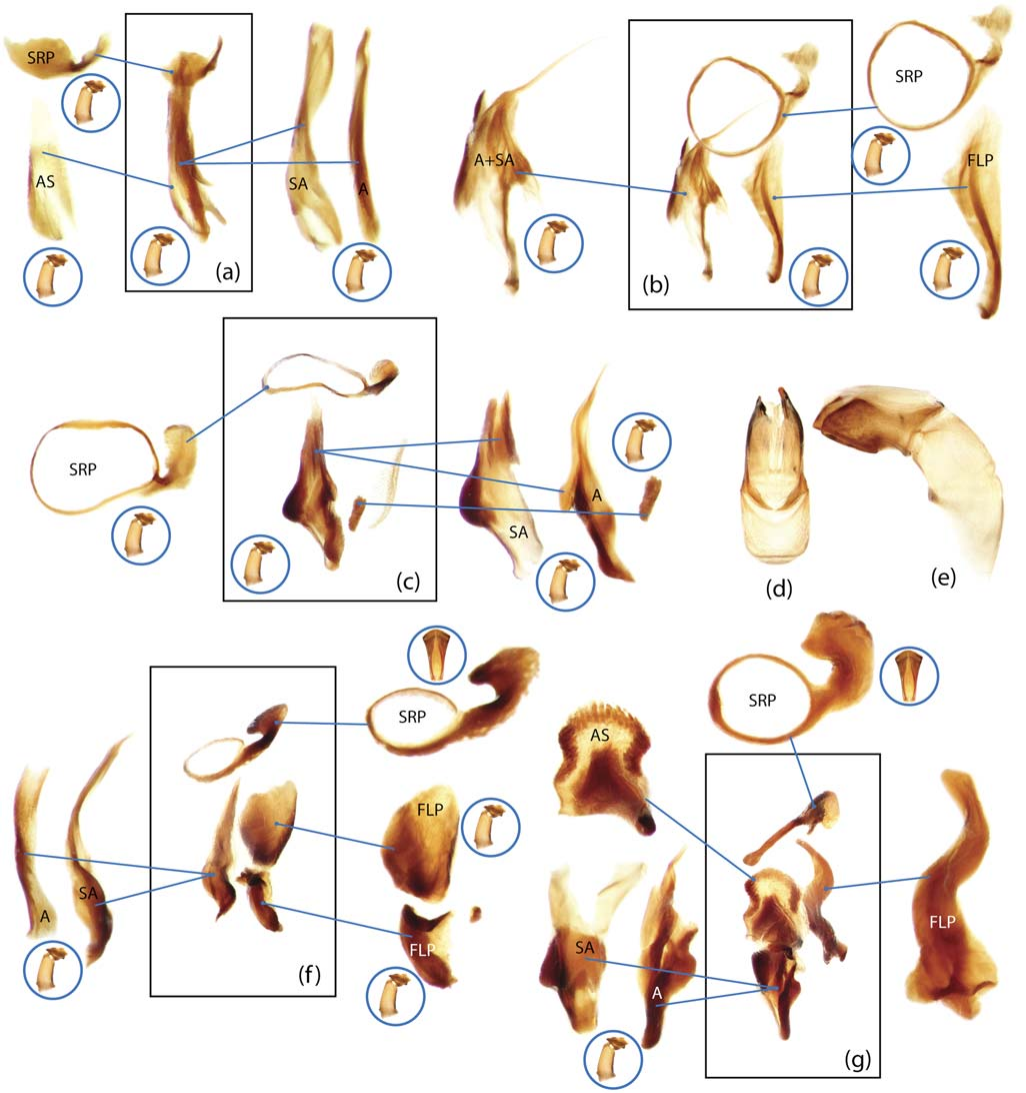
Genitalic elements of Scarabaeinae. a, *Scatimus strandi*; b, *Scatonomus fasciculatus*; c-e, *Scybalocanthon nigriceps*; f, *Scybalophagus rugosus*; g, *Sylvicanthon bridarollii*; a-c, f, g, picture scheme of aedeagal sclerites; d, aedeagus, dorsal view; e, aedeagus, left lateral view.

**Fig 30:**
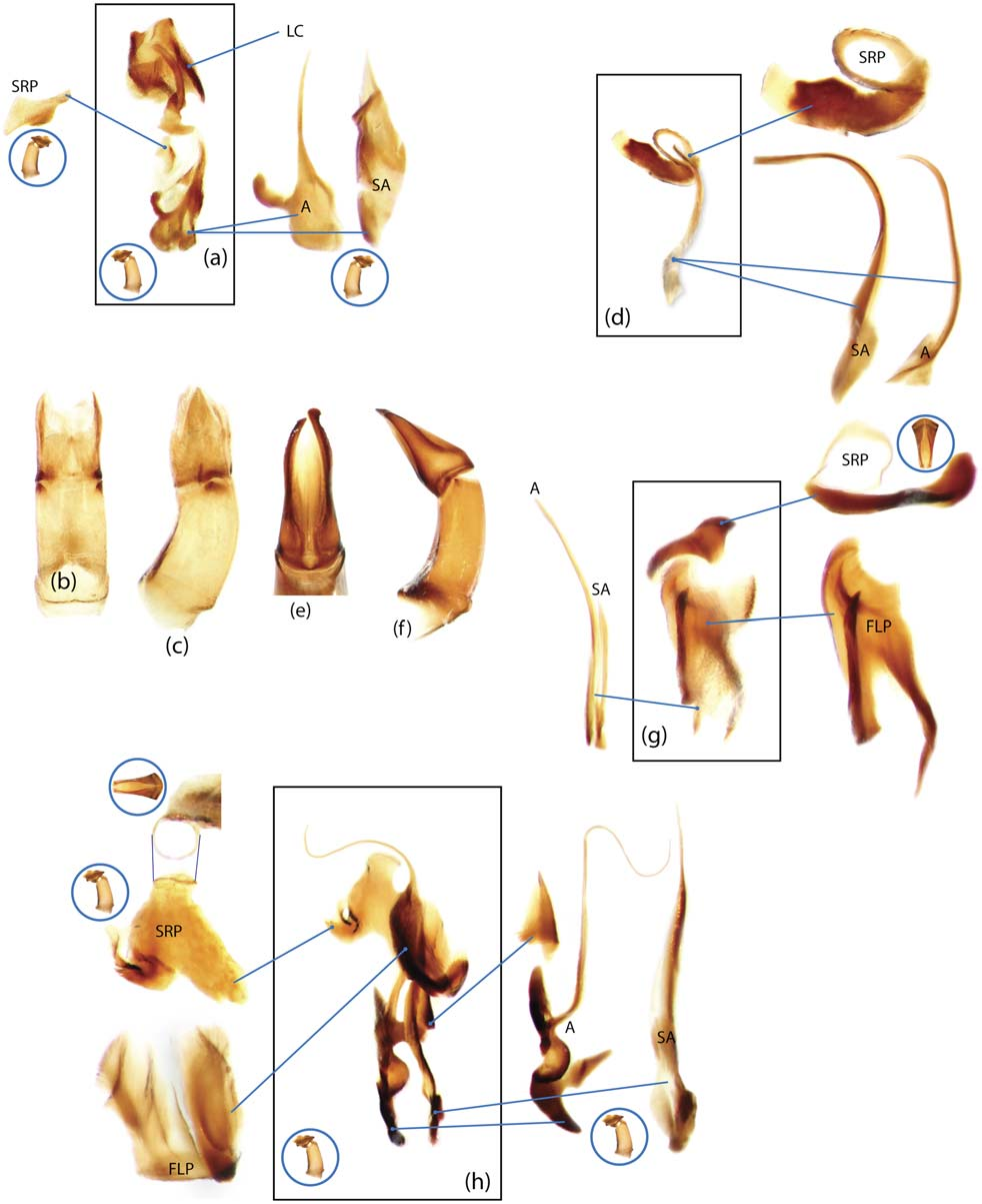
Genitalic elements of Scarabaeinae. a-c, *Sinapisoma sp*.; d-f, *Streblopus opatroides*; g, *Tesserodon erratum*; h, *Neosisyphus sp*.; a, d, g, h, picture scheme of aedeagal sclerites; b, e, aedeagus, dorsal view; c, f, aedeagus, left lateral view.

**Fig 31:**
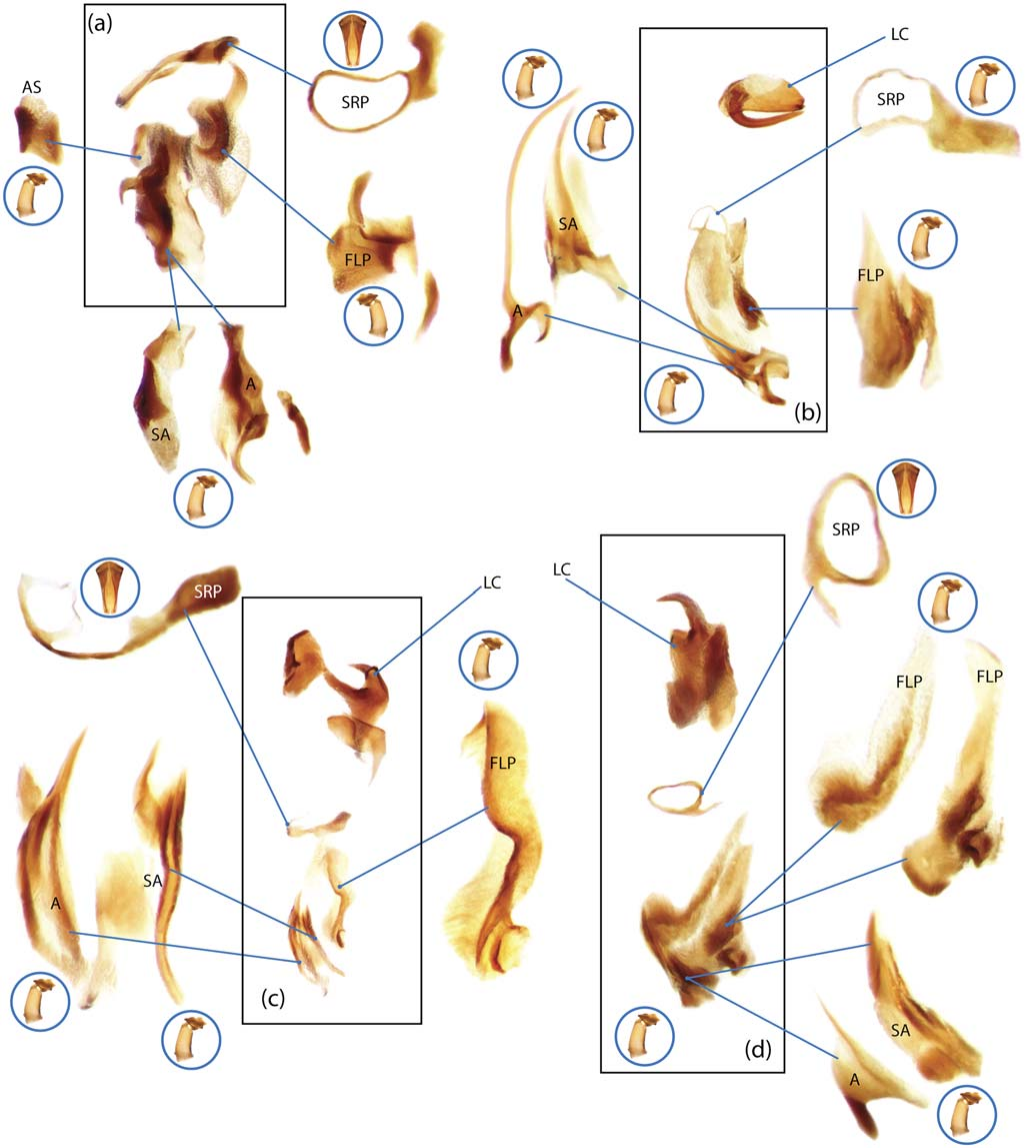
Genitalic elements of Scarabaeinae. a, *Tetraechma sanguineomaculata*; b, *Uroxys epipleuralis*; c, *Uroxys latesulcatus*; d, *Uroxys pauliani*; a-d, picture scheme of aedeagal sclerites.

**Fig 32:**
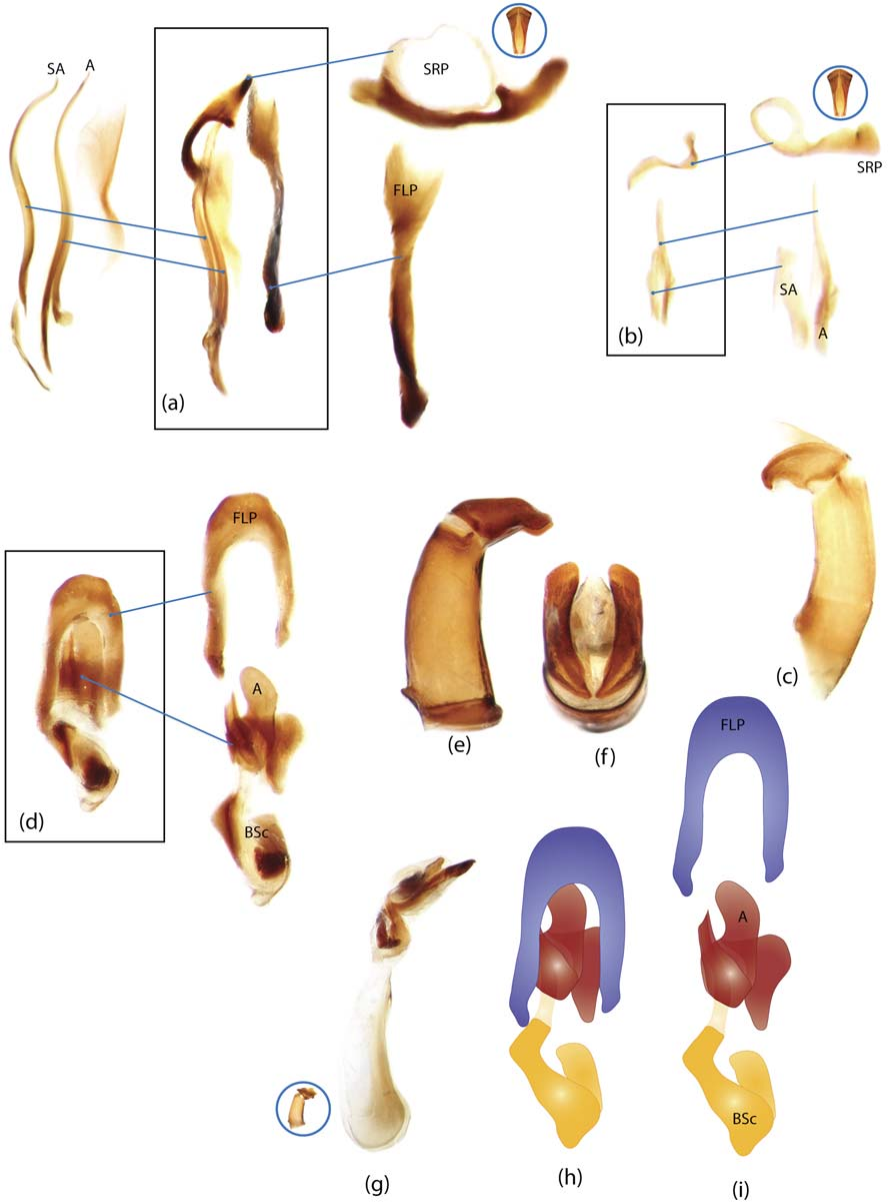
Genitalic elements of Scarabaeinae. a, *Xinidium dentilabris*; b-c, *Zonocopris gibbicollis*; d-i, *Trichillum pauliani*; a, b, d, picture scheme of aedeagal sclerites; c, aedeagus right lateral view; f, aedeagus dorsal view; c, aedeagus left lateral view; g, intact aedeagal sac with sclerites; h, i, scheme of aedeagal sclerites.

**Fig 33:**
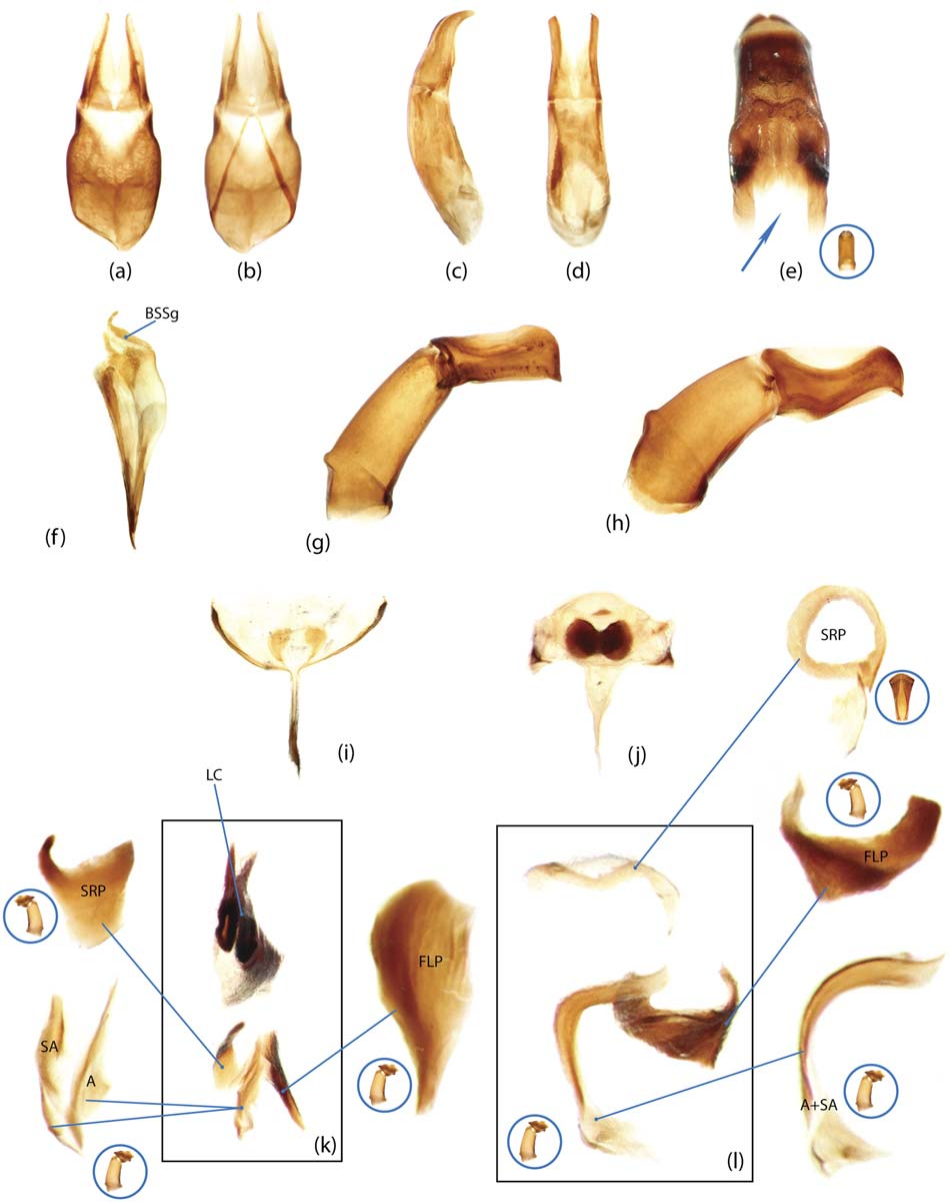
Genitalic elements of Scarabaeinae. a, b, *Aphodius erraticus*; c, d, *Endroedyolus paradoxus*; e, *Epirinus sp*.; f, *Podotenus fulviventris*; g, *Xinidium dentilabris*; h, *Macroderes mutilans*; i, *Dicranocara deschodti*; j, *Demarziella interrupta*; l, *Tesserodoniella elguetai;* k, *Ateuchus histeroides*; a, d, aedeagus dorsal view; b, aedeagus ventral view; c, g, h, aedeagus lateral view; e, aedeagus rear view; f, spiculum gastrale, lateral view; i, j, spiculum gastrale; k. l, picture scheme of aedeagal sclerites.

The aedeagus lies on its lateral side inside the abdomen (Fig. 15a), which complicates positional description of aedeagal elements in respect to the body. Thus, we use symmetry of the aedeagus itself to describe relative positions of its elements. The explanation of these terms is given in Fig. 15b. The picture of the aedeagus in the blue circle next to sclerite pictures indicates the location of the aedeagus during photographing. If no blue circle picture is linked with the sclerite picture then the position of the aedeagus is frontal (see Fig. 15b). The green arrows are used to align elements of a sclerite that were disarticulated for illustration purposes. The double-headed arrows are used to indicate the same structure illustrated from different angles.

The notes for the illustrations of the somatic characters (Figs. 34–47) are provided in their legends.

**Fig 34:**
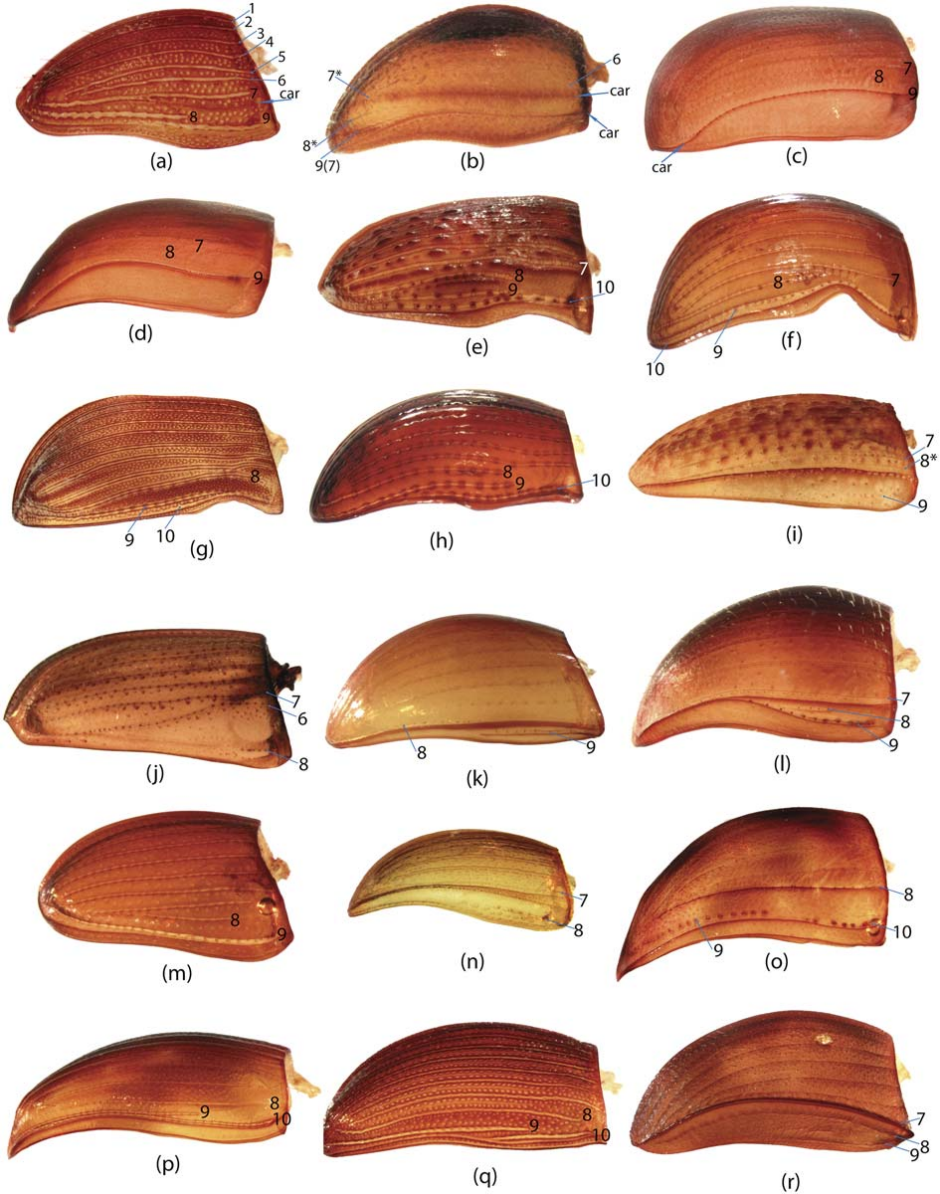
Elytra of Scarabaeinae. a, *Odontoloma sp*.; b, *Endroedyolus paradoxus*; c, *Dicranocara deschodti*; d, *Byrrhidium namaquensis*; e, *Frankenbergerius armatus*; f, *Delopleurus sp*.; g, *Sarophorus costatus*; h, *Paraphytus sp*.; i, *Epirinus sp*.; j, *Neosisyphus sp*.; k, *Janssensantus pauliani*; l, *Aphengoecus multiserratus*; m, *Pycnopanelus krikkeni*; n, *Bohepilissus sp*.; o, *Macroderes mutilans*; p, *Xinidium dentilabris*; q, *Pedaria sp*.; r, *Gyronotus carinatus*; a-r, right elytron, lateral view. Elytral striae are enumerated; indistinct striae or their traces are marked by *.

**Fig 35:**
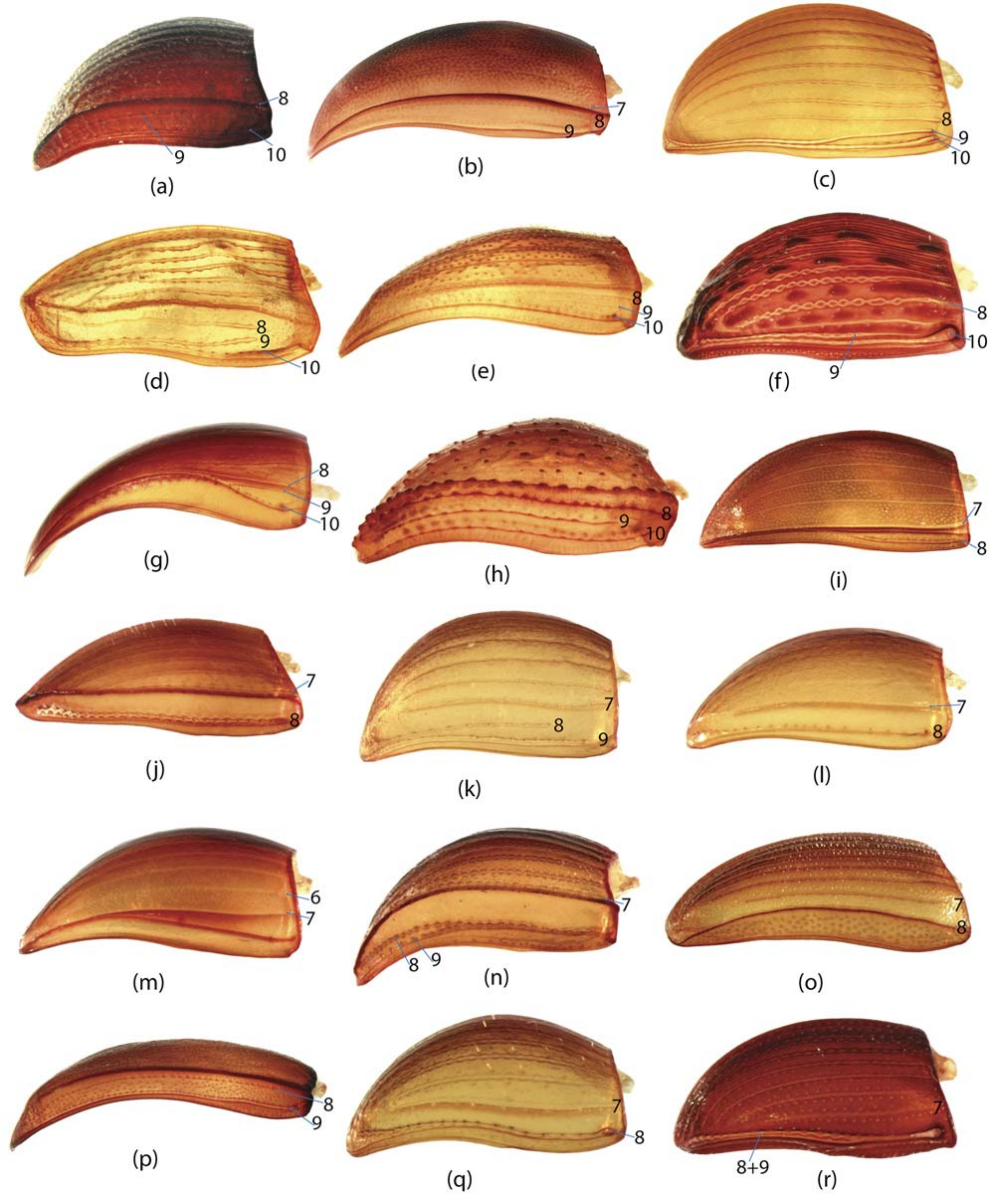
Elytra of Scarabaeinae. a, *Dwesasilvasedis medinae*; b, *Anachalcos convexus*; c, *Coptodactyla nitida*; d, *Amphistomus calcaratus*; e, *Boletoscapter cornutus*; f, *Demarziella interrupta*; g, *Diorygopyx tibialis*; h, *Onthobium gutierrezi*; i, *Bdelyrus seminudus*; j, *Bdelyropsis bowditchi*; k, *Tesserodoniella elguetai*; l, *Canthochilum tureyra*; m, *Canthonella silphoides*; n, *Cryptocanthon paradoxus*; o, *Paracanthon sp*.; p, *Streblopus opatroides*; q, *Zonocopris gibbicollis*; r, *Genieridium margareteae*; a-r, right elytron, lateral view. Elytral striae are enumerated; indistinct striae or their traces are marked by *.

**Fig 36:**
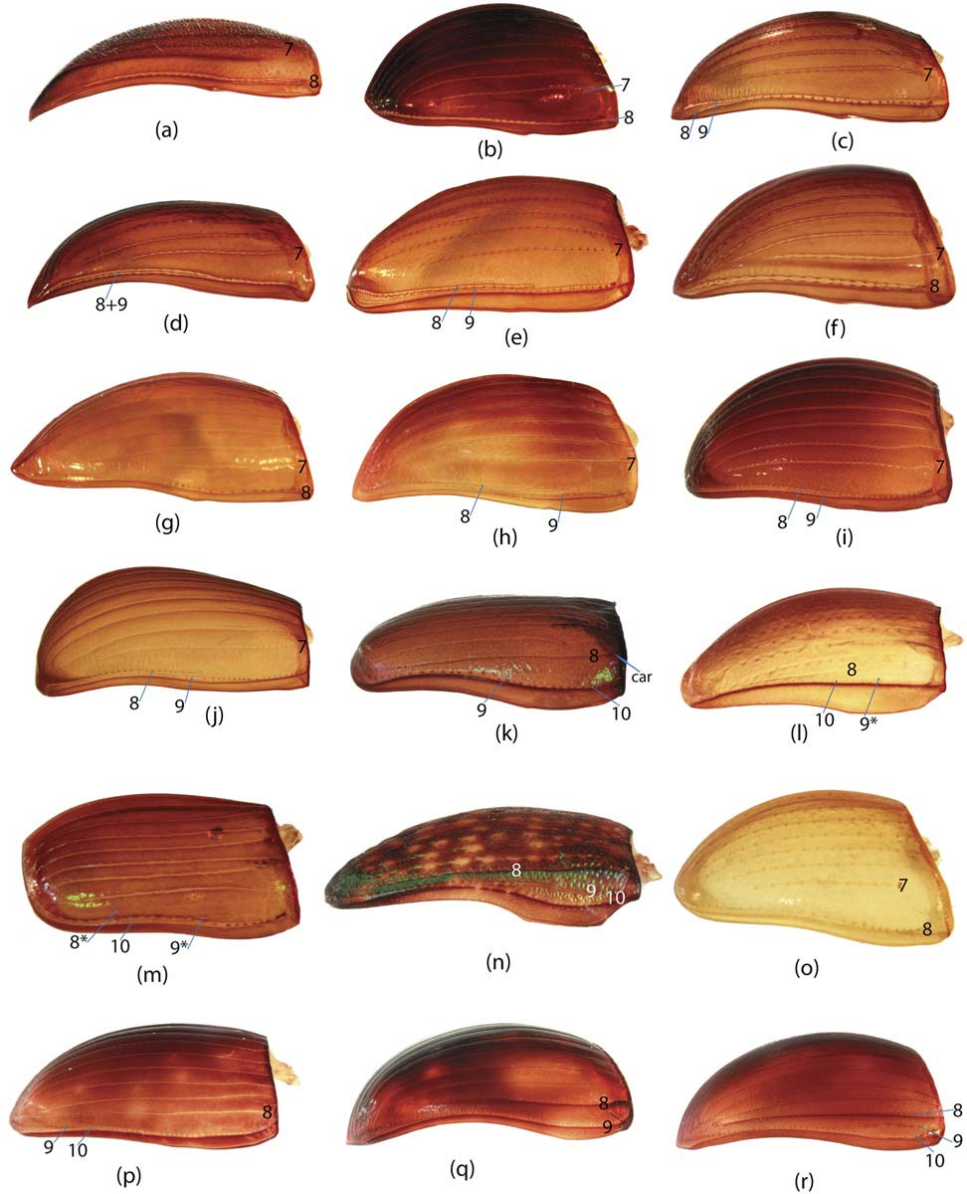
Elytra of Scarabaeinae. a, *Aphengium cupreum*; b, *Ateuchus sp1*.; c, *Ateuchus sp2*.; d, *Scatimus strandi*; e, *Canthidium bokermanni*; f, *Uroxys epipleuralis*; g, *Uroxys pauliani*; h, *Homocopris torulosus*; i, *Ontherus appendiculatus*; j, *Uroxys latesulcatus*; k, *Canthon virens*; l, *Hansreia sp*.; m, *Anomiopus edmondsi*; n, *Megathoposoma candezei*; o, *Sinapisoma sp*.; p, *Copris sp*.; q, *Catharsius sp*.; r, *Metacatharsius sp*.; a-r, right elytron, lateral view. Elytral striae are enumerated; indistinct striae or their traces are marked by *.

**Fig 37:**
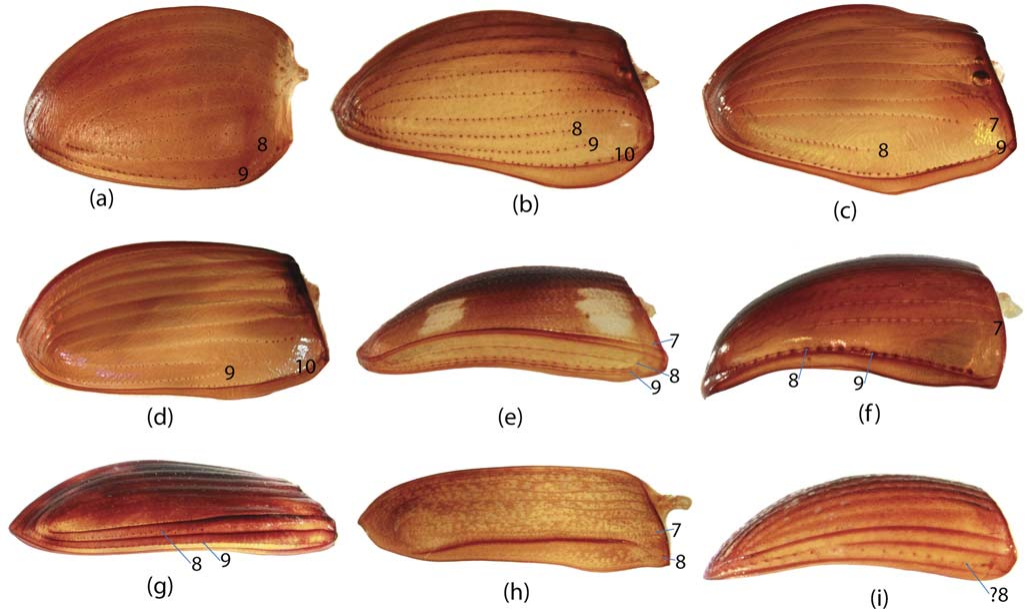
Elytra of Scarabaeinae. a, *Eucranium arachnoides*; b, *Ennearabdus lobocephalus*; c, *Gromphas aeruginosa*; d, *Oruscatus davus*; e, *Ochicanthon sp*.; f, *Arachnodes sp*.*;* g, *Scarabaeus sp*.; h, *Eurysternus hamaticollis*; i, *Tanzanolus sp*.; a-i, right elytron, lateral view. Elytral striae are enumerated; indistinct striae or their traces are marked by *.

**Fig 38:**
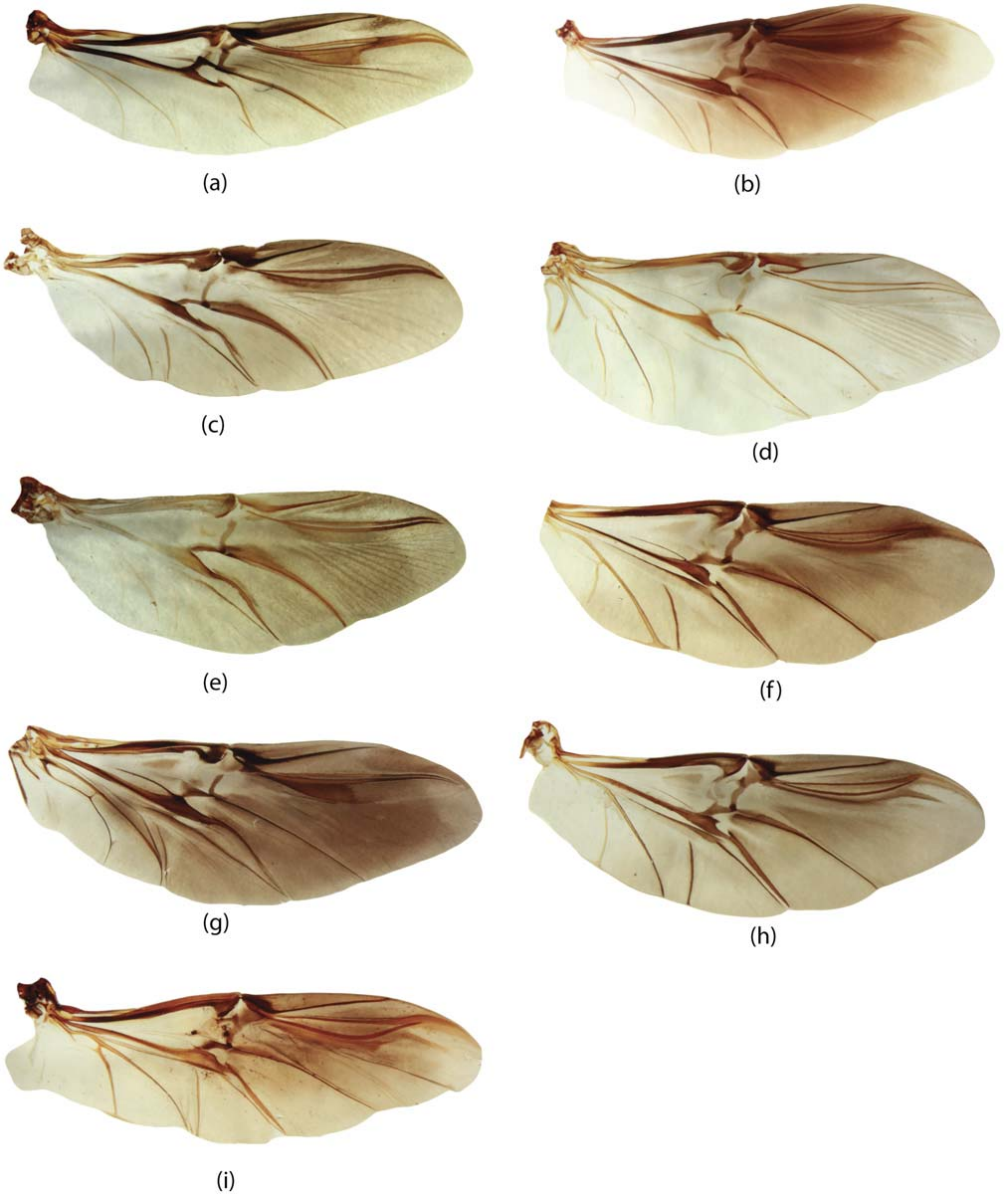
Wings of Scarabaeinae. a, *Neosisyphus sp*.; b, *Sarophorus costatus*; c, *Pycnopanelus krikkeni*; d, *Pedaria sp*.; e, *Hammondantus psammophilus*; f, *Frankenbergerius armatus*; g, *Epirinus sp*.; h, *Delopleurus sp*.; i, *Coptorhina auspicata*; a-i, wings. Wing venation annotated in Fig. 41a, b.

**Fig 39:**
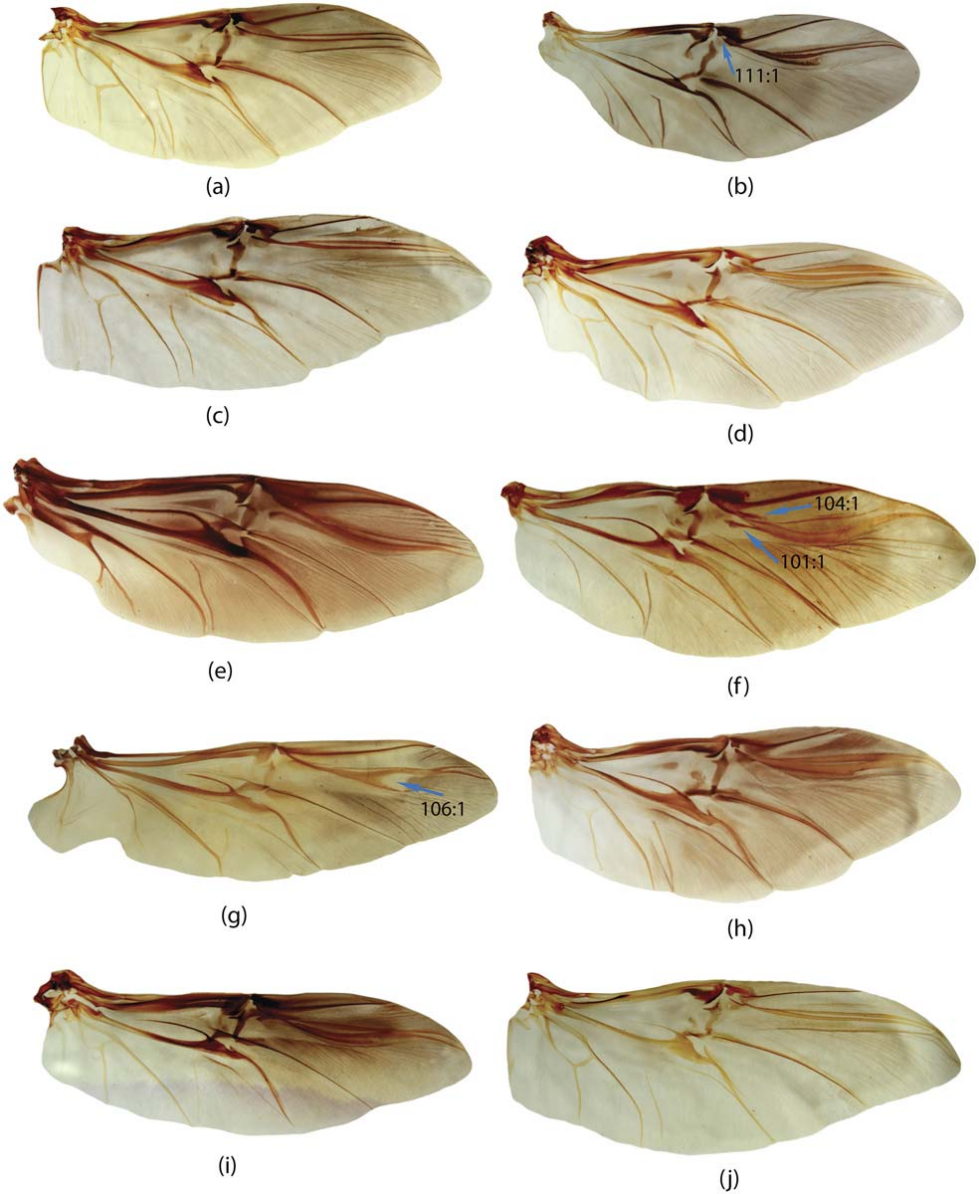
Wings of Scarabaeinae. a, *Coptodactyla nitida*; b, *Ochicanthon sp*.; c, *Metacatharsius sp*.; d, *Dichotomius sericeus*; e, *Megathoposoma candezei*; f, *Canthon virens*; g, *Gymnopleurus leei*; h, *Canthidium bokermanni*; i, *Gromphas aeruginosa*; j, *Ontherus sulcator*; a-j, wings. Wing venation annotated in Fig. 41a, b.

**Fig 40:**
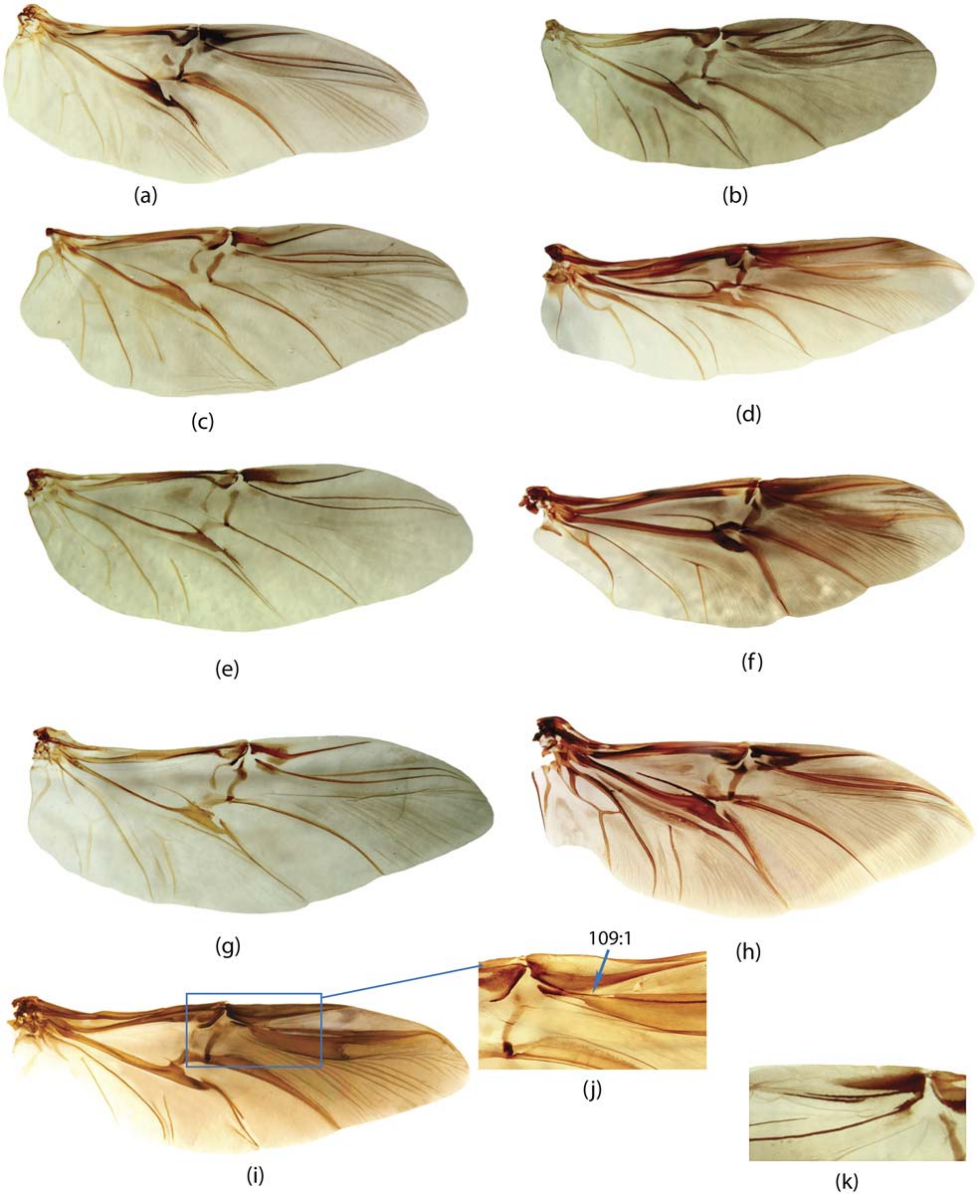
Wings of Scarabaeinae. a, *Xinidium dentilabris*; b, *Sinapisoma sp*.; c, *Scatimus strandi*; d, *Oruscatus davus*; e, *Zonocopris gibbicollis*; f, *Eurysternus hamaticollis*; g, *Bdelyrus seminudus*; h, *Homocopris torulosus*; i, *Onthophagus sp*.; j, *Onthophagus sp*.; k, *Trichillum pauliani*; a-h, wings. Wing venation annotated in Fig. 41a, b.

**Fig 41:**
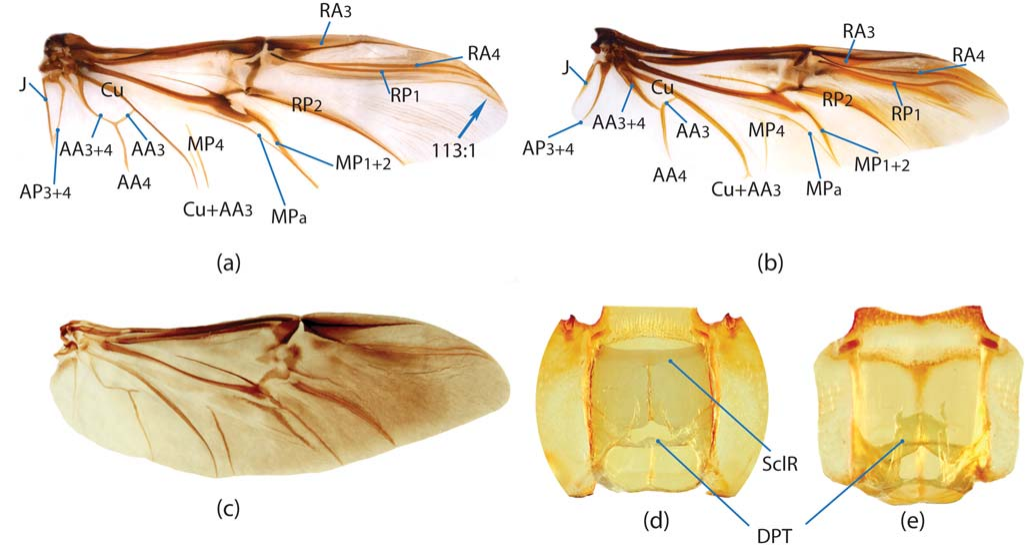
Wings and gula of Scarabaeinae. a, *Copris sp*.; b, *Onitis sp*.; c, *Podotenus fulviventris*; d, *Xinidium dentilabris*; e, *Sarophorus costatus*; a-c, wings; d,e, gula dorsal view.

**Fig 42:**
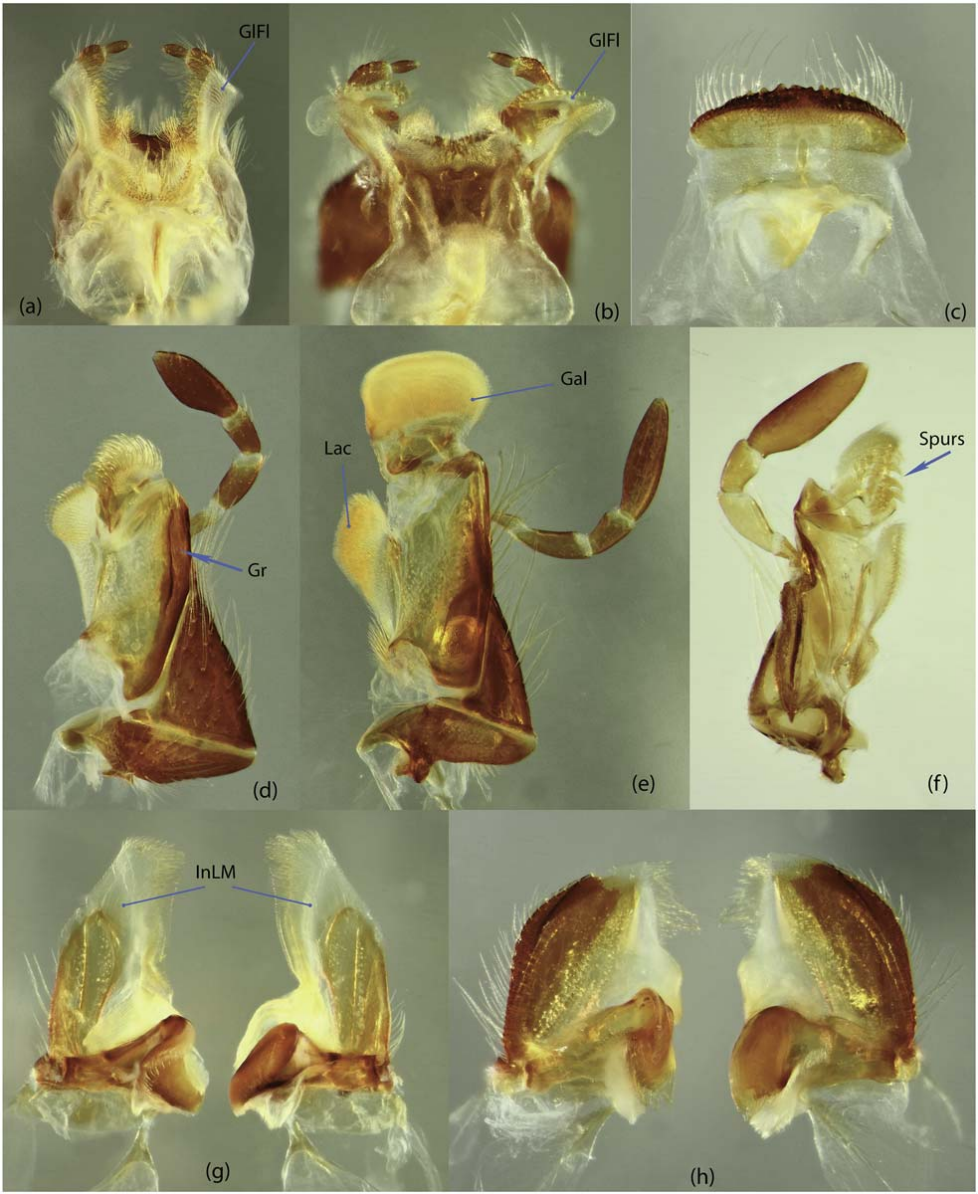
Mouthparts of Scarabaeinae. a, e, g, *Macroderes mutilans*; b-d, h, *Byrrhidium namaquensis*; f, *Delopleurus sp*.; a-b, mentum-glossae, dorsal view; c, epipharynx, dorsal view; d-f, left mandible, ventral view; g-h, mandibles, ventral view.

**Fig 43:**
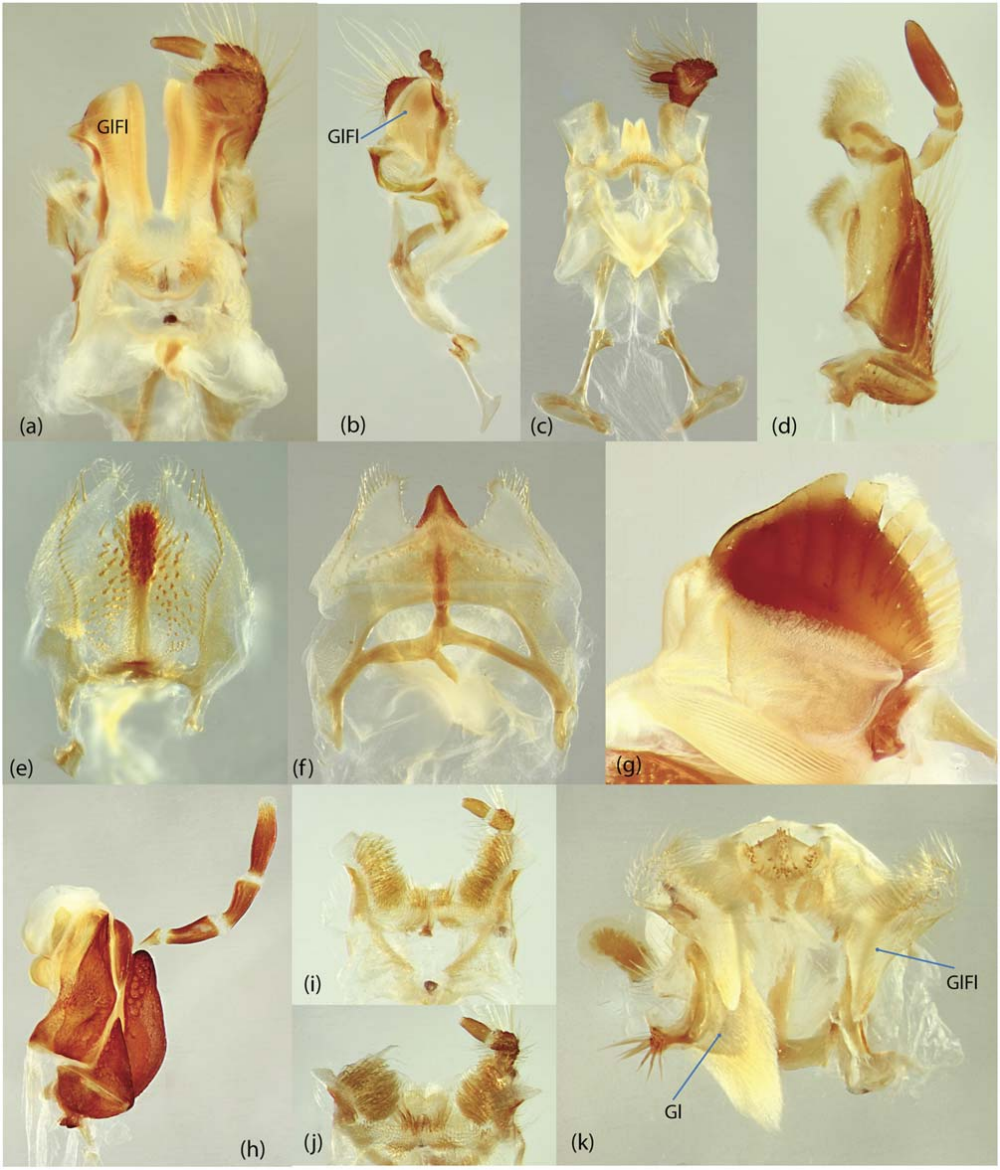
Mouthparts of Scarabaeinae. a,b,e, *Chalcocopris hesperus*; c,f, *Glyphoderus monticola*; d, *Scatonomus fasciculatus*; g,h, *Eucranium arachnoides*; i,j, *Ateuchus squalidus*; k, *Megathoposoma candezei*; a,c,i, hypopharynx and glossae, dorsal view, left maxillary palpus removed; b, hypopharynx and glossae, left lateral view; d,h, left maxilla, ventral view; e, epipharynx, ventral view; f, epipharynx, dorsal view; g, molar lobe of right mandible; j, hypopharynx and glossae, fronto-dorsal view, left maxillary palpus removed; k, hypopharynx and glossa, frontal view, both maxillary palpus removed, left glossa also removed.

**Fig 44:**
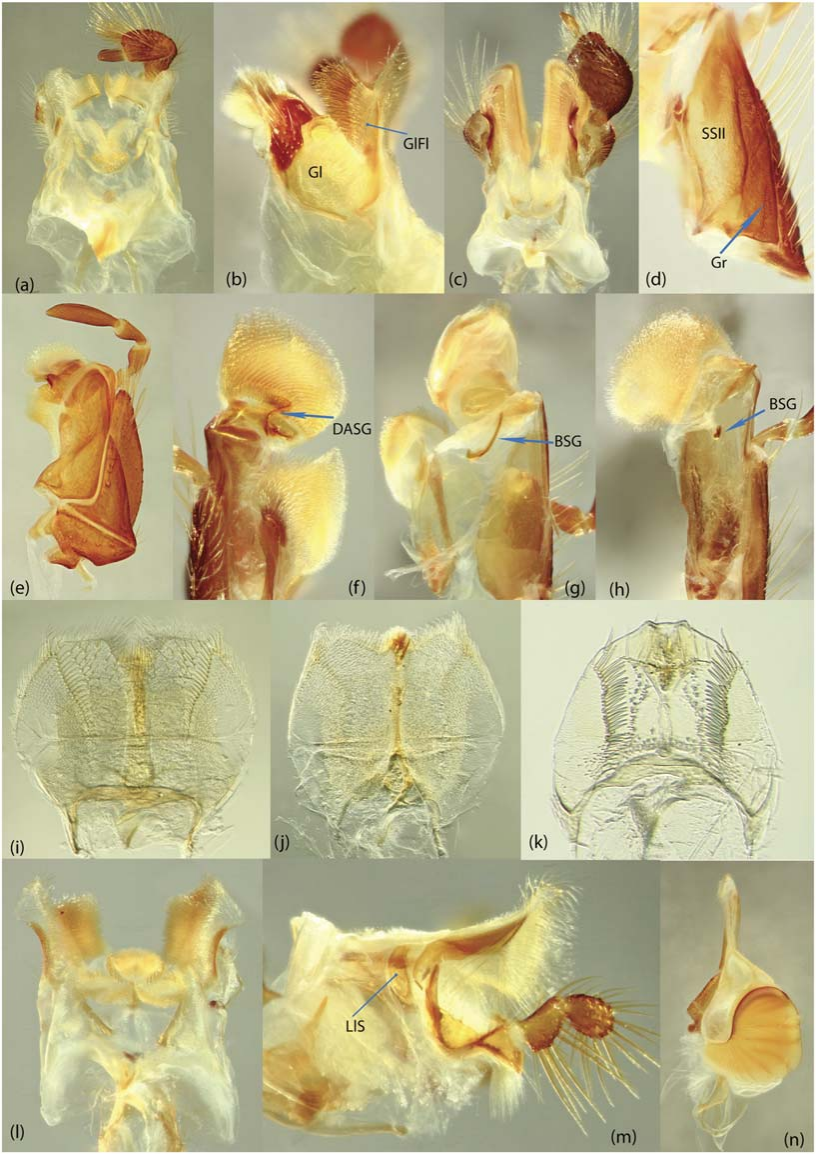
Mouthparts of Scarabaeinae. a,e,n, *Scarabaeus aesculapius*; b, *Anachalcos convexus*; c, *Streblopus opatroides*; d,f, *Chalcocopris hesperus*; h, *Heliocopris sp*; i, *Copris sp*.; j, *Coptodactyla nitida*; k, *Zonocopris gibbicollis*; l, *Metacatharsius sp*.; m, *Eurysternus hamaticollis*; a,c, hypopharynx and glossae, dorsal view, left maxillary palpus removed; b, hypopharynx and glossae, left lateral view, left maxillary palpus removed; d, left maxilla ventral view, stipital sclerite II; f, left maxilla dorsal view, dorsal articular sclerite of galea arrowed; g,h, right maxilla, dorsal view, sclerite of lacinia removed, basal sclerite of galea arrowed; i-k, epipharynx, ventral view; l, hypopharynx and glossae, dorsal view, both maxillary palpus removed; m, hypopharynx and glossae, right lateral view. n, right mandible, left lateral view.

**Fig 45:**
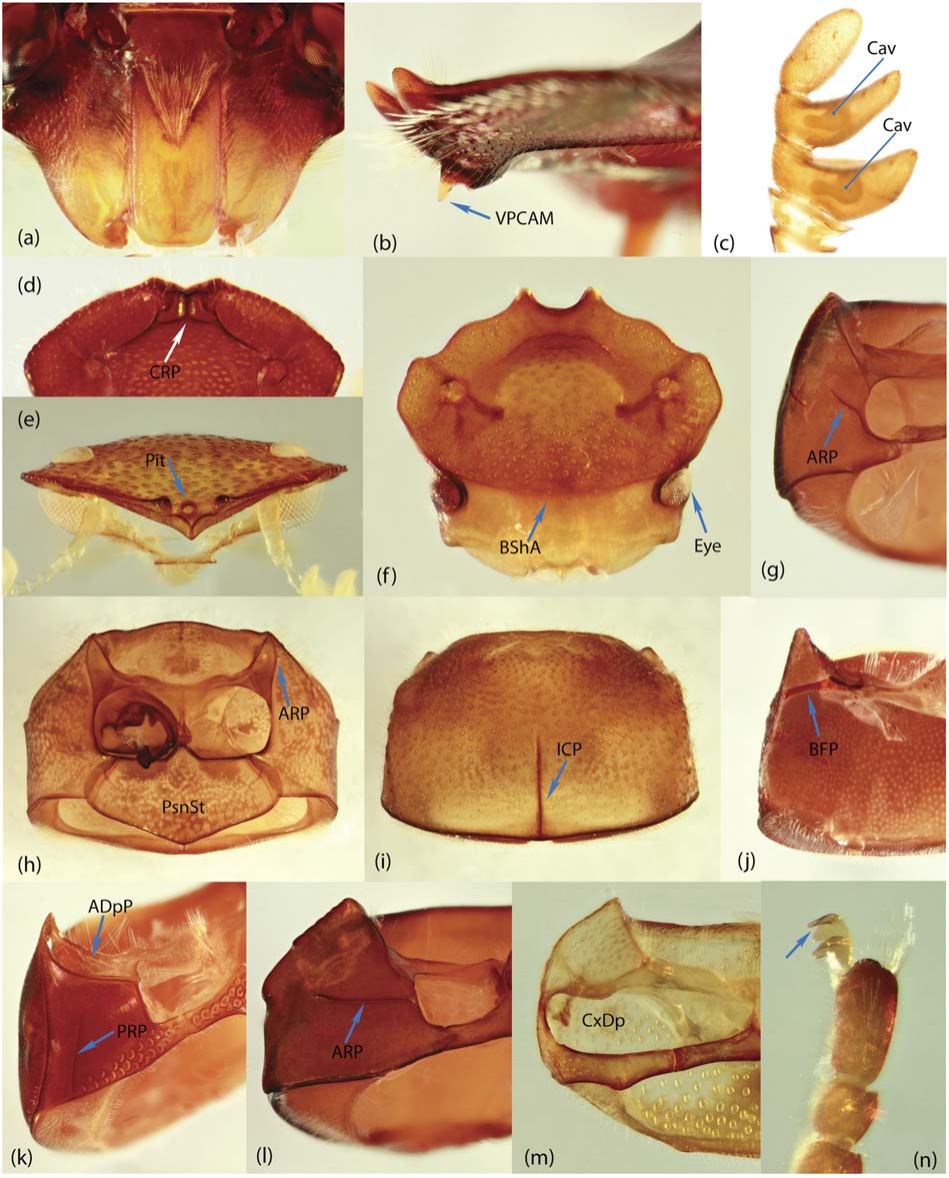
Morphological structures of Scarabaeinae. a, *Aulacopris maximus*; b, *Dichotomius sericeus*; c, *Onthophagus seniculus*; d, *Aphengium sordidum* Harold, 1868; e, *Zonocopris gibbicollis*; f, *Cryptocanthon paradoxus*; g, *Anomiopus edmondsi*; h, *Eurysternus hamaticollis*; i, *Neosisyphus sp*.; j, *Sarophorus costatus*; k, *Coptodactyla nitida*; l, *Hansreia sp*.; m, *Epactoides sp1*.; n, *Pseudonthobium sinuatotibiale*; a, head ventral view; b, head left lateral view; c, antennal club; d, clypeus ventral view; e, head frontal view; f, head dorsal view; g, h, k, l, m, prothorax, ventral view; i, prothorax, dorsal view; j, prothorax, ventral view, propleurae and prosternum removed; n, fore tarsi, bifurcation of claws arrowed.

**Fig 46:**
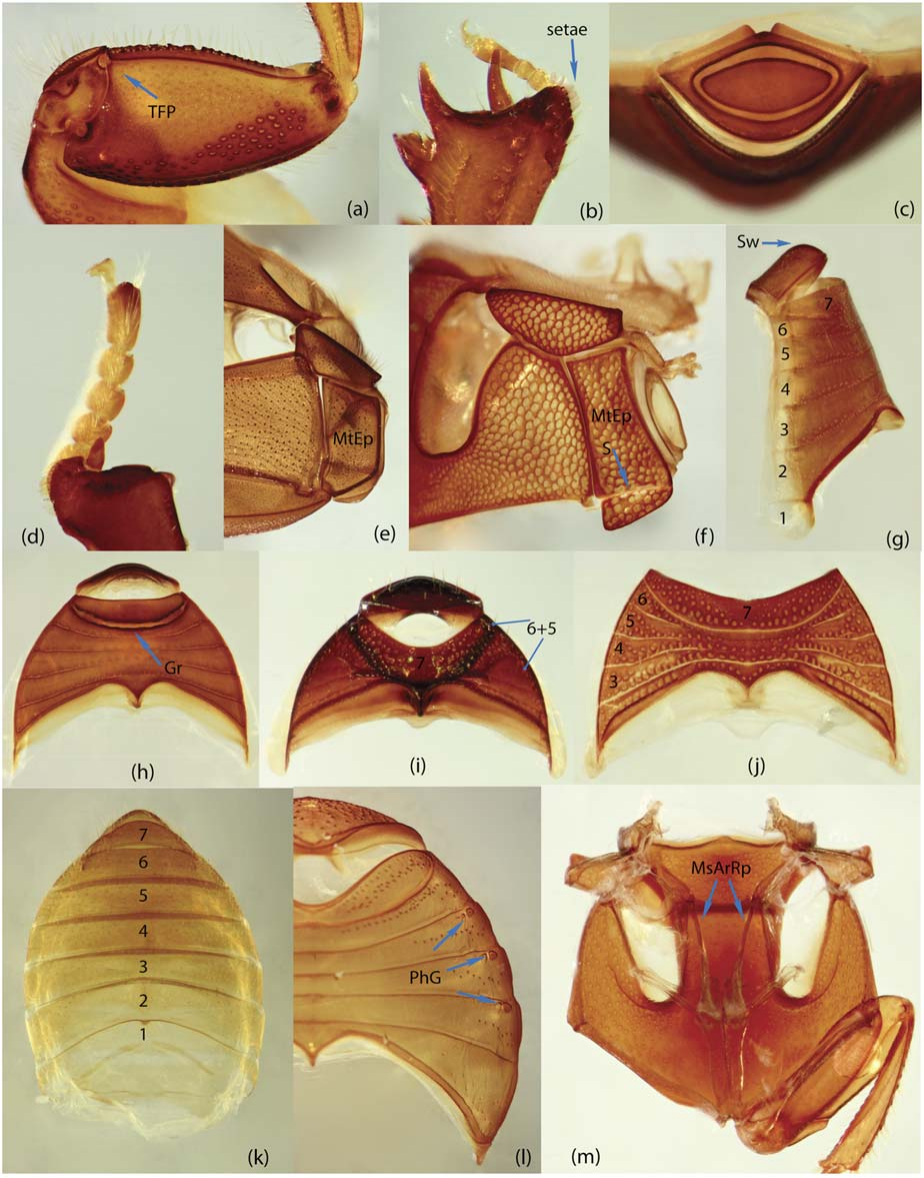
Morphological structures of Scarabaeinae. a, *Coptodactyla glabricollis*; b, *Boletoscapter cornutus*; c, *Bdelyropsis bowditchi*; d, *Pseudonthobium sinuatotibiale*; e, *Paragymnopleurus sp*.; f, *Coptorhina auspicata*; g, *Ochicanthon sp*.; h, *Bdelyropsis bowditchi*; i, *Trichillum pauliani*; j, *Paraphytus sp*.; k, *Podotenus fulviventris*; l, *Pachysoma aesculapius*; m, *Agamopus viridis*; a, femur and trochanter of foreleg; b, apical portion of fore tibia, lamella-like setae arrowed; c, pygidium; d, fore tarsus; e, f, pterothorax, lateral view; g, abdomen, lateral view, sternites enumerated; h-l, abdomen ventral view, sternites partly enumerated; m, internal structure of pterothorax, dorsal view.

**Fig 47:**
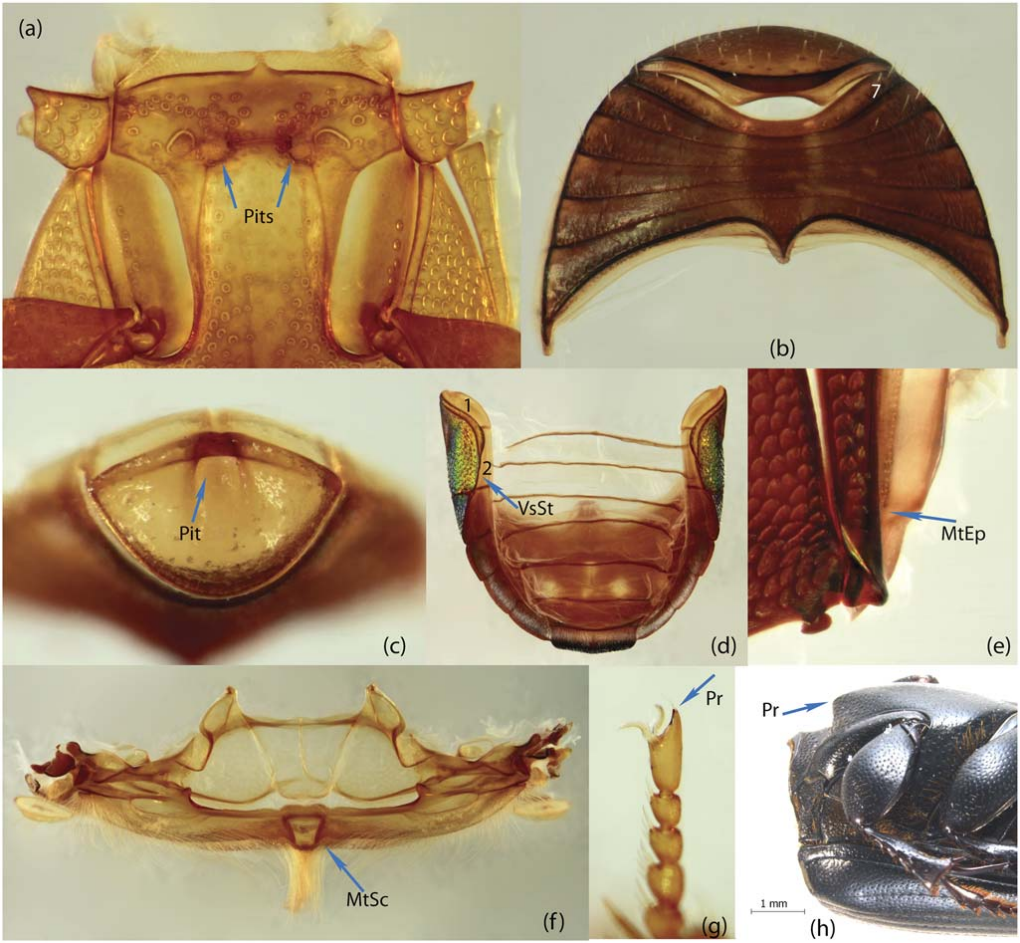
Morphological structures of Scarabaeinae. a, *Zonocopris gibbicollis*; b, *Onthophagus vinctus* Erichson, 1843; c, *Endroedyolus paradoxus*; d, *Gymnopleurus leei*; e, *Aphengium cupreum*; f, *Coprophanaeus telamon*; g, *Zonocopris gibbicollis*; h, *Onthophagus muticifrons* Endrödi, 1973 a, pterothorax, ventral view; b, abdomen, ventral view; c, pygidium; d, abdomen, dorsal view; e, apex of metepisternum, lateral view; f, metanotum; g, mesotarsus; h, pterothorax, lateral view.

1. *Phallobase*, *basal area dorsally*: (0) with one tubercle on each side (Figs. 10e-f, 11d, 12h-i); (1) with single median tubercle or swell, sometimes depressed basally (Figs. 10l, 13b-c, 17a-b,e-f, i-l).
2. *Phallobase*, *general shape (dorsal view)*: (0) cylindrical; symmetrical (i.e., sides of phallobase positioned parallel to each other) or sometimes feebly subsymmetrical (e.g., Figs. 10e-g;. 13b-c); (1) asymmetrical; left basal corner of phallobase distinctly skewed toward right corner (Fig. 33a,b).
3. *Phallobase*, *ground plan of ventral part*: (0) ventral portion reduced presenting large phallobase opening starting at apical margin and continually enlarging toward base; sclerotization of ventral portion (if present laterally) subtle, translucent (Fig. 33b); (1) ventral part present, distinctly sclerotized; opening small usually occupying only basal area of phallobase; sometimes opening extends apically but never occupies entire ventral portion whose sclerotization is always distinct (e.g., Fig. 10g).
4. *Phallobase*, *rectangular notch of basal margin*, *presence (rear view)*: (0) absent; (1) present (Fig. 33e, arrowed).
5. *Phallobase widened and curved medially in lateral view*: (0) absent; (1) present (Fig. 20b, c).
6. *Parameres*, *presence of asymmetry*: (0) absent; both parameres symmetrical (Figs. 1 e-g, l, 4 b, c, 8 e, f, 10 b, c, 11 b, c, 13 d, e, 14 f, g, 15 f, 21 b, c, 23 e, f, 24 c, d, g, h,); (1) present; right paramere modified, usually enlarged (rarely reduced) and curved inward or outward; sometimes modification of right paramere subtle in comparison to left one (Figs. 3 h, 8 a, b, i-l, 12 d, e, 13 b, g, h, 15 b, c, 17 b, c, 19 b-d, h, i, 20 d, e, 21 e, f).
7. *Parameres*, *relatively wide*, *rounded apically (Macroderes—Xinidium groundplan)*: (0) absent; (1) present (see also Fig. 33g, h).
8. *Parameres*, *distinctly acute apically (Dicranocara—Byrrhidium ground plan)*: (0) absent; (1) present (see Fig. 10l).
9. *Parameres*, *simple*, *tiny*, *apically acute (Nebulasilvius*, *Endroedyolus*, *Odontoloma ground plan)*: (0) absent; (1) present (Figs. 10e-g, 33c, d).
10. *Parameres asymmetrical; right paramere widely and obtusely rounded; left paramere widely notched preapically (Hammondantus—Pycnopanelus ground plan)*: (0) absent; (1) present (Figs. 22g, h, 28b, d).
11. *Left paramere wide in lateral view*, *rounded apically; right paramere asymmetrical (Aulacopris—Canthonosoma ground plan)*: (0) absent; (1) present (Fig. 17i, j).
12. *Parameres long*, *thin (Catharsius ground plan)*: (0) absent; (1) present (Fig. 17k,l).
13. *Parameres*, *membrane on lower side strongly sclerotized with two notches basally*: (0) absent; (1) present.
14. *Parameres*, *short*, *rounded apically (Trichillum*, *Genieridium cryptops*, *Onoreidium ground plan)*: (0) absent; (1) present (Fig. 32e, f).
15. *Parameres*, *triangular in lateral view*, *relatively short (Ateuchus ground plan)*: (0) absent; (1) present.
16. *Parameres shortened*, *basolateral plate projected*: (0) absent; (1) present.
17. *Parameres long*, *beak-shaped (Onitis*, *Bubas ground plan)*: (0) absent; (1) present.
18. *Parameres extremely reduced*: (0) absent; (1) present (Fig. 17a,b).
19. *Parameres*, *tips curved upward and more or less bent inward*: (0) absent; (1) present (Fig. 24b,c).
20. *Endophallic sclerites located in superior part of the sac (Trichillum*, *Genieridium*, *Onoreidium ground plan)*: (0) absent; (1) present (Fig. 32g).
21. *Axial sclerite more or less aciculiform*, *simple without complex additional lobes*: (0) absent; (1) present.
22. *Axial sclerite presence*: (0) absent; (1) present (Figs. 1 a-d, i-k, m-q, 2 a-c, g, e, h, f, 3 a, b, d, g, 4 a, d-i, d, 5 a-d, h, 6 c-g, i, 7 a-d, 8 c, d, g-h, m, n, 9 a-f, 10 a, d-f, 11 a, d, g, h, k, 12 a, f, g, 13 a, c, f, i, 14 a-e, h, 15 a, d, e, g, 16 a-f, 17 a, d, e, f, g, 18 a-d, 19 a, e, g, f, 20 a-c, f, g, 21 a, d, g, h, 22a-d, 23 a-d, g, h, i, 24 k, l).
*Note*: In *Odontoloma*, aedeagus contains sclerites X_1_ and X_2_ which, based on Scarabaeinae groundplan, correspond to SA and A sclerites. However, we cannot elucidate which of the two X sclerites in *Odontoloma* corresponds to A and which corresponds to SA as shape of these X sclerites significantly differ from those of SA and A sclerites, and position criterion lacks any information. Therefore, based on Scarabaeinae groundplan, we propose that SA and A sclerites are present in *Odontoloma*, although we are unable to provide detail correspondence of sclerites between *Odontoloma* and the remainder Scarabaeinae.
23. *Axial sclerite and aedeagal sac*, *ground plan (Frankenbergerius*, *Coptorhina*, *Paraphytus*, *Dicranocara*, *Endroedyolus ground plan)*: (0) axial sclerite processed in several lobes and occupying basal part of aedeagal sac; basal part of aedeagal sac possess either solely A or A and BSc sclerites (e.g., Figs. 10j,k,m,n,o,p,q, 11a-c, e-h, 12a-b); (1) axial sclerite differently shaped i.e., simple, without multiple lobes, normally cylindrical or axial sclerite absent.
24. *Axial sclerite*, *central spur-shaped lobe extends from median area of surrounding lobe (Endroedyolus*, *Dicranocara and Paraphytus ground plan)*: (0) absent; (1) present (Figs. 10j-k, m-n, o-q, 11a-c).
25. *Axial sclerite*, *central lobe thin and long*, *surrounding lobe notched on right side (Nebulasilvius-Endroedyolus ground plan)*: (0) absent; (1) present (Figs. 10o-q, 11a).
26. *Axial sclerite*, *central lobe short*, *surrounding lobe more or less triangular (Dicranocara-Byrrhidium ground plan)*: (0) absent; (1) present (Fig. 10i-k, m-n).
27. *Axial sclerite*, *occupies most of basal area of sac; structurally complex; consists of central spur-like sclerite surrounded by multiple lobes; SA sclerites absent (Frankenbergerius—Coptorhina ground plan)*: (0) absent; (1) present (Figs. 11e-h, 12a-b).
28. *Axial sclerite*, *inferior part significantly narrower than remaining portion of sclerite (Macroderes—Xinidium groundplan)*: (0) absent; (1) present (Figs. 23e, 32a).
29. *Axial sclerite*, *aciculiform or with few lobes inferiorly joint with BSc (Trichillum*, *Genieridium cryptops*, *Onoreidium ground plan)*: (0) absent; (1) present (Figs. 21b, c 25e 32d, g, h, i).
30. *Axial sclerite*, *bi- or trifurcated*: (0) absent; (1) present (e.g., Figs. 24d, 18c).
31. *Axial sclerite*, *has unique elongate shape with multiple surrounding lobes located in different planes (Canthochilum ground plan)*: (0) absent; (1) present (Fig. 17c, d).
32. *Axial sclerite associated with multiple surrounding sclerites (Gymnopleurus ground plan)*: (0) absent; (1) present (Figs. 22c, 26f).
33. *Axial sclerite*, *median portion or tip with sclerotized process extending inferiorly*: (0) absent; (1) present (e.g., Figs. 18f, 20h).
34. *Axial sclerite*, *tip with sclerotized process extending inferiorly*: (0) absent; (1) present (e.g., Figs. 18f,27d).
35. *Axial sclerite*, *Λ-shaped (largely bifurcated inferiorly)*: (0) absent; (1) present (Fig. 13h, 31c).
36. *SA sclerite*: (0) absent (Figs. 1 h, i, j-k, m-n, o-p, q, 2 a-c, g, e, h, f, 3 a, b, d, 4 d, e, f, 8 c, d, 12 b, c, 16 e, 23 d, h, i); (1) present (Figs. 1 a-d, 3 g, 4 a, g-i, d, 5 a-d, h, 6 c-g, i, 7 a-d, 8 g-h, m, n, 9 a-f, 10 a, d-f, 11 a, d, g, h, k, 12 a, f, g, 13 a, c, f, i, 14 a-e, h, 15 a, d, e, g, 16 a-d, f, 17 a, d, e, f, g, 18 a-d, 19 a, e, g, f, 20 a-c, f, g, 21 a, d, g, h, 22a-d, 23 a, b, 24 k, l).
*Note*. See note for character 22.
37. *SA sclerite tightly wrapped within axial sclerite; almost inseparable but recognizable (Anachalcos*, *Gyronotus ground plan)*: (0) absent (either SA and A not wrapped tightly or SA and A sclerites not fused, and easy separable or SA and A sclerites distinctly fused or SA and/or A absent); (1) present (Figs. 12f, 22f).
38. *SA and A sclerites distantly located however interlinked with basal extension of A sclerite (Pedaria*, *Coptodactyla)*: (0) absent; (1) present (Figs. 19a, 26g).
39. *SA sclerite bifurcate (Microcopris*, *Pseudopedaria)*: (0) absent; (1) present (Figs. 25b, 28a).
40. *A and SA joint inferiorly by means of inferior accessory sclerite (IAS*_*1*_ *) (Microcopris*, *Pseudopedaria ground plan)*: (0) absent; (1) present (Figs. 25b, 28a).
41. *SA and A strongly fused (Amphistomus ground plan)*: (0) absent; (1) present (Figs. 13a, 16a).
*Note*: We regard the sclerite SA+A (Figs. 13a, 16a) as a fusion of two sclerites SA and A, primarily basing this assumption on the shape of SA + A sclerite that most likely resembles fusion. The lack of additional evidence (e.g., sutures or intermediate forms) may well indicated that our hypotheses of SA and A fusion is false, and SA + A sclerite indeed represent a reduction of one (either A or SA) and an enlargement of the other sclerite.
42. *SA and A sclerites long*, *occupies at least entire inferior half of sac*: (0) absent; (1) present (Figs. 17n, 24a, 26e, 28g).
43. *SA and A sclerites fused together (Epactoides ground plan)*
  (0) absent; (1) present (Fig. 20d, g).
44. FLP*, *presence*: (0) absent; (1) present (Figs. 11e-h, 12a-b).
*Note*: Position of FLP* in aedeagus is similar to that of FLP what can be regarded as an evidence of homology for these two sclerites; however shape of FLP* and FLP differs significantly evidencing against the homology. Thus, in order to avoid *a priori* homology statement, we consider FLP* and FLP to be separate structures.
45. FLP*, *large*, *roughly c-shaped*: (0) absent; (1) present (Fig. 11g-h, 12a-b).
46. FLP* small, *slightly bent right*: (0) absent; (1) present (Fig. 11e-f).
47. *SRP*, *presence*: (0) absent (e.g., Fig.10a,c,i,h); (1) present (e.g., Figs. 12f,g, 13a).
48. *SRP ring-shaped*: (0) absent (SRP differently shaped: without any trace of ring-shaped structure, or absent); (1) present (e.g., Figs. 13a,g, 12g). or at least ring-shape partly formed by poorly visible membrane (Fig. 12e, f).
*Note*: The shape of SRP in *Heliocopris* resembles an intermediate form between state 0 and 1, and therefore cannot be unequivocally scored with either of those states. Due to this ambiguity, we score this character in *Heliocopris* with a question mark.
49. *SRP semicircular or straight (not ring-shaped)*: (0) absent; (1) present (e.g., Figs. 13f, h, 14a).
*Note*: *Heliocopris* possesses the ambiguous state of SRP (see previous character) that also cannot be unequivocally scored with either state of present character. Due to this ambiguity, we score this character in *Heliocopris* with question mark.
50. *SRP sclerite represents flat lamella located along right side of aedeagal sack; SRP bears small ring structure apically (Epirinus—Neosisyphus ground plan)*: (0) absent; (1) present (Figs. 12g, 30h).
51. *SRP not ringed*, *thin*, *its left tip enlarged (Agamopus ground plan)*: (0) absent; (1) present (Fig. 13f, h).
52. *SRP sclerite located in vertical plane*, *flat*, *ringed on the top*, *and with arm-like process toward rear side (Cryptocanthon—Paracanthon ground plan)*: (0) absent; (1) present (Figs. 19d, 27a, b).
53. *Spiculum gastrale*, *basal sclerites*: (0) absent or very weakly sclerotized; (1) distinctly present (Fig. 33f).
54. *Spiculum gastrale*, *basal margin with narrow process apically (Dicranocara-Byrrhidium ground plan)*: (0) absent; (1) present (Fig. 33i).
55. *Spiculum gastrale*, *apical margin widely rounded; basal margin with triangular process (Coptodactyla—Demarziella ground plan)*: (0) absent; (1) present (Fig. 33j).
56. *Spiculum gastrale*, *medial portion narrow*, *straight; basal process long (Catharsius ground plan)*: (0) absent; (1) present.
57. *FLP sclerite(s)*, *presence*: (0) absent (e.g., Fig. 18b, 14c); (1) present (e.g., Figs. 25c, 26g).
*Note*: Judging upon positional criterion *Gymnopleurus* and *Paragymnopleurus* have a suspect of FLP sclerite. However, close association of this FLP suspect with A+SA sclerite indicate that the FLP may in fact represent an additional sclerite (AS). To avoid incorrect homology assumption, this character is coded with question mark in *Gymnopleurus* and *Paragymnopleurus*.
58. *FLP sclerite*, *small c-shaped*, *superior apical portion indistinctly sclerotized (Dichotomius groundplan)*: (0) absent; (1) present (Figs. 18a 19f,g).
59. *FLP sclerite*, *located in front of SA and A sclerites and composed of three distinct lobes*: (0) absent; (1) present.
60. *FLP sclerite*, *big*, *long*, *comprising 2–3 distinct lobes located in the same plane*: (0) absent; (1) present
61. *BSc sclerite*, *semicircularly shaped*, *presence*: (0) absent; (1) present (e.g., Fig. 10a-b, j, m, q).
62. *LC large, ring-shaped in horizontal plane (see direction in Fig. 15b), (Dichotomius ground plan):* (0) absent; (1) present (Figs. 18a 19f,g).
63. *LC well developed and composed of superior and inferior lobes*: (0) absent; (1) present.
64. *IAS*_*2*_ *sclerite*, *presence (Microcopris*, *Pseudopedaria)*: (0) absent; (1) present (Figs. 25b, 28a).
65. *Elytron with 7 distinctly visible striae*: (0) absent; (1) present (Fig. 35m).
*Note*: This and the next three characters account for the number of clearly visible elytral striae. Often, in addition to the visible striae, elytron possesses some traces of striation. The number of striae along with the traces of striation is coded in separate characters #69–72.
66. *Elytron with 8 distinctly visible striae*: (0) absent; (1) present (Figs. 34j, n, i, 35i, j, l, o, q, r, 36a-d, f, g, m, o, 37h, i).
67. *Elytron with 9 distinctly visible striae*: (0) absent; (1) present (Fig. 34a, b, c, d, i, k, l, m, r, 35b, k, n, p, r, 36e, h, i, j, k, l, q, 37a, c, e, f, g).
68. *Elytron with 10 distinctly visible striae*: (0) absent; (1) present (Fig. 34e-h, o, p, q, 35a, c-h, 36k, n, p, r 37b, d).
69. *Elytron with 7 stria and/or traces of striation*: (0) absent; (1) present (Fig. 35m).
70. *Elytron with 8 stria and/or traces of striation 8*: (0) absent; (1) present (Figs. 34l, 35n).
71. *Elytron with 9 stria and/or traces of striation*: (0) absent; (1) present (Fig. 35g, i).
72. *Elytron with 10 stria and/or traces of striation*: (0) absent; (1) present (Fig. 36l).
73. *Elytron*, *1* ^*st*^ *elytral carina*: (0) absent; (1) present (e.g., Fig. 35a, b, o).
74. *Elytron*, *internal margin of 1* ^*st*^ *elytral carina adjoins 7* ^*th*^ *stria*: (0) absent; (1) present (e.g., Fig. 35 b).
75. *Elytron*, *internal margin of 1* ^*st*^ *elytral carina adjoins 8* ^*th*^ *stria*: (0) absent; (1) present (e.g., Fig. 35o).
76. *Elytron*, *2* ^*nd*^ *elytral carina located on pseudoepipleuron*: (0) absent; (1) present (Figs. 34k, l, 35g, i).
77. *Elytron*, *internal margin of 2* ^*nd*^ *elytral carina on pseudoepipleuron adjoins 8* ^*th*^ *stria*: (0) absent; (1) present (Fig. 35i).
78. *Elytron*, *internal margin of 2* ^*nd*^ *elytral carina on pseudoepipleuron adjoins 9* ^*th*^ *stria*: (0) absent; (1) present (Fig. 34k, l).
79. *Elytron*, *internal margin of 2* ^*nd*^ *elytral carina on pseudoepipleuron adjoins 10* ^*th*^ *stria*: (0) absent; (1) present (Fig. 35g).
80. *Elytron*, *2* ^*nd*^ *elytral not located on non-pseudoepipleuron; its internal margin adjoins 8* ^*th*^ *stria ()*: (0) absent; (1) present (Fig. 37g).
81. *Elytron*, *1* ^*st*^ *elytral carina*, *number of ridges*: (0) one (e.g., Fig. 35j, l); (1) two (e.g., Figs. 34n, 35m, 37e).
82. *Elytron*, *8* ^*th*^ *stria largely reduced (Anomiopus—Scatonomus)*: (0) absent; (1) present (Fig. 36m).
83. *Elytron*, *9* ^*th*^ *stria closely approaches 10* ^*th*^ *stria preapically*, *and diverges from 10* ^*th*^ *stria basally*: (0) absent; (1) present (Fig. 36k, n).
84. *Elytron*, *lateral margin subrectangularly protruded preapically*: (0) absent; (1) present (Fig. 36n).
85. *Elytron notched laterally by protrusion of metepisternum (Frankenbergerius—Delopleurus)*: (0) absent; (1) present (Fig. 34e-g).
86. *Elytron*, *last stria (9* ^*th*^ *or 8* ^*th*^ *) visible only preapically*: (0) absent; (1) present (Fig. 34i, j).
*Note*: We consider 8^th^ stria in *Neosisyphus* and 9^th^ in *Epirinus* to be homologous according to the criterion of position. Present character reflects the degree of development of this stria.
87. *Epipleuron*, *size*: (0) relatively narrow; (1) large (Fig. 34c, d).
88. *Elytron*, *7* ^*th*^ *and 8* ^*th*^ *striae largely reduced and visible only preapically*: (0) absent; (1) present (Fig. 34b).
89. *Elytron*, *8* ^*th*^ *and 9* ^*th*^ *striae fused but trace of interstria interval visible*: (0) absent; (1) present (e.g., Figs. 35r, 36e).
90. *Epipleuron*, *slightly protruded downward submedially*: (0) absent; (1) present (e.g., Fig. 36a-c).
91. *Elytron*, *8* ^*th*^ *or 8* ^*th*^ *+9* ^*th*^ *stria(ae) distinctly depressed*: (0) absent; (1) present (e.g., Fig. 36a, d).
92. *Elytron*, *8* ^*th*^ *stria extends from apex to median portion of elytra*, *and very closely located to the 9* ^*th*^ *stria extending all way from apex to base*: (0) absent; (1) present (Fig. 36e, h, i, j).
93. *Elytron*, *out of two last striae (8* ^*th*^ *and 9* ^*th*^ *or 9* ^*th*^ *and 10* ^*th*^ *)*, *inner one extends from apex to median portion of elytra*, *and closely located to (merged with) outer one extending from apex to base*: (0) absent; (1) present (Fig. 36e, h, i, j, p).
*Note*: Here, we consider that 9^th^ and 10^th^ striae in *Copris* are homologous to 8^th^ and 9^th^ striae in all other taxa which are scored with the state one of present character. This statement is based on the positional similarity of two last striae, whereas differences in serial numbers of the striae, in our view, point to the reduction of some preceding striae.
94. *Elytron*, *8* ^*th*^ *stria developed only in medial portion of elytron*: (0) absent; (1) present (Fig. 34m).
95. *Elytron*, *notched laterally by protrusion of abdominal tergites*: (0) absent; (1) present.
96. *Wing*, *RA*_*4*_ *significantly longer than RP*_*1*_ *; RP*_*1*_ *arcuate*, *not parallel to RA*_*4*_: (0) absent; (1) present (Fig. 38b,f,h,j).
97. *Wing*, *MPa largely extends toward junction of Cu and MP*_*1+2*_, *and consists of two distinct halves; of them anterior significantly wider than posterior one*: (0) absent; (1) present (Fig. 38b,f,h,j).
98. *Wing*, *AA*_*4*_ *widened along posterior margin*: (0) absent; (1) present (Fig. 38h,j).
99. *Wing*, *Cu widened along posterior margin*: (0) absent; (1) present (Fig. 38b,f).
100. *Wing*, *RP*_*1*_ *with posterior sclerite*: (0) absent; (1) present (Figs. 38a, g, 39b,d,e,f,g,h,i, 40d,f).
101. *Wing*, *RP*_*1*_ *posterior sclerite represents small basal appendix of RP*_*1*_: (0) absent; (1) present (Fig. 39f).
102. *Wing*, *RP*_*1*_ *with wide posterior sclerite*: (0) absent; (1) present (Fig. 38a,g).
103. *Wing*, *RA*_*4*_ *significantly thinner than RP*_*1*_, *arcuate and not parallel to RA*_*4*_ *; RA*_*4*_ *fused basally with RP*_*1*_: (0) absent; (1) present (Fig. 39f).
104. *Wing*, *RP*_*1*_ *with small anterior sclerite basally*: (0) absent; (1) present (Fig. 39f).
105. *Wing*, *posterior sclerite of RP*_*1*_ *separated from RP*_*1*_: (0) absent; (1) present (Fig. 39d,i).
106. *Wing*, *RP*_*1*_ *and its posterior sclerite fused proximally but bifurcate distally*, *distinctly diverging from each other*: (0) absent; (1) present (Fig. 39g).
107. *Wing*, *MP*_*4*_ *curved and fused with Cu*: (0) absent; (1) present (Fig. 39g).
108. *Wing*, *RA*_*4*_ *gradually thinner medially where it fuses with RP*_*1*_: (0) absent; (1) present (Fig. 39g).
109. *Wing*, *RA*_*3*_ *with posterior sclerite extending toward RA*_*4*_ *or even merging with it*: (0) absent; (1) present (Fig. 40j).
110. *Wing*, *RP*_*1*_ *posterior sclerite approaches RP*_*1*_ *basally*, *almost fusing with it (Ochicanthon*, *Epactoides ground plan)*: (0) absent; (1) present (Fig. 39b).
111. *Wing*, *junction of RA*_*3*_ *and RP*_*1*_ *forms round notch (Ochicanthon—Epactoides ground plan)*: (0) absent; (1) present (Fig. 39b).
112. *Wing*, *J vein well developed; AP*_*3+4*_ *reduced*: (0) absent; (1) present (Fig. 39c,j).
113. *Wing*, *apical area bears sclerite located posteriorly of RP*_*1*_: (0) absent; (1) present (Fig. 41a).
114. *Wing*, *posterior sclerite of RA*_*3*_ *extends from median portion of RA*_*3*_ *to its tip*: (0) absent; (1) present (Fig. 40k).
115. *Wing*, *posterior sclerite of RA*_*3*_ *(similar in shape to that in Fig. 40k) extends from base of RA*_*3*_ *to its median part*: (0) absent; (1) present.
116. *Wing*, *MPa vein presence*: (0) either absent or very weakly developed (Fig. 41c); (1) distinctly present (e.g., Fig. 39a-j).
117. *Galea*, *dorsal part covered with big spurs*: (0) absent; (1) present (Fig. 42f).
118. *Galea elongate and covered with long hairs*: (0) absent; (1) present (Fig. 43d).
119. *Galea and lacinia very closely located to each other; galea strongly sclerotized*, *hook-like*: (0) absent (Fig. 42e, see also character 166); (1) present (Fig. 42d).
120. *Galea and lacinia*, *galea strongly sclerotized*, *hook-like*: (0) absent; (1) present (Fig. 44e).
*Note*: The strongly sclerotized, hook-like structure of galea and lacinia described in this character is similar to that described in character #118. However, we do not consider the similarity in species scored with the state one in present and #119 characters to be homology due to the structural differences in galea. Thus, we code these features using two separate characters.
121. *Galea basal sclerite*, *small*, *more or less round*: (0) absent; (1) present (Fig. 44h).
*Note*: Due to the minute size of *Bohepilissus sp* presence or absence as well as shape of galea basal sclerite was unidentifiable. Therefore, we score this and the next character in *Bohepilissus sp*. with”?”.
122. *Galea*, *basal sclerite longitudinally elongate*: (0) absent; (1) present (Fig. 44g).
*Note*: See note section in previous character.
123. *Maxilla*, *stipital sclerite II medially grooved; surface of groove shagreened*: (0) absent; (1) present (Fig. 44d).
124. *Galea*, *dorsal articular sclerite forms longitudinal carina on galea dorsal surface*: (0) absent; (1) present (Fig. 44f).
125. *Maxilla*, *stipital sclerite II*, *groove on anterior outer margin*: (0) absent; (1) present (Fig. 42d).
126. *Maxilla*, *enlarged*, *strongly sclerotized*, *galea and lacinia reduced in size*, *round*: (0) absent; (1) present (Fig. 44h).
127. *Glossal flap Г-shaped*, *bending inward; its tip covered with big blunt spurs*: (0) absent (Fig. 42a); (1) present (Fig. 42b).
128. *Glossal flap thick; internal margin usually densely haired*: (0) absent; (1) present (Figs. 43a,b, 44c).
129. *Glossal flap thick; its tip bears membranous lobe that bent downward*: (0) absent; (1) present (Fig. 43k).
130. *Glossal flap short*, *with membranous lobes bent inward*: (0) absent; (1) present (Fig. 44a).
131. *Glossal flap triangularly enlarged frontally*, *haired*: (0) absent; (1) present (Fig. 44m).
132. *Glossal flap shortened and wide*: (0) absent; (1) present (Fig. 44c).
133. *Glossa*, *internal margin*, *with dense brush of yellow hairs*: (0) absent; (1) present (Fig. 44f).
134. *Glossa*, *internal margin with thick dense wire-like hairs*: (0) absent; (1) present (Fig. 44i,j).
135. *Glossa*, *internal margin with distinct hairs*: (0) absent; (1) present (Fig. 43k).
136. *Glossa considerably enlarged*, *remarkably bigger than glossal flap*: (0) absent; (1) present (Fig. 44b).
137. *Hypopharyngeal suspensorium*, *lateral labial sclerite*: (0) absent; (1) present (Fig. 44m).
138. *Epipharynx*, *anterior third entirely sclerotized; its anterior margin tuberculated*: (0) absent; (1) present (Fig. 42c).
139. *Epipharynx with triangular deep notch anteriorly*: (0) absent; (1) present (Fig. 43e).
140. *Epipharynx anteriorly with large notch bearing large*, *strongly sclerotized tooth medially*: (0) absent; (1) present (Fig. 44f).
141. *Epipharynx*, *anterior margin slightly processed frontally and blunt medially*: (0) absent; (1) present (Fig. 44k).
142. *Epipharynx*, *anterior margin v-shaped*: (0) absent; (1) present (Fig. 44j).
143. *Epipharynx*, *anterior margin blunt*, *with small medial notch; anterior angles slightly rounded*: (0) absent; (1) present (Fig. 44i).
144. *Mandibles*, *incisor lobe*: (0) mandibles with largely extended membranous incisor lobes (Fig. 42g); (1) membranous incisor lobes of mandibles distinctly reduced (Fig. 42h).
145. *Right mandible*, *molar lobe enlarged*, *with big denticles*: (0) absent; (1) present (Fig. 44g).
146. *Right mandible*, *molar lobe enlarged*, *round*: (0) absent; (1) present (Fig. 44n).
147. *Gula*, *ventral surface with longitudinal groove*: (0) absent; (1) present.
148. *Gula*, *emarginated anteriorly; emargination shagreened and/or haired*: (0) absent; (1) present (Fig. 45a).
149. *Gula*, *internal structure*: (0) gular ridges invaginated only posteriorly, forming longitudinal lamella (dorsal process of tentorium) (Fig. 41e); (1) gular ridges invaginated almost along entire area of gula forming a sclerotized roof over gula with dorsal process of tentorium posteriorly (Fig. 41d).
150. *Eye*, *degree of development*: (0) well-developed; (1) significantly reduced
151. *Clypeus*, *ventral anterior area with ridges forming rectangular pattern that medially divided into two parts*: (0) absent; (1) present (Fig. 45d).
152. *Clypeus*, *anterior margin with sharp ventral process*: (0) absent; (1) present (Fig. 45b).
153. *Clypeus*, *dorsal surface with two big fascicle of dense hairs laterally*: (0) absent; (1) present.
154. *Clypeus*, *apical emargination with medial pit*: (0) absent; (1) present (Fig. 45e).
155. *Head*, *posterior portion setting off anterior edges of eyes*, *shielded by pronotum (shielded area recognized by sculpture distinctly different from that of the rest of head); eyes not exposed dorsally*: (0) absent; (1) present (Fig. 45f).
156. *Antennal club*, *cavity on anterior surface of 1* ^*st*^ *and 2* ^*nd*^ *segments*: (0) absent; (1) present (Fig. 45c).
157. *Hypomera*, *anterior ridge stretches toward lateral margin of hypomera*: (0) absent; (1) present (Fig. 45g,l).
*Note*: In *Epactoides sp2* enlarged coxal depressions, hiding hypomeral anterior ridge, do not allow unequivocal identification of state for this character. Hence, this and the next characters in *Epactoides sp2* are coded with “?”.
158. *Hypomera*, *anterior ridge stretches to anterior angles of pronotum*: (0) absent; (1) present (Fig. 45h).
*Note*: See note section in previous character.
159. *Hypomera*, *anterior portion depressed*: (0) absent; (1) present (Fig. 45k).
160. *Hypomera*, *anterior ridge interrupted medially; lateral premarginal area of hypomera with short ridge*: (0) absent; (1) present (Fig. 45g).
161. *Hypomera*, *posterior longitudinal ridge*: (0) absent; (1) present (Fig. 45k).
*Note*: In *Pedaria sp*, posterior hypomeral portion bears depression with sclerotized folds resembling ridge. However, we cannot unequivocally homologize that posterior depression and folds in *Pedaria* with the posterior ridge. To avoid incorrect homology statement we code this character in *Pedaria sp* with “?”.
162. *Pronotum*, *internal surface of basal margin with medial carina*: (0) absent; (1) present (Fig. 45i).
163. *Pronotum*, *basal margin with two depressions medially (or at least with their traces)*: (0) absent; (1) present.
164. *Prothorax*, *basisternal furca*: (0) absent; (1) present (Fig. 45j).
*Note*: Basisternal furca of prothorax is a newly discovered structure awaiting a thorough investigation, due to its potential informativeness for phylogeny and systematics. In the frame of this paper, we provide a tentative pioneering investigation and score only presence/absence of this structure. Tentative investigation revealed that shape and degree of sclerotization of basisternal furca varies across Scarabaeinae taxa possibly representing a source for more phylogenetic characters.
165. *Prosternum*, *sternellum remarkably enlarged*: (0) absent; (1) present(Fig. 45h).
166. *Prosternum*, *coxal depressions widened*, *closely approaching lateral margin of pronotum*: (0) absent; (1) present (Fig. 45m).
167. *Proleg with trochantofemoral pit*: (0) absent; (1) present (Fig. 46a).
168. *Protibia*, *internal apical angle processed and bears lamella-like setae*: (0) absent; (1) present (Fig. 46b).
169. *Protibia*, *apical spur*: (0) absent/invisible; (1) present/visible.
170. *Protarsus in male*: (0) absent; (1) present.
171. *Protarsus in female*:(0) absent; (1) present.
172. *Protarsus*, *last tarsomere triangularly processed apically*: (0) absent; (1) present.
173. *Protarsus*, *claws*: (0) absent; (1) present.
174. *Protarsus*, *claws big*, *bifurcate preapically*: (0) absent; (1) present (Fig. 45n).
175. *Pro-*, *meso-*, *and metatarsus and apex of meso- and metatibia distinctly haired and/or setose*: (0) absent; (1) present.
176. *Pro-*, *meso-*, *and metatarsus and apex of meso- and metatibia*, *ventral surface*, *with short dense hairs*: (0) absent; (1) present (e.g., Fig. 46c).
177. *Meso- and metatarsus*, *claws*: (0) absent; (1) present.
178. *Meso- and metatarsus with one fixed claw*: (0) absent; (1) present.
179. *Metatarsus and mesotarsus with thick tarsomere and big claws*: (0) absent; (1) present.
180. *Meso- and metatarsus*, *last tarsomere triangularly projected apically*: (0) absent; (1) present (Fig. 47g).
181. *Metatibia*, *number of apical spurs*: (0) no visible spurs; (1) one; (2) two.
182. *Mesotibia*, *number of apical spurs*: (0) no visible spurs; (1) one; (2) two.
183. *Metepisternum*, *apex of lateral surface modified*, *convex and/or depressed (sometimes with unclear keel)*: (0) absent; (1) present (Fig. 47e).
184. *Metepisternum widened (lateral view) cut into elytron by notching it*: (0) absent; (1) present (Fig. 46f).
185. *Metepisternum shape (lateral view) more or less rectangular with apical part rounded*: (0) absent; (1) present (Fig. 46e).
186. *Metepisternum*, *lateral surface*, *apical suture*: (0) absent; (1) present (Fig. 46f).
187. *Mesofurcal arm with*: (0) only one lateral process; (1) with two processes—lateral, and rear directed backward (Fig. 46m).
188. *Mesofurcal arm*, *rear process gross*, *reaches metendosternite*: (0) absent; (1) present (Fig. 46m).
189. *Meso-metasternal pits* (Fig. 47a): (0) absent; (1) present.
190. *Metasternum*, *frontal part raised and projected forward*: (0) absent; (1) present (e.g., Fig. 47h).
191. *Mesoscutellum*, *visible from above in undissected specimen*: (0) absent; (1) present.
192. *Metascutellum*, *small*, *truncate*, *with bunch of hairs apically*: (0) absent; (1) present (Fig. 47f).
193. *Abdomen general structure*: (0) abdomen soft; sternites separated from each other by membrane; 1st sternite well developed laterally (Fig. 46k); (1) abdomen strongly sclerotized; sternites tightly attached to each other lacking membranous interconnection; 1st sternite small (Fig. 46g-j).
194. *Abdomen*, *7* ^*th*^ *sternite largely expanded and crowding out remain sternites along midline; remain sternites visible only laterally*: (0) absent; (1) present (Fig. 46i).
195. *Abdomen*, *6* ^*th*^ *and 5* ^*th*^ *sternites almost or entirely fused; border between them almost or entirely invisible*: (0) absent; (1) present (Fig. 46i).
196. *Abdomen*, *7* ^*th*^ *sternite distinctly squeezed along midline in both male and female*: (0) absent; (1) present (e.g., Fig. 47b).
197. *Abdomen*, *7* ^*th*^ *sternite distinctly squeezed along midline only in male while more or less normally developed in female*: (0) absent; (1) present (e.g., Fig. 47b).
198. *Abdomen*, *ventral surface of 2* ^*nd*^ *-4* ^*th*^ *abdominal sternites runs over its dorsal surface*: (0) absent; (1) present (Fig. 47d).
199. *Abdomen*, *ventral surface of 2* ^*nd*^ *-4* ^*th*^ *abdominal sternites runs over its dorsal surface; ventral surface lacks suture between 2* ^*nd*^ *and 3* ^*rd*^ *sternites*: (0) absent; (1) present (Fig. 47d).
200. *Abdomen*, *lateral pheromone glands*: (0) absent; (1) present (Fig. 46l).
201. *Abdomen*, *7* ^*th*^ *sternite grooved basally*: (0) absent; (1) present (Fig. 46h).
202. *Abdomen*, *sternites 3–6 fused along midline*: (0) absent; (1) present (Fig. 46j).
203. *Pygidium distinctly swollen*: (0) absent; (1) present (Fig. 46g).
204. *Pygidium with grooved line(s) and/or ring*: (0) absent; (1) present (Fig. 46c).
205. *Pygidium*, *medial pit*: (0) absent; (1) present (Fig. 47c).

## Supporting Information

S1 Document The output of the script [48] listing unstable taxa/clades and characters. (TXT)

S1 Fig Majority consensus (50%) of MPTs from the analysis #2 (unweighted parsimony). (PDF)

S2 Fig Majority consensus (50%) of MPTs from the analysis #3 (unweighted parsimony). (PDF)

S3 Fig Majority consensus (50%) of MPTs from the analysis #4 (unweighted parsimony). (PDF)

S4 Fig Majority consensus (50%) of MPTs from the analysis #6 (unweighted parsimony). (PDF)

S5 Fig Phylogenetic tree from Bayesian analysis with parameter-partition scheme #9.3 (autapomorphies excluded). Values above branches indicate posterior probabilities.
(PDF)

S6 Fig Phylogenetic tree from Bayesian analysis with parameter-partition scheme #9.3 (autapomorphies included). Values above branches indicate posterior probabilities.
(PDF)

S7 Fig Phylogenetic tree from Bayesian analysis with parameter-partition scheme #8.11 (autapomorphies included). Values above branches indicate posterior probabilities.
(PDF)

S1 Matrix Data matrix of Scarabaeinae taxa used in the phylogenetic analyses. (NEX)

S1 Table Taxa examined in the phylogenetic analyses. (DOCX)

S2 Table Frequency of characters supporting instability. (XLSX)

S3 Table Summary of 164 models tested using Bayesian framework. (XLSX)
