# Supplementary figures and images for: Innovative Bayesian and Parsimony Phylogeny of Dung Beetles (Coleoptera, Scarabaeidae, Scarabaeinae) Enhanced by Ontology-Based Partitioning of Morphological Characters
Source: PLoS One. 2015 Mar 17;10(3):e0116671. doi: 10.1371/journal.pone.0116671 (PMC4363793; doi:10.1371/journal.pone.0116671)

Majority rule tree (from 392 trees, cut 50)

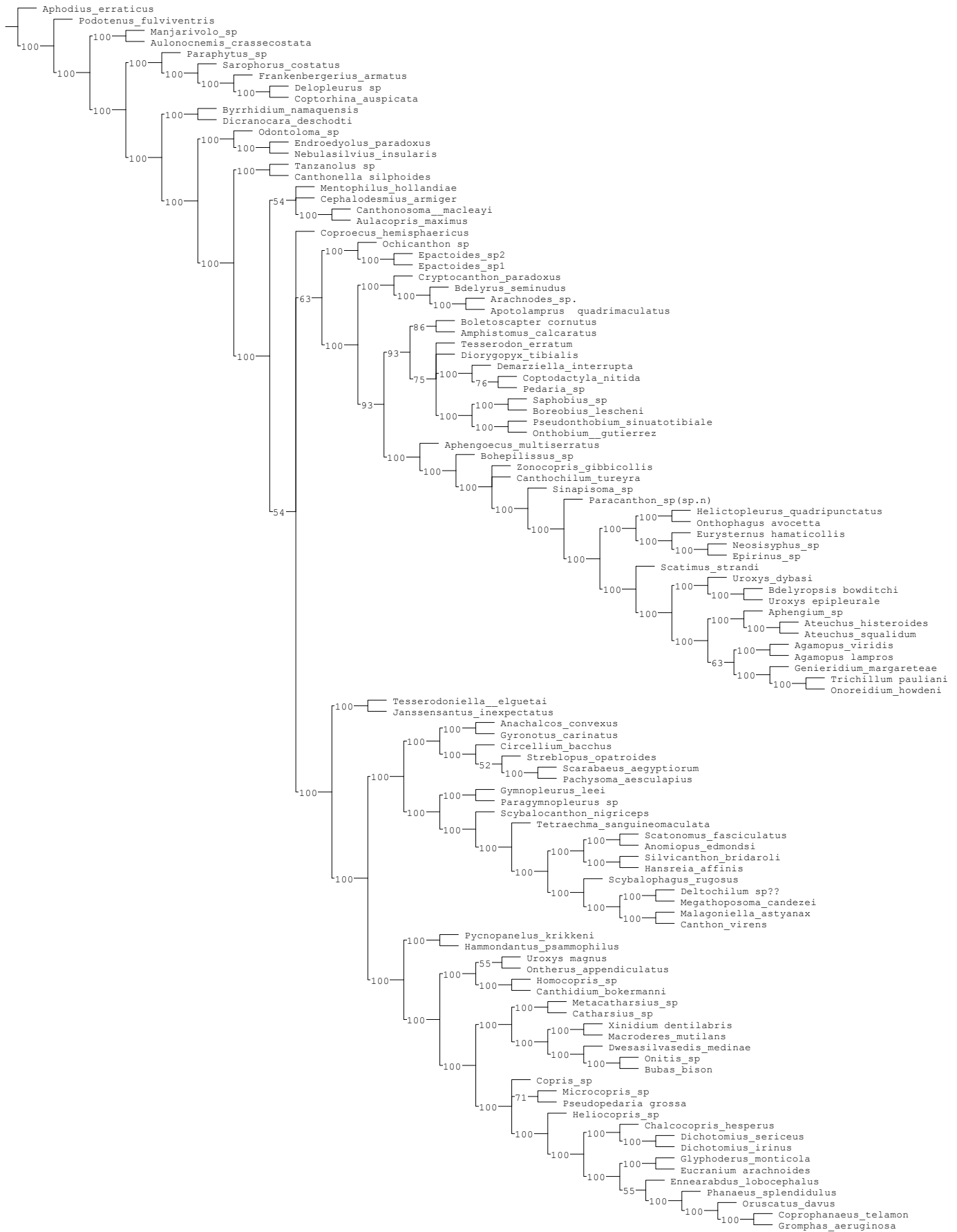

Supplement: S1 Fig — (PDF) [file pone.0116671.s002.pdf]

Majority rule tree (from 11686 trees, cut 50)

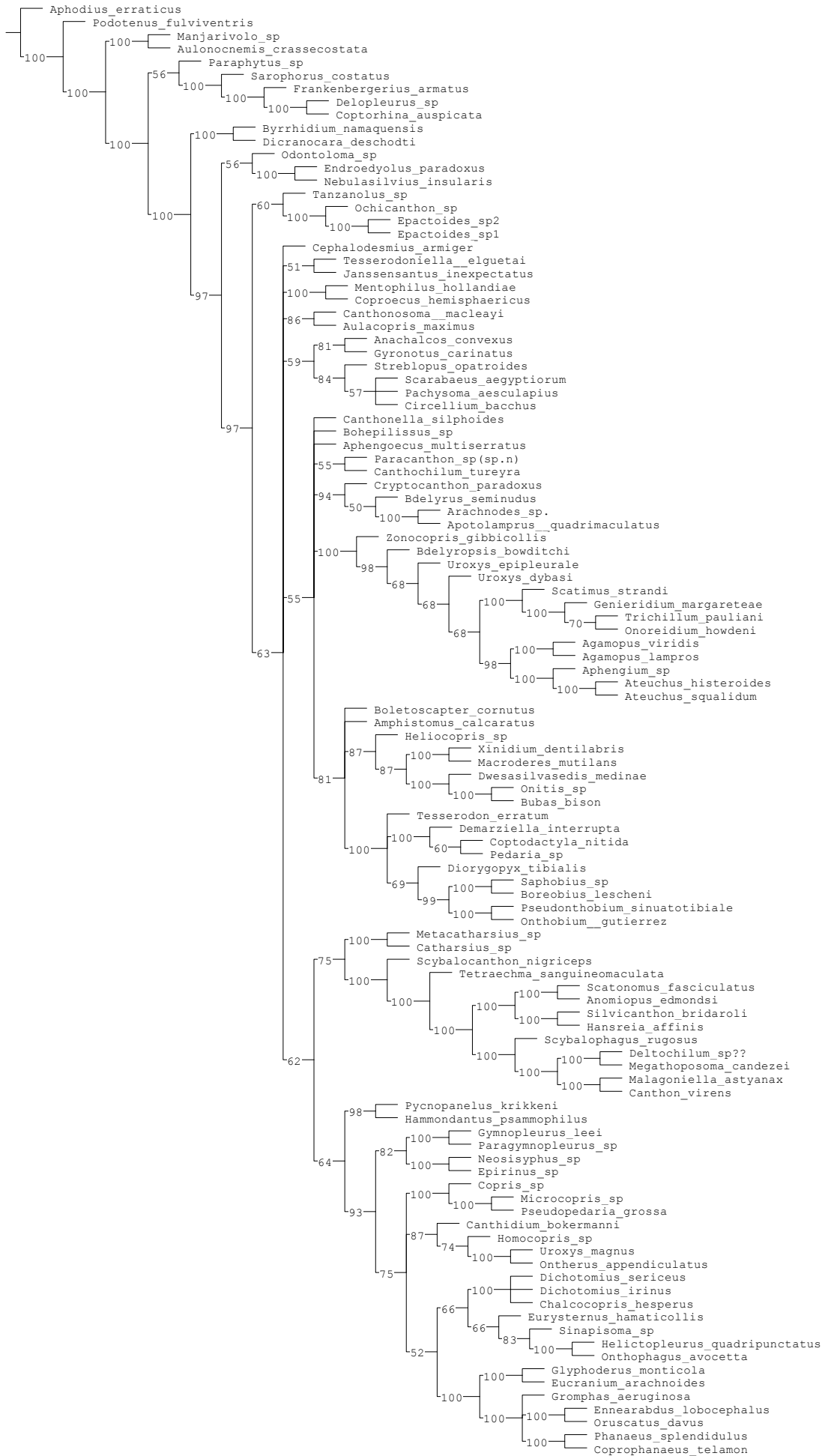

Supplement: S2 Fig — (PDF) [file pone.0116671.s003.pdf]

Majority rule tree (from 11491 trees, cut 50)

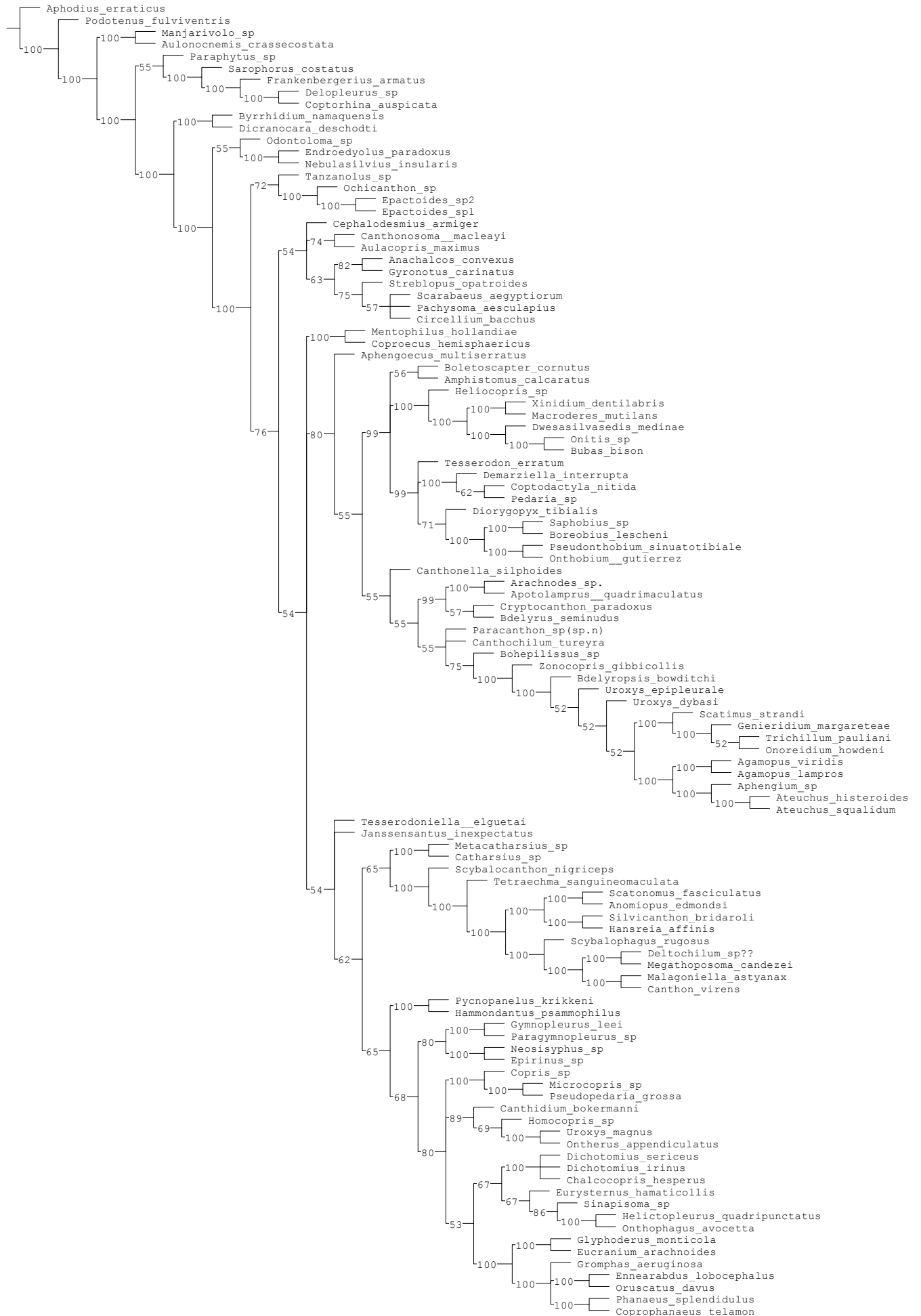

Supplement: S3 Fig — (PDF) [file pone.0116671.s004.pdf]

Majority rule tree (from 164436 trees, cut 50)

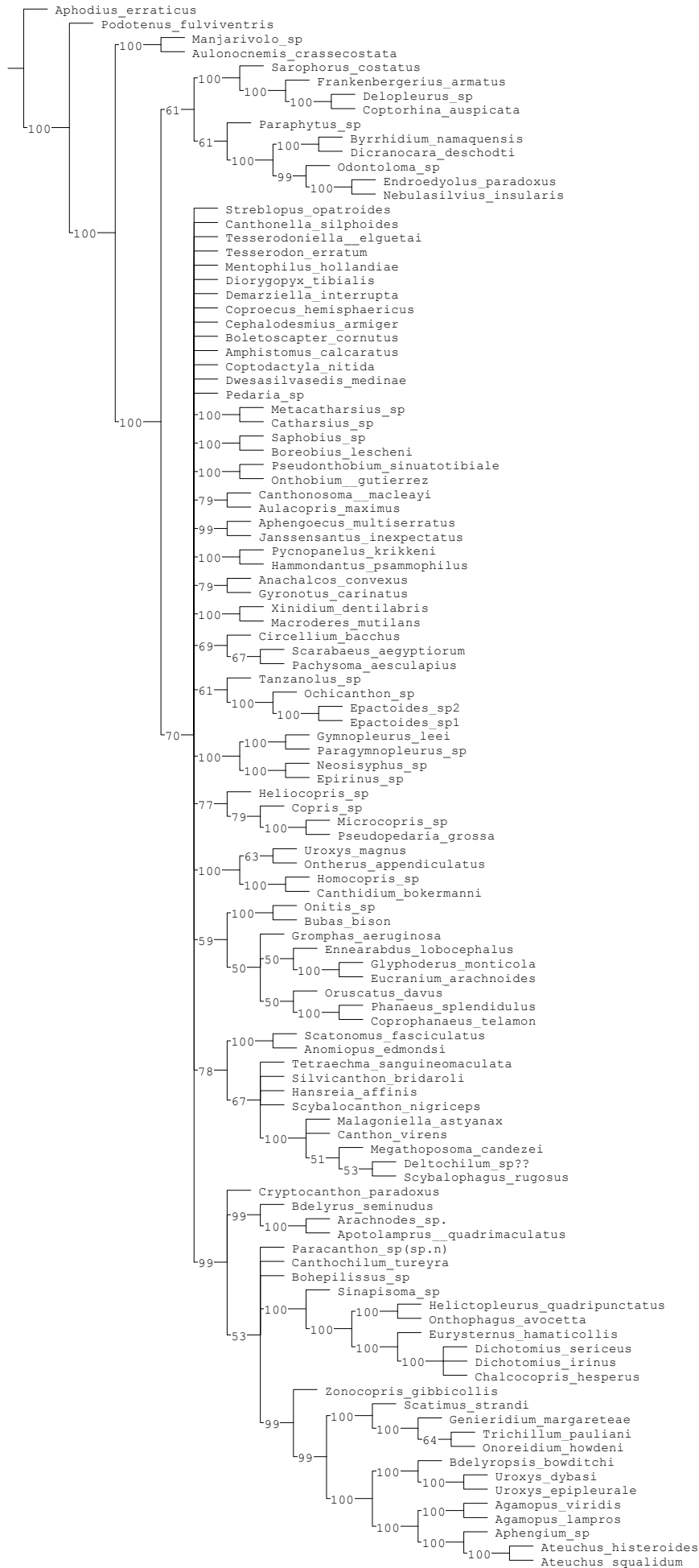

Supplement: S4 Fig — (PDF) [file pone.0116671.s005.pdf]

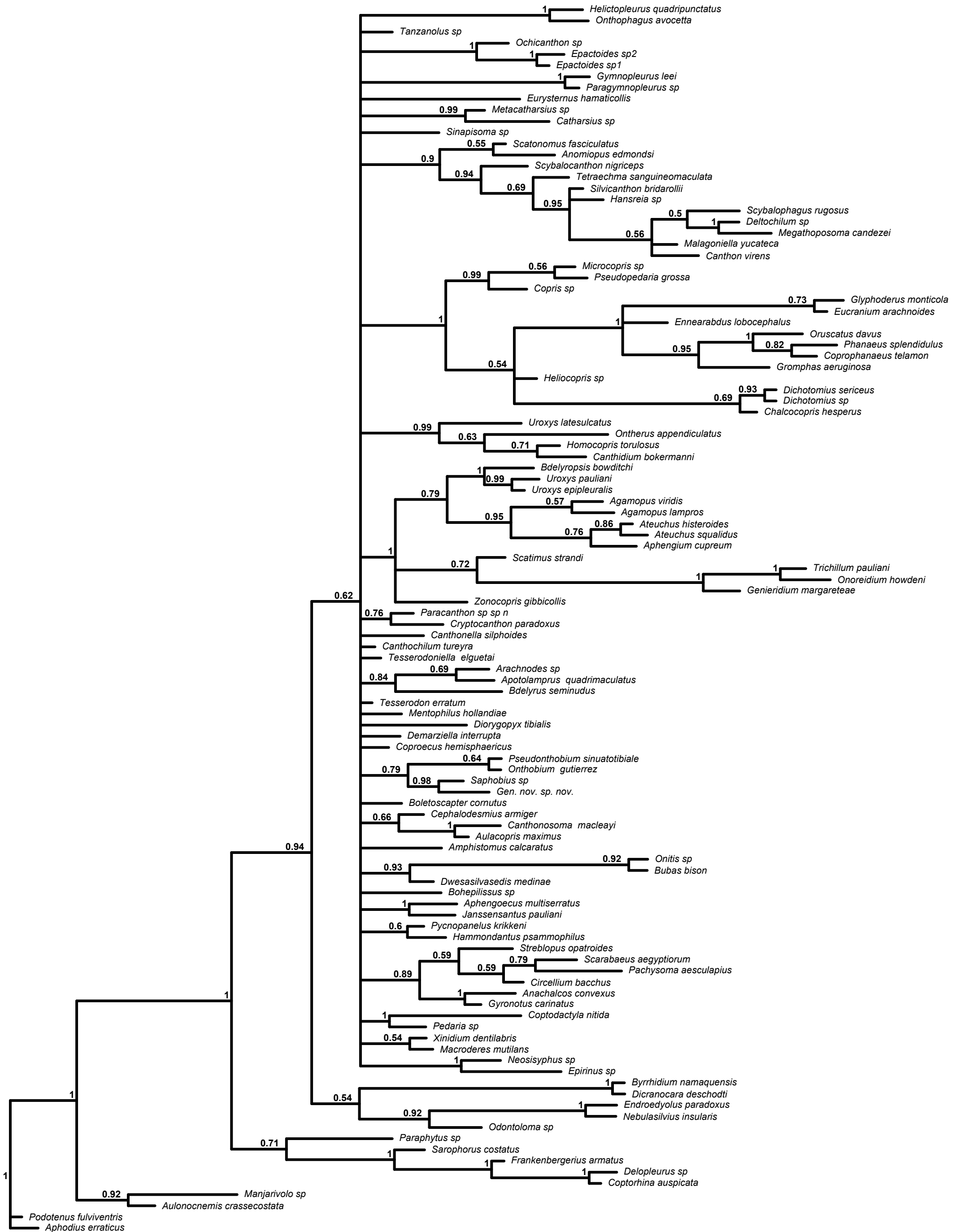

Supplement: S5 Fig — Values above branches indicate posterior probabilities. (PDF) [file pone.0116671.s006.pdf]

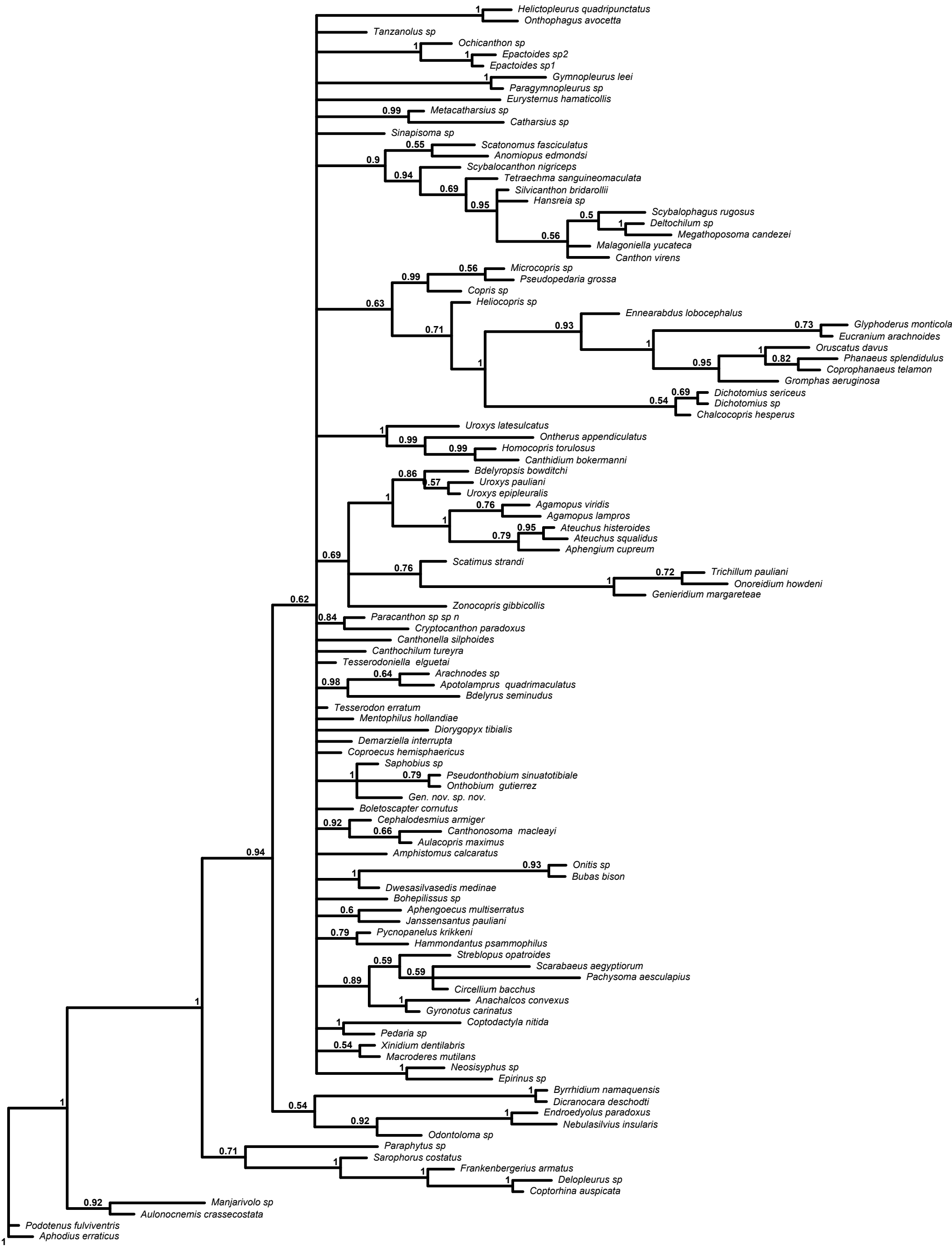

Supplement: S6 Fig — Values above branches indicate posterior probabilities. (PDF) [file pone.0116671.s007.pdf]

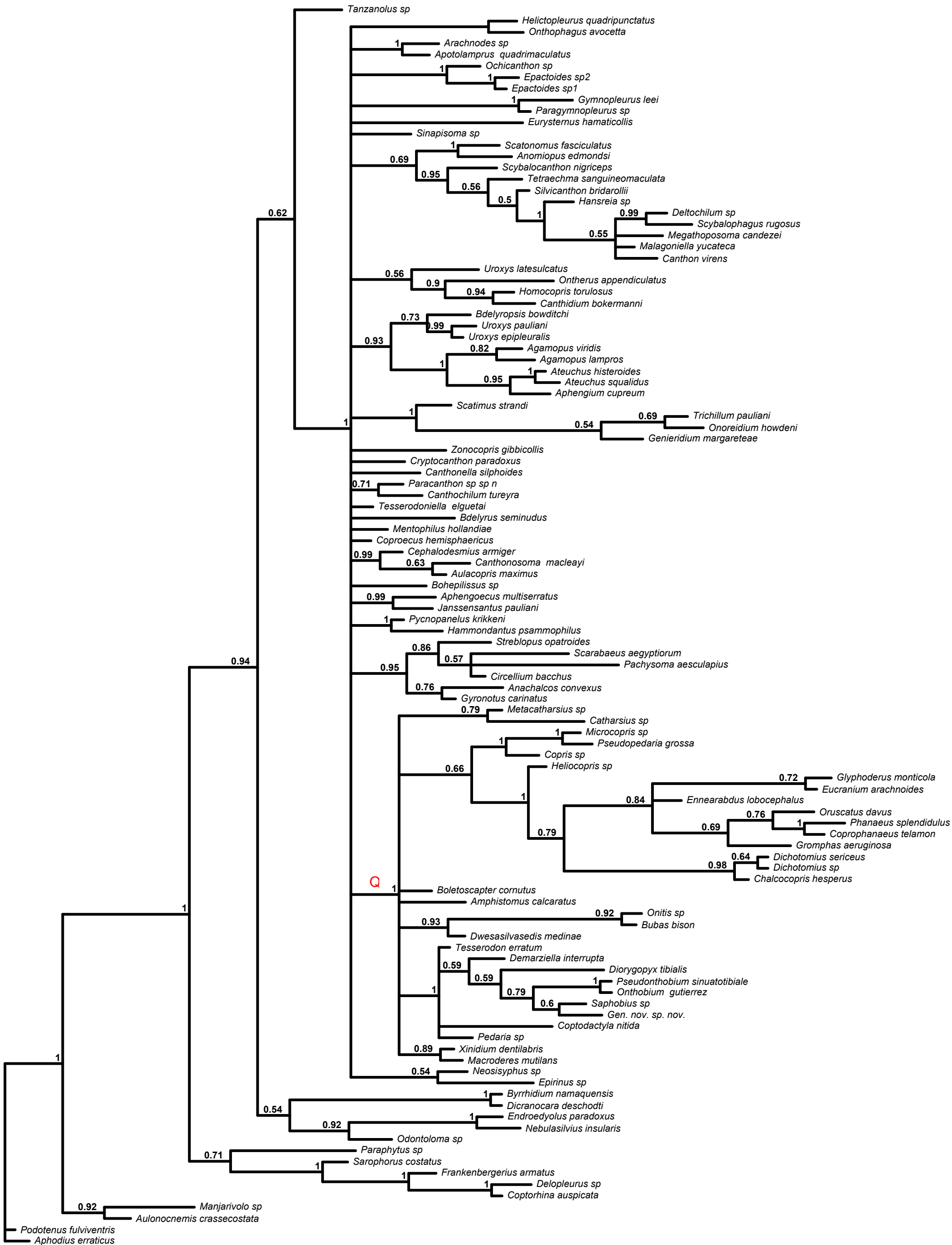

Supplement: S7 Fig — Values above branches indicate posterior probabilities. (PDF) [file pone.0116671.s008.pdf]
