# Supplementary material for: Innovative Bayesian and Parsimony Phylogeny of Dung Beetles (Coleoptera, Scarabaeidae, Scarabaeinae) Enhanced by Ontology-Based Partitioning of Morphological Characters
Source: PLoS One. 2015 Mar 17;10(3):e0116671. doi: 10.1371/journal.pone.0116671 (PMC4363793; doi:10.1371/journal.pone.0116671)
Supplement: S1 Table — (DOCX) [file pone.0116671.s010.docx]

Table S1. Taxa examined in the phylogenetic analyses.

| # | Tribe. | Genus | Species | Author | Year of description | Male | Female | Country | Biogeograpghic Region | Deposition |
| --- | --- | --- | --- | --- | --- | --- | --- | --- | --- | --- |
| 190 | Aphodiini | *Aphodius* | *erraticus* | Linnaeus | 1758 | 2 |  | Russia | Palearctic | cST |
| 197 | Aphodiini | *Podotenus* | *fulviventris* | Fairmaire and Germain | (1860) |  |  | Chile | Neotropical | cST |
| 26 | Aulonocnemini | *Aulonocnemis* | *crassecostata africana* | Boucomont | 1937 | 1 |  | Tanzania | Afrotropical | ZMUC |
| 25 | Aulonocnemini | *Manjarivolo* | *sp* |  |  | 1 |  | Madagascar | Afrotropical | FMNH |
| 170 | Coprini | *Catharsius* | *sp* |  |  | 1 |  | South Africa | Afrotropical | cST |
| 181 | Coprini | *Copris* | *sp* |  |  | 1 |  | Laos | Oriental | cST |
| 82 | Coprini | *Coptodactyla* | *nitida* | Paulian | 1933 | 1 |  | Australia | Australian | CMNC |
| 157 | Coprini | *Metacatharsius* | *sp* |  |  | 1 |  | South Africa | Afrotropical | cST |
| 184 | Coprini | *Microcopris* | *sp* |  |  | 1 |  | Laos | Oriental | cST |
| 154 | Coprini | *Pseudopedaria* | *grossa* | Thomson | (1858) | 1 |  | Ghana | Afrotropical | UPSA |
| 51 | Deltochilini | *Agamopus* | *lampros* | Bates | 1887 | 1 |  | Mexico | Neotropical | cST |
| 94 | Deltochilini | *Agamopus* | *viridis* | Boucomont | 1928 | 1 |  | Brazil | Neotropical | CMNC |
| 105 | Deltochilini | *Amphistomus* | *calcaratus* | MacLeay | (1871) | 2 |  | Australia | Australian | CMNC |
| 172 | Deltochilini | *Anachalcos* | *convexus* | Boheman | 1857 | 1 |  | South Africa | Afrotropical | cST |
| 72 | Deltochilini | *Anomiopus* | *edmondsi* | Canhedo | 2006 | 1 |  | Venezuela | Neotropical | CMNC |
| 174 | Deltochilini | *Aphengoecus* | *multiserratus* | Scholtz and Howden | 1987 | 1 |  | South Africa | Afrotropical | UPSA |
| 219 | Deltochilini | *Apotolamprus* | *quadrimaculatus* | Lebis | 1953 | 1 |  | Madagascar | Afrotropical | MNHN |
| 187 | Deltochilini | *Arachnodes* | *sp* |  |  | 1 | 1 | Madagascar | Afrotropical | CASC |
| 119 | Deltochilini | *Aulacopris* | *maximus* | Matthews | (1974) | 1 |  | Australia | Australian | CMNC |
| 166 | Deltochilini | *Bohepilissus* | *sp* |  |  | 2 |  | South Africa | Afrotropical | cST |
| 111 | Deltochilini | *Boletoscapter* | *cornutus* | MacLeay | (1887) | 2 |  | Australia | Australian | CMNC |
| 85 | Deltochilini | *Gen.nov* | *sp.nov* |  |  | 2 |  | New Zealand | Australian | CMNC |
| 148 | Deltochilini | *Byrrhidium* | *namaquensis* | Scholtz and Howden | 1987 | 1 |  | South Africa | Afrotropical | UPSA |
| 71 | Deltochilini | *Canthochilum* | *tureyra* | Zayas & Matthews | 1966 | 2 |  | Cuba | Neotropical | CMNC |
| 23 | Deltochilini | *Canthon* | *virens* | Mannerheim | 1829 | 1 |  | Brazil | Neotropical | cST |
| 91 | Deltochilini | *Canthonella* | *silphoides* | Harold | (1867) | 1 |  | Colombia | Neotropical | CMNC |
| 49 | Deltochilini | *Canthonosoma* | *macleayi* | Harold | (1868) | 1 |  | Australia | Australian | CMNC |
| 106 | Deltochilini | *Cephalodesmius* | *armiger* | Westwood | 1842 | 2 |  | Australia | Australian | CMNC |
| 202 | Deltochilini | *Circellium* | *bacchus* | Fabricius | (1781) | 1 |  | South Africa | Afrotropical | cST |
| 103 | Deltochilini | *Coproecus* | *hemisphaericus* | Guérin-Méneville | (1844) | 1 |  | Australia | Australian | CMNC |
| 52 | Deltochilini | *Cryptocanthon* | *paradoxus* | Balthasar | 1942 | 1 |  | Peru | Neotropical | cST |
| 142 | Deltochilini | *Dicranocara* | *deschodti* | Frolov and Scholtz | 2003 | 1 |  | Namibia | Afrotropical | UPSA |
| 97 | Deltochilini | *Diorygopyx* | *tibialis* | MacLeay | 1871 | 1 |  | Australia | Australian | CMNC |
| 210 | Deltochilini | *Dwesasilvasedis* | *medinae* | Deschodt & C.H.Scholtz | 2008 | 1 |  | South Africa | Afrotropical | UPSA |
| 209 | Deltochilini | *Endroedyolus* | *paradoxus* | Scholtz and Howden | 1987 | 1 |  | South Africa | Afrotropical | TMSA |
| 29 | Deltochilini | *Epactoides* | *sp1* |  |  | 1 |  | Madagascar | Afrotropical | cST |
| 198 | Deltochilini | *Epactoides* | *sp2* |  |  | 2 |  | Madagascar | Afrotropical | CASC |
| 162 | Deltochilini | *Epirinus* | *sp* |  |  | 2 |  | South Africa | Afrotropical | cST |
| 144 | Deltochilini | *Gyronotus* | *carinatus* | Felsche | 1911 | 1 |  | South Africa | Afrotropical | UPSA |
| 167 | Deltochilini | *Hammondantus* | *psammophilus* | Cambefort | 1978 |  |  | Namibia | Afrotropical | UPSA |
| 109 | Deltochilini | *Hansreia* | *sp* |  |  | 1 |  | French Guiana | Neotropical | CMNC |
| 145 | Deltochilini | *Janssensantus* | *pauliani* | Scholtz & Howden | 1987 | 1 |  | Tanzania | Afrotropical | UPSA |
| 79 | Deltochilini | *Malagoniella* | *yucateca* | Harold | (1863) | 1 |  | Honduras | Neotropical | CMNC |
| 87 | Deltochilini | *Megathoposoma* | *candezei* | Harold | (1873) | 1 |  | Costa Rica | Neotropical | CMNC |
| 118 | Deltochilini | *Mentophilus* | *hollandiae* | Boisduval | (1835) | 1 |  | Australia | Australian | CMNC |
| 153 | Deltochilini | *Nebulasilvius* | *insularis* | Deschodt & Scholtz | 2008 | 1 |  | South Africa | Afrotropical | UPSA |
| 211 | Deltochilini | *Ochicanthon* | *sp* |  |  | 1 |  | Laos | Oriental | cST |
| 180 | Deltochilini | *Odontoloma* | *sp* |  |  | 2 |  | South Africa | Afrotropical | UPSA |
| 117 | Deltochilini | *Onthobium* | *gutierrezi* | Paulian | (1985) | 1 |  | Australia | Australian | CMNC |
| 28 | Deltochilini | *Paracanthon* | *sp(sp.n)* |  |  | 1 |  | Brazil | Neotropical | cST |
| 30 | Deltochilini | *Pseudonthobium* | *sinuatotibiale* | Montreuil & Théry | 2011 | 1 |  | New Caledonia | Australian | cST |
| 146 | Deltochilini | *Pycnopanelus* | *krikkeni* | Cambefort | 1978 | 2 | 1 | South Africa | Afrotropical | UPSA |
| 24 | Deltochilini | *Saphobius* | *sp* |  |  | 3 | 1 | New Zealand | Australian | cST |
| 9 | Deltochilini | *Scatonomus* | *fasciculatus* | Erichson | 1835 | 1 |  | Brazil | Neotropical | cST |
| 206 | Deltochilini | *Scybalocanthon* | *nigriceps* | Harold | (1868) | 1 |  | Brazil | Neotropical | cST |
| 90 | Deltochilini | *Scybalophagus* | *rugosus* | Blanchard | (1846) | 1 |  | Argentina | Neotropical | CMNC |
| 124 | Deltochilini | *Sylvicanthon* | *bridarollii* | Martínez | 1949 | 1 |  | Peru | Neotropical | CMNC |
| 135 | Deltochilini | *Streblopus* | *opatroides* | Lansberge | 1874 | 1 |  | Brazil | Neotropical | CMNC |
| 212 | Deltochilini | *Tanzanolus* | *sp* |  |  | 1 |  | Tanzania | Afrotropical | CNC |
| 43 | Deltochilini | *Tesserodon* | *erratum* | Storey | 1991 | 2 |  | Australia | Australian | CMNC |
| 213 | Deltochilini | *Tesserodoniella* | *elguetai* | Vaz-de-Mello & Halffter | 2006 | 1 |  | Chile | Neotropical | cST |
| 88 | Deltochilini | *Zonocopris* | *gibbicollis* | Harold | (1868) | 1 |  | Paraguay | Neotropical | CMNC |
| 102 | Dichotomiini | *Aphengium* | *cupreum* | Shipp | 1897 | 1 |  | Brazil | Neotropical | CMNC |
| 2 | Dichotomiini | *Ateuchus* | *squalidus* | Fabricius | 1775 | 1 | 0 | Brazil | Neotropical | cST |
| 107 | Dichotomiini | *Ateuchus* | *histeroides* | Weber | 1801 | 1 |  | USA | Nearctic | CMNC |
| 92 | Dichotomiini | *Bdelyropsis* | *bowditchi* | Paulian | 1939 | 1 |  | Belize | Neotropical | CMNC |
| 93 | Dichotomiini | *Bdelyrus* | *seminudus* | Bates | (1887) | 2 |  | Panama | Neotropical | CMNC |
| 122 | Dichotomiini | *Canthidium* | *sp* |  |  | 1 |  | French Guiana | Neotropical | CMNC |
| 123 | Dichotomiini | *Canthidium* | *bokermanni* | Martinez and Halffter and Pereira | 1964 | 1 |  | Argentina | Neotropical | CMNC |
| 11 | Dichotomiini | *Chalcocopris* | *hesperus* | Olivier | 1789 | 1 |  | Brazil | Neotropical | cST |
| 176 | Dichotomiini | *Coptorhina* | *auspicata* | Peringuey | 1901 | 1 |  | South Africa | Afrotropical | UPSA |
| 207 | Dichotomiini | *Delopleurus* | *sp* |  |  | 1 |  | South Africa | Afrotropical | TMSA |
| 69 | Dichotomiini | *Demarziella* | *interrupta* | Carter | (1936) | 2 |  | Australia | Australian | CMNC |
| 59 | Dichotomiini | *Dichotomius* | sp (near *batesi*) | Harold | (1869) | 1 |  | French Guiana | Neotropical | cST |
| 80 | Dichotomiini | *Dichotomius* | *sericeus* | Harold | (1867) | 1 |  | Brazil | Neotropical | CMNC |
| 150 | Dichotomiini | *Frankenbergerius* | *armatus* | Boheman | (1857) | 1 |  | South Africa | Afrotropical | UPSA |
| 46 | Dichotomiini | *Genieridium* | *margareteae* | Génier & Vaz-de-Mello | 2002 | 1 |  | Brazil | Neotropical | cST |
| 205 | Dichotomiini | *Heliocopris* | *sp* |  |  | 1 |  | Central African Republic | Afrotropical | cST |
| 194 | Dichotomiini | *Homocopris* | *torulosus* | Eschscholtz | 1822 | 2 |  | Chile | Neotropical | cST |
| 151 | Dichotomiini | *Macroderes* | *mutilans* | Kolbe | 1908 | 1 |  | South Africa | Afrotropical | UPSA |
| 86 | Dichotomiini | *Onoreidium* | *howdeni* | Ferreira & Galileo | (1993) | 1 | 1 | Ecuador | Neotropical | CMNC |
| 10 | Dichotomiini | *Ontherus* | *appendiculatus* | Mannerheim | 1829 | 1 |  | Brazil | Neotropical | cST |
| 189 | Dichotomiini | *Paraphytus* | *sp* |  |  | 2 |  | Vietnam | Oriental | CNC |
| 173 | Dichotomiini | *Pedaria* | *sp* |  |  | 1 |  | South Africa | Afrotropical | cST |
| 164 | Dichotomiini | *Sarophorus* | *costatus* | Fahraeus | (1857) | 1 |  | South Africa | Afrotropical | cST |
| 95 | Dichotomiini | *Scatimus* | *strandi* | Balthasar | 1939 | 1 |  | Ecuador | Neotropical | CMNC |
| 101 | Dichotomiini | *Sinapisoma* | *sp* |  |  | 1 |  | French Guiana | Neotropical | CMNC |
| 62 | Dichotomiini | *Trichillum* | *pauliani* | Balthasar | 1939 | 1 |  | Brazil | Neotropical | cST |
| 8 | Dichotomiini | *Uroxys* | *latesulcatus* | Bates | 1891 | 1 |  | Ecuador | Neotropical | cST |
| 98 | Dichotomiini | *Uroxys* | *epipleuralis* | Boucomont | 1928 | 1 | 1 | Brazil | Neotropical | CMNC |
| 127 | Dichotomiini | *Uroxys* | *pauliani* | Balthasar | 1940 | 1 |  | Costa Rica | Neotropical | CMNC |
| 158 | Dichotomiini | *Xinidium* | *dentilabris* | Harold | 1869 | 3 |  | South Africa | Afrotropical | cST |
| 152 | Eucranini | *Ennearabdus* | *lobocephalus* | Harold | 1868 | 1 |  | Argentina | Neotropical | CMNC |
| 39 | Eucranini | *Eucranium* | *arachnoides* | Brulle | 1834 | 1 |  | Argentina | Neotropical | cST |
| 133 | Eucranini | *Glyphoderus* | *monticola* | Burmeister | 1861 | 1 |  | Argentina | Neotropical | CMNC |
| 16 | Eurysternini | *Eurysternus* | *hamaticollis* | Balthasar | 1939 | 2 | 1 | Costa Rica | Neotropical | cST |
| 171 | Gymnopleurini | *Gymnopleurus* | *leei* | Fabricius | (1792) | 2 | 1 | South Africa | Afrotropical | cST |
| 185 | Gymnopleurini | *Paragymnopleurus* | *sp* |  |  | 2 |  | Laos | Oriental | cST |
| 215 | Oniticellini | *Helictopleurus* | *quadripunctatus* | Olivier | (1789) | 1 |  | Madagascar | Afrotropical | cST |
| 214 | Onitini | *Bubas* | *bison* | Linnaeus | (1767) | 1 |  | Spain | Palearctic | cST |
| 218 | Onitini | *Onitis* | *sp* |  |  | 1 |  | Laos | Oriental | cST |
| 216 | Onthophagini | *Onthophagus* | *avocetta* | Arrow | 1933 | 1 |  | Laos | Oriental | cST |
| 55 | Phanaeini | *Coprophanaeus* | *telamon* | Erichson | 1847 | 1 |  | Ecuador | Neotropical | cST |
| 137 | Phanaeini | *Gromphas* | *aeruginosa* | Perty | 1830 | 1 |  | Peru | Neotropical | CMNC |
| 134 | Phanaeini | *Oruscatus* | *davus* | Erichson | 1847 | 1 |  | Peru | Neotropical | CMNC |
| 57 | Phanaeini | *Phanaeus* | *splendidulus* | Fabricius | (1781) | 1 |  | Brazil | Neotropical | cST |
| 76 | Phanaeini | *Tetraechma* | *sanguineomaculata* | Blanchard | (1846) | 1 |  | Argentina | Neotropical | CMNC |
| 54 | Scarabaeini | *Scarabaeus* | *aesculapius* | Olivier | 1789 | 1 |  | ? South Africa | Afrotropical | cST |
| 168 | Scarabaeini | *Scarabaeus* | *aegyptiorum* | Latreille | 1827 | 1 |  | South Africa | Afrotropical | cST |
| 165 | Sisyphini | *Neosisyphus* | *sp* |  |  | 2 | 1 | South Africa | Afrotropical | cST |

# indicates tray ID of glycerin collection in the Zoological Museum, University of Oslo, Norway. The abbreviations for materials deposition are explained in “materials deposition” section of the paper.
